# iRNAm5C-PseDNC: identifying RNA 5-methylcytosine sites by incorporating physical-chemical properties into pseudo dinucleotide composition

**DOI:** 10.18632/oncotarget.17104

**Published:** 2017-04-17

**Authors:** Wang-Ren Qiu, Shi-Yu Jiang, Zhao-Chun Xu, Xuan Xiao, Kuo-Chen Chou

**Affiliations:** ^1^ Department of Computer Science and Bond Life Science Center, University of Missouri, Columbia, MO, USA; ^2^ Computer Department, Jingdezhen Ceramic Institute, Jingdezhen, China; ^3^ Gordon Life Science Institute, Boston, MA, USA; ^4^ Center for Informational Biology, University of Electronic Science and Technology of China, Chengdu, China; ^5^ Center of Excellence in Genomic Medicine Research (CEGMR), King Abdulaziz University, Jeddah, Saudi Arabia

**Keywords:** RNA 5-methylcytosine sites, pseudo dinucleotide composition, physical-chemical property matrix, auto/cross-covariance, web-server

## Abstract

Occurring at cytosine (C) of RNA, 5-methylcytosine (m^5^C) is an important post-transcriptional modification (PTCM). The modification plays significant roles in biological processes by regulating RNA metabolism in both eukaryotes and prokaryotes. It may also, however, cause cancers and other major diseases. Given an uncharacterized RNA sequence that contains many C residues, can we identify which one of them can be of m^5^C modification, and which one cannot? It is no doubt a crucial problem, particularly with the explosive growth of RNA sequences in the postgenomic age. Unfortunately, so far no user-friendly web-server whatsoever has been developed to address such a problem. To meet the increasingly high demand from most experimental scientists working in the area of drug development, we have developed a new predictor called iRNAm5C-PseDNC by incorporating ten types of physical-chemical properties into pseudo dinucleotide composition via the auto/cross-covariance approach. Rigorous jackknife tests show that its anticipated accuracy is quite high. For most experimental scientists’ convenience, a user-friendly web-server for the predictor has been provided at http://www.jci-bioinfo.cn/iRNAm5C-PseDNC along with a step-by-step user guide, by which users can easily obtain their desired results without the need to go through the complicated mathematical equations involved. It has not escaped our notice that the approach presented here can also be used to deal with many other problems in genome analysis.

## INTRODUCTION

**Figure 1:**
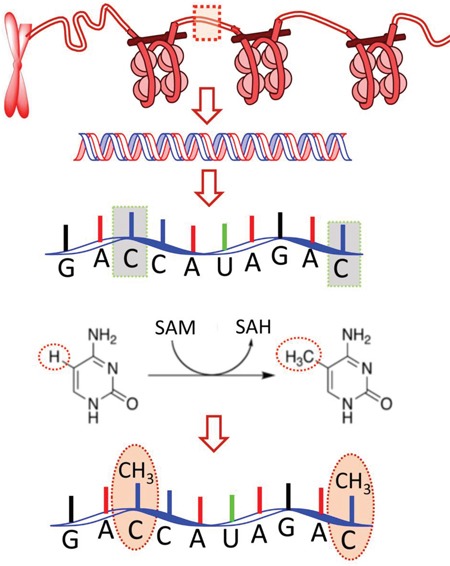
Schematic drawing to show the m^5^C modification in RNA: an important post-transcriptional modification (PTCM) in RNA [4, 5] During the modification process, a methyl group is attached to the 5th atom of the 6-atom ring. SAM and SAH are the abbreviations of *S*-adenosylmethionine and *S*-adenosylhomocysteine, respectively. The former is the source of the methyl group; while the latter, the byproduct.

Post-transcriptional modifications (PTCM) of RNA plays a paramount role for the metabolism processes of RNAs, such as for their splicing export, immune tolerance, and transcription [1–3]. So far, more than 100 distinct PTCMs have been identified in tRNAs, rRNAs, Mt-tRNAs, miRNAs, lincRNAs, miscRNAs, protein-coding genes, pseudogenes, etc. [1]. Among these modifications, the methylation of the 5-methylcytosine (m^5^C) is an epigenetic one [4] formed by the action of RNA methyltransferases [5] (Figure 1). The m^5^C modification is well investigated in DNA, but the corresponding studies in cellular RNA were mainly confined to tRNA and rRNA [6].

Actually, the m^5^C modification site in RNA has various biological functions, including the one that can regulate RNA metabolism in both eukaryotes and prokaryotes [7]. Furthermore, it plays a key role in yeast cell fate decision [4]. It is also significant for animal (such as mouse) and human embryonic development [1].

Although many efforts have been made by using biological experiments to determine the m^5^C sites in RNA (see, e.g. [2, 3]), it is time-consuming and expensive to completely rely on the experimental approaches alone. Facing today's explosive growth of uncharacterized RNA sequences, it is highly demanded to develop computational approach to help getting the information.

Very recently, in a pioneering study, Feng et al. [8] proposed an interesting method to identify RNA m^5^C sites via the powerful PseKNC approach [9–11]. But no web-server has been provided for their method, and hence its practical application value is quite limited. In view of this, the present study was initiated to fill such an empty area.

## RESULTS AND DISCUSSION

A predictor called “iRNAm5C-PseDNC” has been established. The success rates achieved by it on the benchmark dataset constructed based on experimental servations (Supplementary Information 1) are

$$\{\begin{array}{c} \text{Sn}=0.6989\;\\ \begin{array}{c} \text{Sp}=0.9986\;\\ \text{Acc}=0.9237\; \end{array}\\ \text{MCC}=0.7935\; \end{array}$$ (1)

where the definitions for the metrics Sn, Sp, Acc, and MCC are given in Eq.13 of the MATERIALS AND DISCUSSION section later.

Since it is the first web-server predictor ever developed for identifying the m^5^C sites in RNA sequences, it is not possible to demonstrate its power by comparing with its counterparts for exactly the same purpose. Nevertheless, we can indirectly show its power via a cohort of the anticipated success rates (Table 1) reported from the five powerful web-server predictors in genome and proteome analyses [12] [13–16]. As we can see from the table, the iRNAm5C-PseDNC is with the highest score for Acc (see column 3), and the same is true for MCC (column 4), indicating the proposed predictor is not only high in overall accuracy but also quite stable.

**Table 1:** A cohort comparison with some existing web-server predictors for different purposes

Predictor's name	Purpose	Acc^a^	MCC^a^	Sn^a^	Sp^a^
iRSpot-PseDNC^b^	DNA recombination spot	0.8204	0.6380	0.7306	0.8949
iSNO-PseAAC^c^	Cysteine S-nitrosylation site	0.6762	0.3515	0.6701	0.6815
iPro54-PseKNC^d^	Sigma-54 promoter	0.8043	0.6101	0.7702	0.8385
iRSpot-TNCPseAAC^e^	DNA recombination spot	0.8372	0.6710	0.8714	0.7959
iNitro-Tyr^f^	Nitrotyrosine site	0.8452	0.4905	0.8176	0.8598
iRNAm5C-PseDNC^g^	RNA 5-methylcytosine site	0.9237	0.7935	0.6989	0.9986

^a^ See Eq.13 for the definition.

^b^ See ref. [12].

^c^ See ref. [13].

^d^ See ref. [14].

^e^ See ref. [15].

^f^ See ref. [16].

^g^ The web-server predictor developed in this paper.

Also, it is instructive to point out that, among the four metrics in Eq.13, the most important are the Acc and MCC. The metrics Sn and Sp are used to measure a predictor from two completely opposite angles, and they are actually constrained with each other [17]. Therefore, it is meaningless to use only one of the two for comparison [18]. When, and only when, both Sn and Sp of the predictor A are higher than those of the predictor B, can we say A is better than B. In other words, a meaningful comparison in this regard should count the scores of both Sn and Sp, or even better, the rate of their combination that is none but the score of MCC [19, 20].

**Figure 2:**
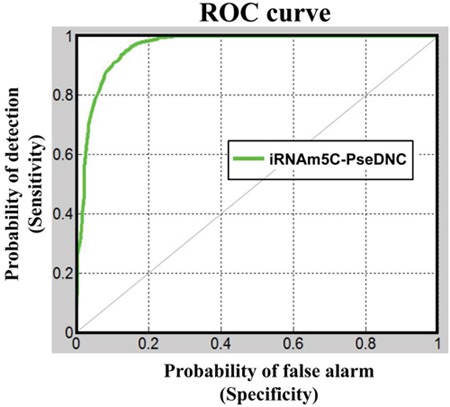
A graphical illustration to show the proposed predictor's performances via the ROC (receiver operating characteristic) curves [29, 30] The area under the ROC curve is called AUC (area under the curve). The greater the AUC value is, the better the performance will be. See the text for further explanation.

Now, let us use graphic analysis to further show the proposed predictor's quality. Graphs are a useful vehicle for studying complicated biological systems because they can provide intuitive insights, as demonstrated by a series of previous studies (see, e.g., [21–28]). To provide an intuitive illustration, the graph of Receiver Operating Characteristic (ROC) [29, 30] was adopted as given in Figure 2, where the green line is the ROC for iRNAm5C-PseDNC. The area under the ROC curve is called the AUC (area under the curve). Being within the region of 0 and 1, the greater the AUC is, the better the predictor would be. For the current predictor, the AUC is 0.9626, which is very close to 1, the value for a perfect predictor.

As shown in a series of recent publications (see, e.g., [18, 19, 31–43]), papers with a user-friendly and publicly accessible web-server will significantly enhance their impacts; this is particularly true for those papers that were aimed at developing various prediction methods [44, 45]. In view of this, the web-server for iRNAm5C-PseDNC has been established at http://www.jci-bioinfo.cn/iRNAm5C-PseDNC. Furthermore, to maximize users’ convenience, a step-to-step guide of how to use it is given in Supplementary Information 2.

## MATERIALS AND METHODS

As practiced in a series of recent studies [19, 20, 35–37, 39–41, 46–53] in complying with the 5-step rule proposed in [54], to establish a really useful sequence-based predictor for a biological system, one should make the following five steps very clear: (1) how to construct or select a valid benchmark dataset to train and test the predictor; (2) how to formulate the biological sequence samples with an effective mathematical expression that can truly reflect their essential correlation with the target concerned; (3) how to introduce or develop a powerful algorithm (or engine) to run the prediction; (4) how to properly conduct cross-validation tests to objectively evaluate the anticipated accuracy; (5) how to provide a web-server and user guide to make people very easily to get their desired results. In the rest of this paper, we are to address these point-by-point.

### Benchmark dataset

To make the description simpler and cleaner, the Chou's sequential scheme, which had been used by many previous investigators for analyzing the enzyme specificity [55], signal peptide cleavage sites [56], nitrotyrosine sites [16, 57], hydroxyproline or hydroxylysine sites [50, 58], methylation sites [34, 39, 59], protein-protein interaction [32], protein-protein binding sites [60, 61], carbonylation sites [48], and phosphorylation sites [51], was adopted in this study. According to Chou's scheme, a potential RNA m^5^C modificationsite sample can be generally expressed by

$$R_{\text{ξ}}(\mathbb{C})={\text{N}}_{-\text{ξ}}{\text{N}}_{-(\text{ξ}-1)}\cdots {\text{N}}_{-2}{\text{N}}_{-1}\mathbb{C}{\text{ N}}_{+1}{\text{N}}_{+2}\cdots {\text{N}}_{+(\text{ξ}-1)}{\text{N}}_{+\text{ξ}}$$ (2)

where the center symbol ℂ denotes the single nucleic acid code cytosine (C), the subscript ξ is an integer, ${\text{N}}_{-\text{ξ}}$ represents the ξ-th upstream nucleotide from the center, the ${\text{N}}_{+\text{ξ}}$ denotes the ξ-th downstream nucleotide, and so forth. The (2ξ+1)-tuple RNA sample $R_{\text{ξ}}(\mathbb{C})$ can be further classified into the following two categories:

$$R_{\xi }(\mathbb{C})\in \{{}_{R_{\xi }^{-}(\mathbb{C}),\;\;\;\text{ otherwise}}^{R_{\xi }^{+}(\mathbb{C}),\;\;\text{ if its center can be of }{\text{m}}^{5}\text{C}}$$ (3)

where $R_{\text{ξ}}^{+}\left(\mathbb{C}\right)$ represents a true ${\text{m}}^{5}\text{C}$ sample with C at its center, $R_{\text{ξ}}^{-}\left(\mathbb{C}\right)$ a false one with C at its center, and the symbol ∈ means “a member of” in the set theory.

In literature the benchmark dataset usually consists of a training dataset and a testing dataset: the former is for training a model, while the latter for testing it. But as elucidated in a comprehensive review [62], there is no need to artificially separate a benchmark dataset into the two parts if the prediction model is tested by the jackknife or subsampling (K-fold) cross-validation because the outcome thus obtained is actually from a combination of many different independent dataset tests. Thus, the benchmark dataset $S_{\text{ξ}}(\mathbb{C})$ for the current study can be formulated as

$$S_{\text{ξ}}=S_{\text{ξ}}^{+}\cup S_{\text{ξ}}^{-}$$ (4)

where the positive and negative subsets, $S_{\text{ξ}}^{+}$ and $S_{\text{ξ}}^{-}$, only contain the true and false m^5^C samples, $R_{\text{ξ}}^{+}\left(\mathbb{C}\right)$ and $R_{\text{ξ}}^{-}\left(\mathbb{C}\right)$, respectively (see Eq.3); while ∪ denotes the symbol of “union” in the set theory [62].

The benchmark dataset used in this study was derived from RMBase [1], which is a resource for decoding the landscape of RNA modifications from high-throughput sequencing data. The detailed procedures are as follows. **(1)** The genomic sequences downloaded from RMBase [63] are in the form of DNA; to make the entire description of this paper in a coherent manner, we first change the code T to U for all the genomic sequences taken from RMBase and make them become RNA sequences. **(2)** As done in [64], by sliding the (2ξ+1)-tuple nucleotide window along each of the RNA sequences thus obtained, collected were only those RNA segments with ℂ= C at the center. (**3**) If the upstream or downstream in a RNA sequence was less than ξ or greater than L−ξ where *L* is the length of the RNA sequence concerned, the lacking code was filled with the same code of its nearest neighbor. (**4**) The RNA segment samples thus obtained were put into the positive subset $S_{\text{ξ}}^{+}$ if their centers were experimentally annotated as the ${\text{m}}^{5}\text{C}$ sites; otherwise, into the negative subset $S_{\text{ξ}}^{-}$. (**5**) To reduce redundancy and bias, none of included RNA segments had pairwise sequence identity with any other in a same subset. By strictly following the above procedures, we obtained an array of benchmark datasets with different ξ values, and hence different lengths of RNA samples as well (see Eq.2), as illustrated below

$$\text{Sample length in }S_{\text{ξ}}=\{\begin{array}{c} \vdots \\ 23\;\text{nucleotides},\text{when ξ}=11\;\\ 25\;\text{nucleotides},\text{when ξ}=12\;\\ \vdots \\ 39\;\text{nucleotides},\text{when ξ}=19\;\\ 41\;\text{nucleotides},\text{when ξ}=20\;\\ \vdots \end{array}$$ (5)

But it was observed via preliminary tests that when ξ=20 (i.e., the RNA samples formed by 41 nucleotides), the corresponding results were most promising. In other words, we observed a turning point for the success rates at ξ=20. After this point, the success rates would become going down with the increase of such parameter. Accordingly, hereafter we only consider the 41-tuple nucleotide samples without explicitly mentioning the parameter ξ any more.

The benchmark dataset S thus obtained is given in Supplementary Information 1, which can also be downloaded at http://www.jci-bioinfo.cn/iRNAm5C/Supp-S1.pdf. It contains 1,900 RNA samples, of which 475 belong to the positive subset $S^{+}$ and 1,425 to the negative subset $S^{-}$.

### Sample formulation

An RNA samples in the aforementioned benchmark dataset can be generally expressed as

$$R={\text{N}}_{1}{\text{N}}_{2}\cdots {\text{N}}_{19}{\text{CN}}_{21}\cdots {\text{N}}_{41}$$ (6)

where ${\text{N}}_{1}$ represents the 1st nucleotide of the RNA sample at its sequence position 1, ${\text{N}}_{2}$ the 2nd nucleotide at its position 2, and so forth. Except for ${\text{N}}_{20}=\text{C}$, they can be any of the four nucleotides; i.e.,

$${\text{N}}_{i}\in \left\{\begin{array}{ccc} \text{A }(\text{adenine}) & \text{C }(\text{cytosine}) & \begin{array}{cc} \text{G }(\text{guanine}) & \text{U }(\text{uracil}) \end{array} \end{array}\right\}$$ (7)

Based on the sequential model of Eq.6, one could directly utilize BLAST to perform statistical analysis. Unfortunately, this kind of straightforward and intuitive approach failed to work when a query RNA sample did not have significant similarity to any of the character-known RNA sequences. To overcome this problem, investigators have shifted their focus to the discrete or vector model. The reason of doing so is also due to the fact that nearly all the existing machine-learning aorithms can be directly used to handle vector models but not sequences, as elaborated in [45].

One of the well-known vector models for DNA/RNA sequences is the *k*-tuple nucleotide (or *k*-mers) composition; i.e.,

$$R={\left[\begin{array}{ccc} f_{1}^{k} & \begin{array}{cc} f_{2}^{k} & f_{3}^{k} \end{array} & \begin{array}{ccc} \cdots & f_{i}^{k} & \begin{array}{cc} \cdots & f_{4^{k}}^{k} \end{array} \end{array} \end{array}\right]}^{T}$$ (8)

where $f_{i}^{k}$ represents the normalized occurrence frequency of the *i*-th *k*-mer, and the symbol **T** is the transpose operator.

When k=1, Eq.8 reduces to

$$R={\left[f\left(\text{A}\right)\;f\left(\text{C}\right)\;f\left(\text{G}\right)\;f\left(\text{U}\right)\right]}^{T}={\left[f_{1}^{1}\;f_{2}^{1}\;f_{3}^{1}\;f_{4}^{1}\right]}^{T}$$ (9)

where $f_{1}^{1}$, $f_{2}^{1}$, $f_{3}^{1}\;$ and $f_{4}^{1}$ are the normalized occurrence frequencies of adenine, cytosine, thymine, and uracil in the RNA sequence, respectively.

When k=2, Eq.8 reduces to

$$R={\left[f\left(\text{AA}\right)\;f\left(\text{AC}\right)\;f\left(\text{AG}\right)\;f\left(\text{AU}\right)\;\cdots \left(\text{TT}\right)\right]}^{T}$$

$$\;\;={\left[f_{1}^{2}\;f_{2}^{2}\;\;f_{3}^{2}\;\;f_{4}^{2}\;\cdots \;f_{16}^{2}\right]}^{T}$$ (10)

where $f_{1}^{2}$ is the normalized occurrence frequencies of AA in the RNA sequence, $f_{2}^{2}$ is that AC, $f_{3}^{2}$ is that AG, $f_{4}^{2}$ is that AU, and so forth.

As we can see from above, the vector's dimension will rapidly increase with the *k* value, causing the so-called “high-dimension disaster” [65] or overfitting problem. This will significantly reduce the deviation tolerance or cluster-tolerant capacity [66], and make the prediction model contain a lot of noise and very unstable.

Therefore, the *k*-mers approach is useful only when the value of *k* is very small. In other words, it can only be used to incorporate the local or short-range or local sequence-order information, but certainly not the long-range or global sequence-order information. To approximately cover the long-range sequence-order effects, one popular and well-known method is to use the pseudo components that were originally introduced in dealing with protein/peptide sequences [67–73] and recently extended to deal with DNA/RNA sequences [9–11, 34, 74–80].

According to the concept of pseudo components, the RNA sequence can be generally formulated by [11, 54]

$$R={\left[{\text{Ψ}}_{1}\;{\text{Ψ}}_{2}\;\cdots \;{\text{Ψ}}_{u}\;\cdots \;\Psi_{\Omega }\right]}^{T}$$ (11)

where the subscript Ω is integer and its value as well as the components ${\text{Ψ}}_{u}\;(u=1,2,\;\cdots ,\;\text{Ω})$ will depend on how to extract the desired information from the RNA sequence of Eq.6.

In this study, we used the approach called “physical-chemical property matrix combined with auto/cross-covariance” proposed by Liu et al. [39] to define the components in Eq.7. According to that approach, the vector components in Eq.7 are given by

$${\text{Ψ}}_{u}=\{\begin{array}{ll} \text{AC}\left(m,\text{λ}\right) & \left(1\leq u\leq 10\text{λ}\right)\\ \text{CC}\left({\text{μ}}_{1},{\text{μ}}_{2},\text{λ}\right) & \left(10\text{λ}+1\leq u\leq 90\;\lambda =\text{Ω}\right) \end{array}$$

$$\left(m=1,\;2,\;\cdots ,\;10;\;{\text{μ}}_{1},\;{\text{μ}}_{2}=1,\;2,\;\cdots ,\;10;\;{\text{μ}}_{1}\neq \;{\text{μ}}_{2}\right)$$ (12)

where λ is an integer within the range from 0 to 39. Using exactly the same calculation approach as elaborated in [39], we found that λ=5 was optimal choice for the current study. As for how to calculate the concrete values in Eq.12, see ref. [39] where a crystal clear description had been given and hence there is no need to repeat here.

### Random forest algorithm

Being a powerful algorithm, the random forest (RF) has been increasingly used to analyze various different problems in computational biology (see, e.g. [32, 36, 37, 40, 48, 50–52, 60, 61, 81–84]). The essence of RF is to compare each individual classifier as a tree, and the combination of many such classifiers as a forest. In this study, 100 trees were used for the forest, and dimension of the random subspace was 22. Each tree in the forest is trained with different part of the benchmark dataset, and hence may yield a different result. The final outcome is determined via a vote from all the trees. For more information about RF, see [85] where a very detailed description has been given, and hence there is no need to repeat here.

The final predictor obtained via the aforementioned procedures is called as iRNAm5C-PseDNC, where “i” stands for “identify”, and “RNAm5C” for “RNA 5-methylcytosine modification sites”, and “PseDNC” for “pseudo dinucleotide composition”.

### Test procedure

One of the important procedures [54] in developing a new prediction method is how to objectively evaluate its anticipated success rate [54]. To address this, we need to consider two issues. (1) What metrics should be used to quantitatively reflect the predictor's quality? (2) What kind of test approach should be utilized to score the metrics?

### Metrics formulation

The following metrics are generally used to measure the prediction quality from four different angles: (1) Acc for measuring the overall accuracy of a predictor, (2) MCC for its stability, (3) Sn for its sensitivity, and (4) Sp for its specificity [86]. Unfortunately, their conventional formulations as given in [86] lack intuitiveness and most experimental scientists feel difficult to understand them, particularly for the MCC. Interestingly, using the Chou's symbols introduced in studying signal peptides [56], Xu et al. [13] and Chen et al. [12] converted them into a set of four intuitive equations, as given by

$$\{\begin{array}{lll} \text{Sn}=1-\frac{N_{-}^{+}}{N^{+}} & & \;0\;\leq \text{Sn}\leq 1\\ \text{Sp}=1-\frac{N_{+}^{-}}{N^{-}} & & \;0\leq \text{Sp}\leq 1\\ \text{Acc}=1-\frac{N_{-}^{+}+N_{+}^{-}}{N^{+}+N^{-}} & & \;0\leq \text{Acc}\leq 1\\ \text{MCC}=\;\frac{1-\left(\frac{N_{-}^{+}}{N^{+}}+\frac{N_{+}^{-}}{N^{-}}\right)}{\sqrt{\left(1+\frac{N_{+}^{-}-N_{-}^{+}}{N^{+}}\right)\;\left(1+\frac{N_{-}^{+}-N_{+}^{-}}{N^{-}}\right)}} & & -1\leq \text{MCC}\leq 1 \end{array}$$ (13)

where $N^{+}$ represents the total number of the true m^5^C sites investigated, while $N_{-}^{+}$ is the number of the true m^5^C sites incorrectly predicted to be of false m^5^C site; $N^{-}$ is the total number of the false m^5^C sites investigated, while $N_{+}^{-}$ is the number of the false m^5^C sites incorrectly predicted to be of true m^5^C site.

According to Eq.13, it is crystal clear to see the following. When $N_{-}^{+}=0$ meaning none of the true m^5^C sites are incorrectly predicted to be of false m^5^C site, we have the sensitivity Sn=1. When $N_{-}^{+}=N^{+}$ meaning that all the true m^5^C sites are incorrectly predicted to be of false m^5^C site, we have the sensitivity Sn=0. Likewise, when $N_{+}^{-}=0$ meaning none of the false m^5^C sites are incorrectly predicted to be of m^5^C site, we have the specificity Sp=1; whereas $N_{+}^{-}=N^{-}$ meaning that all the false m^5^C sites are incorrectly predicted to be of true m^5^C sites, we have the specificity Sp=0. When $N_{-}^{+}=N_{+}^{-}=0$ meaning that none of true m^5^C sites in the positive dataset and none of the false m^5^C sites in the negative dataset are incorrectly predicted, we have the overall accuracy Acc=1 and MCC=1; when $N_{-}^{+}=N^{+}$ and $N_{+}^{-}=N^{-}$ meaning that all the true m^5^C sites in the positive dataset and all the false m^5^C sites in the negative dataset are incorrectly predicted, we have the overall accuracy Acc=0 and MCC=−1; whereas when $N_{-}^{+}=N^{+}/2$ and $N_{+}^{-}=N^{-}/2$ we have Acc=0.5 and MCC=0 meaning no better than random guess. Therefore, Eq.13 has made the meanings of sensitivity, specificity, overall accuracy, and stability much more intuitive and easier-to-understand, particularly for the meaning of MCC, as concurred recently by many investigators (see, e.g., [18, 20, 31–35, 47–52, 60, 75–77, 84, 87–92]).

Note that, however, the set of equations defined in Eq.13 is valid only for the single-label systems. For the multi-label systems whose emergence has become more frequent in system biology [93–95] and system medicine [96] or biomedicine [40], a completely different set of metrics are needed as elaborated in [97].

### Test method

Now let us discuss what kind of test method should be used to score the four metrics in Eq.13. In statistical analysis, the following three methods are often used to test a predictor: (1) independent dataset test, (2) subsampling (or K-fold cross-validation) test, and (3) jackknife test [98]. Of these three, however, the jackknife test is deemed the least arbitrary that can always yield a unique outcome for a given benchmark dataset as elucidated in [54]. Accordingly, the jackknife test has been widely recognized and increasingly used by investigators to examine the quality of various predictors (see, e.g., [15, 99–108]).

Accordingly, here we also used the jackknife test to check the quality of iRNAm5C-PseDNC predictor. During the jackknifing process, both the training dataset and testing dataset are actually open, and each sample will be in turn moved between the two. The jackknife test can exclude the “memory” effect. Also, the arbitrariness problem [54] rooted in the independent dataset and subsampling tests can be completely avoided because the outcome obtained by the jackknife cross-validation is always unique for a given benchmark dataset.

## SUPPLEMENTARY MATERIALS FIGURES AND TABLES




