# Supplementary material for: iRNAm5C-PseDNC: identifying RNA 5-methylcytosine sites by incorporating physical-chemical properties into pseudo dinucleotide composition
Source: Oncotarget. 2017 Apr 17;8(25):41178–88. doi: 10.18632/oncotarget.17104 (PMC5522291; doi:10.18632/oncotarget.17104)
Supplement: Supplementary file 1 [file oncotarget-08-41178-s001.pdf]

iRNAm5C-PseDNC: identifying RNA 5-methylcytosine sites by incorporating physical-chemical properties into pseudo dinucleotide composition

SUPPLEMENTARY MATERIALS

SUPPLEMENTARY INFORMATION

Supplementary Information 1: The benchmark dataset $\mathbb{S}_{\xi=20}$ used to train and test the model for predicting the possibility of 5-methylcytosine site. It contains 475 positive samples and 1425 negative samples. See the main text for further explanation.

See Supplementary File 1

Supplementary Information 2: User Guide for the Web-Server iRNAm5C-PseDNC

To maximize users' convenience, a step-to-step guide for how to use the web-server to get the desired results is given below.

Step 1. Open the web-server at http://www.jci-bioinfo.cn/iRNAm5C-PseDNC and you will see the top page of the iRNAm5C-PseDNC on your computer screen, as shown in Supplementary Figure 1. Click on the Read Me button to see a brief introduction about the iRNAm5C and the caveat when using it.

Step 2. Either type or copy/paste the query RNA sequences into the input box at the center of Supplementary Figure 1. The input sequence should be in the FASTA format. For the examples of sequences in FASTA format, click the Example button right above the input box.

Step 3. Click on the Submit button to see the predicted result. For example, if you use the two query DNA sequences in the Example window as the input and, after 15 seconds or so, you will see the following shown on the screen of your computer: (1) Example_01 contains 20 C nucleotide residues, of which 3 are predicted to be 5-methylcytosine sites and they are at sequence position 58, 98, and 99. (2) Example_02 contains 20 C residues, of which 4 are predicted to be 5-methylcytosine sites and they are at sequence position 21, 62, 102, and 103. All these results are fully consistent with experimental observations.

Step 4. As shown on the lower panel of Supplementary Figure 1, you may also choose the batch prediction by entering your e-mail address and your desired batch input file (in FASTA format of course) via the Browse button. To see the sample of batch input file, click on the button Batch-example. After clicking the button Batch-example, you will see "Your batch job is under computation; once the results are available, you will be notified by e-mail."

Step 5. Click the Supplementary Information button to download the benchmark dataset used to train and test the iRNAm5C-PseDNC predictor.

Step 6. Click on the Citation button to find the relevant papers that document the detailed development and algorithm of iRNAm5C-PseDNC.

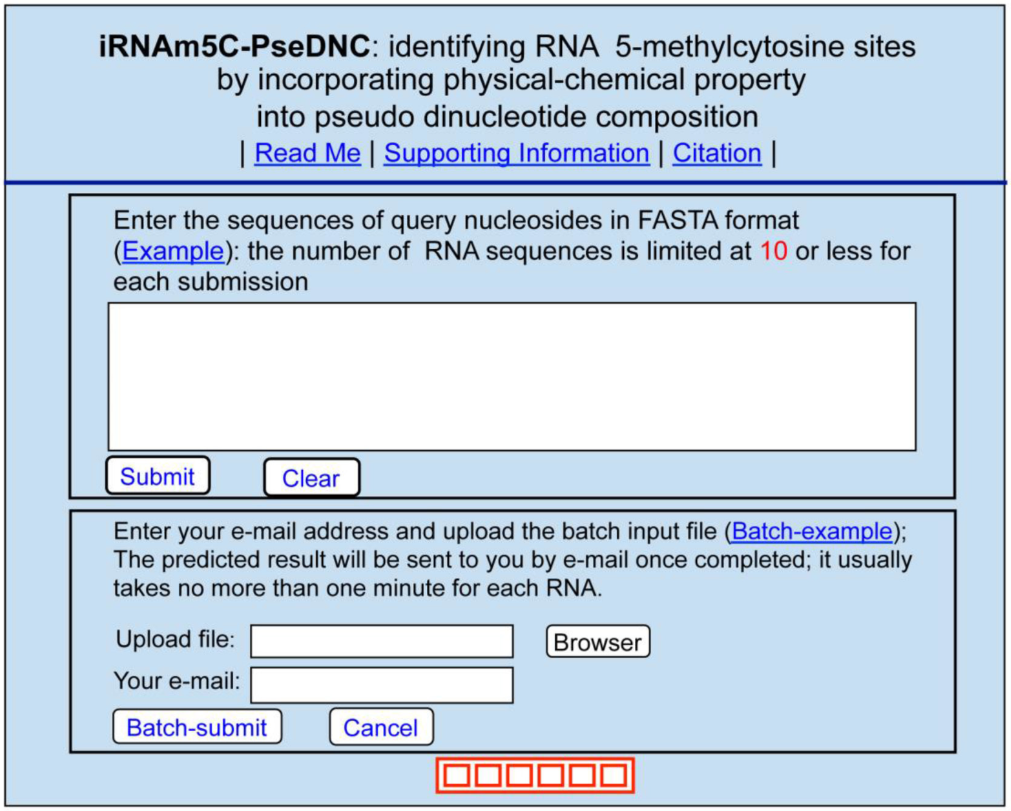

Supplementary Figure 1: A semi-screenshot for the top-page of the iRNAm5C-PseDNC web-server at http://www.jci-bioinfo.cn/iRNAm5C-PseDNC.
