# Supplementary material for: iRNAm5C-PseDNC: identifying RNA 5-methylcytosine sites by incorporating physical-chemical properties into pseudo dinucleotide composition
Source: Oncotarget. 2017 Apr 17;8(25):41178–88. doi: 10.18632/oncotarget.17104 (PMC5522291; doi:10.18632/oncotarget.17104)
Supplement: Supplementary file 2 [file oncotarget-08-41178-s002.docx]

**Supplementary Information 1**. The benchmark dataset $\mathbb{S}_{\xi=20}$used to train and test the model for predicting the possibility of 5-methylcytosine site. It contains 475 positive samples and 1425 negative samples. See the main text for further explanation.

# List of the 475 RNA samples in the positive subset $\mathbb{S}^{\mathbf{+}}$

UCGCCUCCCACGCGGGAGAC**C**CGGGUUCAAUUCCCGGCCAA

CUCGCCUCCCACGCGGGAGA**C**CCGGGUUCAAUUCCCGGCCA

CCGGGUUCAAUUCCCGGCCA**C**UGCACGUGGUUGUUUUUCAC

CGCCUCCCAUGCGGGAGACC**C**GGGUUCAAUUCCCGGCCACU

UCGCCUCCCAUGCGGGAGAC**C**CGGGUUCAAUUCCCGGCCAC

GGCCGUGGGUGUGUAGAGGC**C**UUGGUGGUGCAGUGGUAGAA

GUGCAGUGGUAGAAUUCUCG**C**CUCCCACGUGGGAGACCCGG

UCGCCUCCCACGUGGGAGAC**C**CGGGUUCAAUUCCCGGCCAA

CGCCUCCCACGUGGGAGACC**C**GGGUUCAAUUCCCGGCCAAU

CGCCUCCCACGCGGGAGACC**C**GGGUUCAAUUCCCGGCCAAU

UGACUGCAGAUCAAGAGGUC**C**CCGGUUCAAAUCCGGGUGCC

GCUGAAGGCAUUCAAAGGUU**C**CGGGUUCGAGUCCCGGCGGA

CUGAAGGCAUUCAAAGGUUC**C**GGGUUCGAGUCCCGGCGGAG

GCAUUCAAAGGUUCCGGGUU**C**GAGUCCCGGCGGAGUCGUAA

AGAUGUUGGUGGUAGUAGCA**C**AUAUUCUAAAGAGAACUUCA

AGCCCAGGGCUGGGCCCGGG**C**AGAGCCGCCGCAGGUGCAGA

CUGCGGUGAGUCUUGAAGCC**C**AGGGCUGGGCCCGGGCAGAG

UAUUUGAUCGGAUCGUGACC**C**CAGCCCCGCCGGGCCGACCC

CGCUGUGGUCCCCGAGGUCC**C**GGAGCUGGCCCUGCGGGGCC

GAGACUCUUAAUCUCAGGGU**C**GUGGGUUCGAGCCCCACGUU

AGACGCCAGUAAUCAGUGCC**C**GGCUAGCUCAGUCGGUAGAG

AAGACGCCAGUAAUCAGUGC**C**CGGCUAGCUCAGUCGGUAGA

GGUUCGAAUCCGAGUCACGG**C**AGGUGGUUCUAACUUGCUGG

CUCGGUUCGAAUCCGAGUCA**C**GGCAGGUGGUUCUAACUUGC

UGCGUUGUGGCCGCAGCAAC**C**UCGGUUCGAAUCCGAGUCAC

CUGCGUUGUGGCCGCAGCAA**C**CUCGGUUCGAAUCCGAGUCA

GCGCCGCCUGGUUAGUGGCU**C**GCCGUGAUCGUAUAGUGGUU

GCGCGCCGCCUGGUUAGUGG**C**UCGCCGUGAUCGUAUAGUGG

UGCCUUCCAAGCAGUUGACC**C**GGGUUCGAUUCCCGGCCAAC

CUGCCUUCCAAGCAGUUGAC**C**CGGGUUCGAUUCCCGGCCAA

GCUGCCUUCCAAGCAGUUGA**C**CCGGGUUCGAUUCCCGGCCA

GCGCUCUCACCGCCGCGGCC**C**GGGUUCGAUUCCCGGUCAGG

GGCGCUCUCACCGCCGCGGC**C**CGGGUUCGAUUCCCGGUCAG

GCCUCACACGCGAAAGGUCC**C**CGGUUCGAAACUGGGCGGAA

CGCCUCACACGCGAAAGGUC**C**CCGGUUCGAAACUGGGCGGA

UCGCCUCACACGCGAAAGGU**C**CCCGGUUCGAAACUGGGCGG

CAGGUUCCGCCUGUGGUUUC**C**GUAGUGUAGUGGUUAUCACG

AGCGUGCUGGGCCCAUAACC**C**AGAGGUCGAUGGAUCGAAAC

UGGGCCCAUAACCCAGAGGU**C**GAUGGAUCGAAACCAUCCUC

GUCGAUGGAUCGAAACCAUC**C**UCUGCUAUCGGAGUUUUUUC

GGACUUCUAAUCCAGAGGUU**C**CGGGUUCGAGUCCCGGCAGA

GAAAGGUCCCCGGUUCGAAA**C**CGGGCGGAAACAAAGUGGUU

CGCCUCACACGCGAAAGGUC**C**CCGGUUCGAAACCGGGCGGA

UCGCCUCACACGCGAAAGGU**C**CCCGGUUCGAAACCGGGCGG

GCGCUUUCACCGCCGCGGCC**C**GGGUUCGAUUCCCGGUCAGG

GGCGCUUUCACCGCCGCGGC**C**CGGGUUCGAUUCCCGGUCAG

UGCCUUCCAAGCAGUUGACC**C**GGGCUCGAUUCCCGCCCAAC

UUGCCUUCCAAGCAGUUGAC**C**CGGGCUCGAUUCCCGCCCAA

UUGCCUGCCACGCAGGAGGC**C**CAGGUUUGAUUCCUGGCCCA

CGCCUGCCACGCGGGAGGCC**C**GGGUUCGAUUCCCGGCCAAU

UCGCCUGCCACGCGGGAGGC**C**CGGGUUCGAUUCCCGGCCAA

CUCGCCUGCCACGCGGGAGG**C**CCGGGUUCGAUUCCCGGCCA

GGUAGAAUUCUCGCCUGCCA**C**GCGGGAGGCCCGGGUUCGAU

GUCGCAGUCUCCCCUGGAGG**C**GUGGGUUCGAAUCCCACUCC

AGUGCCCUUGCCGAGAGCGG**C**UCGUUGGUCUAGGGGUAUGA

CGCUUCGGGUGCGAGAGGUC**C**CGGGUUCAAAUCCCGGACGA

GCUUCGGGUGCGAGAGGUCC**C**GGGUUCAAAUCCCGGACGAG

CUUCGGGAGCGCCCGGAUAG**C**UCAGUCGGUAGAGCAUCAGA

CAGACUUUUAAUCUGAGGGU**C**CAGGGUUCAAGUCCCUGUUC

AGACUUUUAAUCUGAGGGUC**C**AGGGUUCAAGUCCCUGUUCG

GUCUACGGGAGCCCGGAUAG**C**UCAGUCGGUAGAGCAUCAGA

UUAGUACUUGGAUGGGAGAC**C**GCCUGGGAAUACCUGGUGCU

GUUAGUACUUGGAUGGGAGA**C**CGCCUGGGAAUACCUGGUGC

AACACGCCAGAUCUCGGAAA**C**UAAGCAGGGUCGGGCCUGGU

AGACUUUUAAUCUGAGGGUC**C**AGGGUUCAAGUCCUCGUUCG

UGGUCUUGUAAACCAGGGGU**C**GCGAGUUCGAUCCUCGCUGG

GUUAGUACUUAACUGGGAGA**C**CGCCUGGGAAUAACGGGUGC

UUAGUACUUAACUGGGAGAC**C**GCCUGGGAAUAACGGGUGCU

UUAGUACUUGGACGGGAGAC**C**GCCUGGGAAUACCGGGUGCU

GUUAGUACUUGGACGGGAGA**C**CGCCUGGGAAUACCGGGUGC

GAAGCUAAGCAGGGUCGGGC**C**UGGUUAGUACUUGGACGGGA

AUCUCGUCUGAUCUCGGAAG**C**UAAGCAGGGUCGGGCCUGGU

UGGCCGAGUGGUCUAAGGCG**C**CAGACUCAAGGUAAGCACCU

GUGGUCUAAGGCGCCAGACU**C**AAGGUAAGCACCUUGCCUGC

UUCUGGUCUCCGGAUGGAGG**C**GUGGGUUCGAAUCCCACUUC

CGGCGCUCUCACCGCCGCGG**C**CCGGGUUCGAUUCCCGGUCA

CCAUACCACCCUGAACGCGC**C**CAAUCUCGUCUGAUCUCGGA

AUCUCUGUCUACGGCCAUAC**C**ACCCUGAACGCGCCCAAUCU

UGCUUUACACGCAGAAGGUC**C**UGGGUUCAAGCCCCAGUGGA

CUGCUUUACACGCAGAAGGU**C**CUGGGUUCAAGCCCCAGUGG

AAUCCAAUGGGGUCUCCCCG**C**GCAGGUUCGAACCCUGCUCG

GUAAGCAGGGUCGGGCCUGG**C**UAGUACUUUGAAGGAAGCAG

CUGCUGCCGUGAUCGUAUAG**C**GGUUAGUAGUCUGCGUUGUG

UUAGUACUUGGAUGGGAGAC**C**UCCUGGGAAUACCGGGUGCU

GUUAGUACUUGGAUGGGAGA**C**CUCCUGGGAAUACCGGGUGC

GUGGAACCAUGAGAUGUUAC**C**UAGCGUUUUGUGAGCCAGGU

UGCUUUACACGCAGAAGGUC**C**UGGGUUCGAGCCCCAGUGGA

CUGCUUUACACGCAGAAGGU**C**CUGGGUUCGAGCCCCAGUGG

AAGGCGUUGGACUUAAGAUC**C**AAUGGAUUCAUAUCCGCGUG

AUCCAAUGGAUUCAUAUCCG**C**GUGGGUUCGAACCCCACUUC

CAGACUUUUAAUCUGAGGGU**C**CGGGGUUCAAGUCCCUGUUC

AGACUUUUAAUCUGAGGGUC**C**GGGGUUCAAGUCCCUGUUCG

AGACUGAAGAUCUAAAGGUC**C**CUGGUUCGAUCCCGGGUUUC

UAGACUGAAGAUCUAAAGGU**C**CCUGGUUCGAUCCCGGGUUU

AAAUAGCUCAGUUGGGAGAG**C**GUUAGACUGAAGAUCUAAAG

AGACUGAAGAUCUAAAGGUC**C**CUGGUUCAAUCCCGGGUUUC

UAGACUGAAGAUCUAAAGGU**C**CCUGGUUCAAUCCCGGGUUU

AAUCCAUUGUGCUUUGCACG**C**GUGGGUUCGAAUCCCAUCCU

GGUUUGGGUCCGAGAGGUCC**C**GGGUUCAAAUCCCGGACGAG

CGGUUUGGGUCCGAGAGGUC**C**CGGGUUCAAAUCCCGGACGA

AGACUUUUAAUCUGAGGGUC**C**AGGGUUCAAGUCCCUGUUCA

AUCUCGUCUGAUCUCGGAAG**C**UAAGGAGGGUCGGGCCUGGU

GUGCCUCUCAUGUACAAGGC**C**CUGAGUUUGACUCCCAGCAC

GUACUUGGAAGGGAGACUGA**C**UGGGAAUACCGGGUGCUGUA

UCAGUGCUUGGAAGGGAGAC**C**GCCUGGGAAUACCGGGUGCU

AAGGCGUUGGACUCGAAAUC**C**AAUGGGGUUUCCCCGCACAG

AAUCCAAUGGGGUUUCCCCG**C**ACAGGUUCGAAUCCUGUUCG

CCCGCCUGUCACGCGGGAGA**C**CGGGGUUCGAUUCCCCGACG

CCGCCUGUCACGCGGGAGAC**C**GGGGUUCGAUUCCCCGACGG

CCCGCCUGUCACGCGGGAGA**C**CGGGGUUCAAUUCCCCGACG

CCGCCUGUCACGCGGGAGAC**C**GGGGUUCAAUUCCCCGACGG

CCCGACGGGGAGGUGUGUAG**C**UGCACUUUUUUGGCGACAGU

GACGGGGAGGUGUGUAGCUG**C**ACUUUUUUGGCGACAGUUAU

CUGCGCGAGCCACAGCCCAG**C**AGGACCUCGUGGCGCAACGG

GCCACAGCCCAGCAGGACCU**C**GUGGCGCAACGGUAGCGCGU

AACGGUAGCGCGUCUGACUC**C**AGAUCAGAAGGCUGCGUGUU

UCUGACUCCAGAUCAGAAGG**C**UGCGUGUUCGAAUCACGUCG

UGCUUUGCAUGUAUGAGGCC**C**CGGGUUCGAUCCCCGGCAUC

CCCGACGGGGAGGCCAAGUA**C**GUUUUUACCAUUCUUCCGUA

AGUAUAGUGGUGAGUAUCCC**C**GCCUGUCACGCGGGAGACCG

CGUAACGGCUGCCGAAAUAG**C**UCAGUUGGGAGAGCGUUAGA

AUGCUUUGCACGUAUGAGGC**C**CCGGGUUCAAUCCCCGGCAU

UGCUUUGCACGUAUGAGGCC**C**CGGGUUCAAUCCCCGGCAUC

AGGUGGCCCGGGUUCGACUC**C**CGGUAUGGGAACGCUUCCUU

UGGUUUUCACCCAGGUGGCC**C**GGGUUCGACUCCCGGUAUGG

CUGGUUUUCACCCAGGUGGC**C**CGGGUUCGACUCCCGGUAUG

CCUGGUUUUCACCCAGGUGG**C**CCGGGUUCGACUCCCGGUAU

UGGUCUAGCGGUUAGGAUUC**C**UGGUUUUCACCCAGGUGGCC

UGGUUUUCACCCAGGCGGCC**C**GGGUUCGACUCCCGGUGUGG

CUGGUUUUCACCCAGGCGGC**C**CGGGUUCGACUCCCGGUGUG

UGGUCUAGCGGUUAGGAUUC**C**UGGUUUUCACCCAGGCGGCC

AUGGUCUAGCGGUUAGGAUU**C**CUGGUUUUCACCCAGGCGGC

CCGCGCGAGGUCAGACUGGG**C**AGGAGAUGCCGUGGACCCCG

GCUUAGGGUGCGAGAGGUCC**C**GGGUUCAAAUCCCGGACGAG

GGCUCCAGUCUCUUCGGGGG**C**GUGGGUUCGAAUCCCACCGC

CGCUUAGGGUGCGAGAGGUC**C**CGGGUUCAAAUCCCGGACGA

UAAGAGUAUCUGUAUCGCGG**C**UCGUUGGUCUAGGGGUAUGA

ACUUAAGAGUAUCUGUAUCG**C**GGCUCGUUGGUCUAGGGGUA

UGGUCUUGUAAACCAGGGGU**C**GCGAGUUCAAUUCUCGCUGG

GGCUCCAGUCUCUUCGGGGG**C**GUGGGUUCGAAUCCCACCAC

UGGUCUUGUAAACCAGGGGU**C**GCGAGUUCAAAUCUCGCUGG

CGCUUUGGGUGCGAGAGGUC**C**CGGGUUCAAAUCCCGGACGA

GCUUUGGGUGCGAGAGGUCC**C**GGGUUCAAAUCCCGGACGAG

AUUUGUGGACAUCCUUAGGU**C**GCUGGUUCGAUUCCGGCUCG

GUAGAGCGGAGGACUGUAGC**C**UGUAGAAACAUUUGUGGACA

AUUUGCGGACAUCCUUAGGU**C**GCUGGUUCGAUUCCAGCUCG

GUUUGUGGACAUCCUUAGGU**C**GCUGGUUCAAUUCCGGCUCG

CAGUGUGCAUCCUUCGAUAG**C**UCAGCUGGUAGAGCGGAGGA

AUUUGCGGACAUCCUUAGGU**C**GCUGGUUCGAUUCCGGCUCG

CAGCGUGCACCCUUCGAUAG**C**UCAGCUGGUAGAGCGGAGGA

UUCUGCGUGAGGCCCUAUAG**C**UCAGGGGUUAGAGCACUGGU

AUGUGUGGUCAUCCUUAGGU**C**GCUGGUUCGAUUCCGGCUCG

GCACCACUCAGCUACAGUGG**C**UCGUUGGUCUAGGGGUAUGA

UCGCUUUGGGUGCGAGAGGU**C**CCGGGUUCAAAUCCCGGACG

UGACUACGGAUCAGAAGAUU**C**CAGGUUCGACUCCUGGCUGG

GGGAUCGCGCCUGUGAAUAG**C**CACUGCACUCCAGCCUGGGC

GGAUCGCGCCUGUGAAUAGC**C**ACUGCACUCCAGCCUGGGCA

CUGGGCAACAUAGCGAGACC**C**CGUCUCUUUUGAACAAUAAA

CUGAGCAACAUAGCGAGACC**C**CGUCUCUUUUGCCCCCCUCC

UGUGAAUAGCCACUGCACUC**C**AGCCUGAGCAACAUAGCGAG

GGAUCGCGCCUGUGAAUAGC**C**ACUGCACUCCAGCCUGAGCA

GGGAUCGCGCCUGUGAAUAG**C**CACUGCACUCCAGCCUGAGC

GGGACUCUUAAUCCCAGGGU**C**GUGGGUUCGAGCCCCACGUU

AGGCUAACGUAACAGGCGCC**C**GGCUAGCUCAGUCGGUAGAG

GGUGCUAAUAACGCCAAGGU**C**GCGGGUUCGAUCCCCGUACG

CCUGGUUUUCACCCAGGCGG**C**CCGGGUUCGACUCCCGGUGU

AACGGUUACUUGUCAUCUCC**C**ACAUGGUCUAGCGGUUAGGA

CAACGGUUACUUGUCAUCUC**C**CACAUGGUCUAGCGGUUAGG

AAUCCAUUGUGCUCUGCACG**C**GUGGGUUCGAAUCCCAUCCU

GGUUCGAAUCCGAGUCACGG**C**AUUGUGGGAACAAUGGCACG

GUAUAGUGGUUAGUACUCUG**C**GUUGUGGCCGCAGCAACCUC

GGUUCGAAUCCGAGUCACGG**C**AUUGUGAGGACAAUGGCACG

GGUUCGAAUCCGAGUCACGG**C**AUUGUGGAAACAAUGGUACG

GGACUCUGAAUCCAGCGAUC**C**GAGUUCAAAUCUCGGUGGAA

UGGACUCUGAAUCCAGCGAU**C**CGAGUUCAAAUCUCGGUGGA

UUGACUGCAGAUCAAGAGGU**C**CCCGGUUCAAAUCCGGGUGC

CCGAGGCCGCCCAGAGCCCU**C**CCCGGGAGACCCGAGGCCGC

CCGAGGCCGCCCAGGGCCCU**C**CCCGGGAGACCCGAGGCCGC

CCCGGGGAGACCCGAGGCCG**C**CCAGGGCCCUCCCGGGAGAC

UCCCGGGAGACCCGAGGCCG**C**CCAGGGCCCUCCCGGGAGAC

AGAUUCCCAUUCUUGCGACC**C**GGGUUCGAUUCCCGGGCGGC

AAGAUUCCCAUUCUUGCGAC**C**CGGGUUCGAUUCCCGGGCGG

GAGACCCUUAAUCUCAGGGU**C**GUGGGUUCGAGCCCCACGUU

CAGUCGGUAGAGCAUGAGAC**C**CUUAAUCUCAGGGUCGUGGG

AGGUAACGUUGGCGUGUCGC**C**CGGCUAGCUCAGUCGGUAGA

GGUAACGUUGGCGUGUCGCC**C**GGCUAGCUCAGUCGGUAGAG

GAGACUCUUAAUCUCAGGGU**C**GUGGGUUCGAGCCGCACGUU

AAAACGUUCCCAGGGAAGCC**C**GGCUAGCUCAGUCGGUAGAG

GGGACUCUUAAUCUCAGGGU**C**GUGGGUUCGAGCCCCACGUU

CGCUUAGGAUGCGAGAGGUC**C**CGGGUUCAAAUCCCGGACGA

GCUUAGGAUGCGAGAGGUCC**C**GGGUUCAAAUCCCGGACGAG

GAUGCGAGAGGUCCCGGGUU**C**AAAUCCCGGACGAGCCCCUC

CGGUCUCGUAAACCGAAGAU**C**ACGGGUUCGAACCCCGUCCG

GGCUCCAGUCAUUUCGAUGG**C**GUGGGUUCGAAUCCCACCGC

UGGCCGAGUGGUCUAAGGCG**C**UGGAUUUAGGCUCCAGUCAU

AACAUAUUGCAGCUGGGUAG**C**GUGGCCGAGCGGUCUAAGGC

GUCGCAGUCUCCCCUGGAGG**C**GUGGGUUCGAAUCCCACUUC

CAGACCAGUCUACGGCCAUA**C**CACCCUGAACGCGCCGAUCU

GAAGCUAAGCAGGGUCGGGC**C**UGGUUAGUACUAGUACUUGG

CGCCUGCCACGCGGGAGGCC**C**GGGUUUGAUUCCCGGCCAGU

UCGCCUGCCACGCGGGAGGC**C**CGGGUUUGAUUCCCGGCCAG

CUCGCCUGCCACGCGGGAGG**C**CCGGGUUUGAUUCCCGGCCA

UAGAAUUCUCGCCUGCCACG**C**GGGAGGCCCGGGUUUGAUUC

GGUAGAAUUCUCGCCUGCCA**C**GCGGGAGGCCCGGGUUUGAU

GGCCCGGGUUCGAUUCCCGG**C**CAAUGCAGCAGCUGAAAGCU

AUUCUCGCCUGCCAUGCGGG**C**GGCCGGGCUUCGAUUCCUGG

CAGUCUCAUAAUCUGAAGGU**C**CUGAGUUCGAGCCUCAGAGA

AGUCUCAUAAUCUGAAGGUC**C**UGAGUUCGAGCCUCAGAGAG

CAGGGAAGAAGCCUGGAUAG**C**UCAGUUGGUAGAGCAUCAGA

CAGUCUCAUAAUCUGAAGGU**C**GUGAGUUCGAGCCUCACACG

UGAAUCCAGCGAUCCGAGUU**C**AAAUCUCGGUGGAACCUUGA

GGCUCCAGUCUCUUCGGAGG**C**GUGGGUUCGAAUCCCACCGC

AAUCCAAUGGGGUCUCCCCG**C**GCAGGUUCGAAUCCUGCUCA

AAGGCGUUGGACUCGAAAUC**C**AAUGGGGUCUCCCCGCGCAG

UGUCUAGUAAACAGGAGAUC**C**UGGGUUCGAAUCCCAGCGGU

CUGUCUAGUAAACAGGAGAU**C**CUGGGUUCGAAUCCCAGCGG

UGCUAAUAACGCCAAGGUCG**C**GGGUUCGAUCCCCGUACGGG

UGGUAUAGUGGUAAGCAUAG**C**UGCCUUCCAAGCAGUUGACC

AAUCCAUUGGGGUCUCCCCG**C**GCAGGUUCGAAUCCUGCCGA

GACUCCAGAUCAGAAGGUUG**C**GUGUUCAAGUCACGUCGGGG

UAGAAUUCUCGCCUCCCACG**C**AGGAGACCCAGGUUCGAUUC

CGGUCUCGUAAACCGAAGAU**C**GCGGGUUCGAACCCCGUCCG

UGACUGCAGAUCAAGAAGUC**C**CCGGUUCAAAUCCGGGUGCC

UUGACUGCAGAUCAAGAAGU**C**CCCGGUUCAAAUCCGGGUGC

UGACUGCAGAUCAAGAGGUC**C**CUGGUUCAAAUCCGGGUGCC

UUGACUGCAGAUCAAGAGGU**C**CCUGGUUCAAAUCCGGGUGC

CUUAAGAAGCCAGCAGGUCC**C**AUGGUGUAAUGGUUAGCACU

UCCCAUGGUGUAAUGGUUAG**C**ACUCUGGACUUUGAAUCCAG

UGGACUUUGAAUCCAGCGAU**C**CGAGUUCAAAUCUCGGUGGG

GGACUUUGAAUCCAGCGAUC**C**GAGUUCAAAUCUCGGUGGGA

UGAAUCCAGCGAUCCGAGUU**C**AAAUCUCGGUGGGACCUCAC

CUAGGCAGGGUCAGGCCUGG**C**UAGUACUUGGAUGGGGUCUA

AUACCACCCUGAACGCGCCC**C**AUCUCGUCUGAUCUCGGAAG

GGGACUCUUAAUCCCAGGGU**C**GUGGGUUUGAGCCCCAUGUU

CGCCUCACACGCGAAAGGUC**C**CCGGUUCGAUCCCGGGCGGA

UCGCCUCACACGCGAAAGGU**C**CCCGGUUCGAUCCCGGGCGG

AAUCCCAGCGGUGCCUCAAC**C**GAGCGUCCAAGCUCUUUCCA

AUUCUCAAACUUUAAAUGGG**C**AAGAAGCCCAGCUAGCUCAG

CAGUGCGAGCGGAGCAAUGC**C**GAGGUUGUGAGUUCGAUCCU

AAUCUGGCCUACGGCCAUAC**C**ACCCUGAACGUGCCCGAUCU

GGGCGUGGCAAUCCUUAGGU**C**GCUGGUUCGAUUCCGGCUCG

CAGUACAUGCAGAGCAAUGC**C**GAGGUUGUGAGUUCGAGCCU

GAAGCUAAGCAGGGUCGGGC**C**UGGUUAGUACUUGCAUGGGA

GGUCACGACCCUCAGGCUUU**C**AAUCUGAGGGUCCAGGGUUC

CAGGCUUUCAAUCUGAGGGU**C**CAGGGUUCAUAUCCCUGUUC

AGGCUUUCAAUCUGAGGGUC**C**AGGGUUCAUAUCCCUGUUCA

CGUCACCUUUCAGCAGUUCC**C**AUAUGGUCUAGCGGUUAGGA

CUCUUCGAAUGCACUUGCGG**C**CCGGGUUCCUCCCAGGGCUA

CGCUUCGCAUGUGUGAGGUC**C**CGGGUUCAAUCCCCGGCAUC

CGCCUGCCACGCGGGAGGCC**C**GGGUUCGAUUCCCGGCCCAU

UCGCCUGCCACGCGGGAGGC**C**CGGGUUCGAUUCCCGGCCCA

CUCGCCUGCCACGCGGGAGG**C**CCGGGUUCGAUUCCCGGCCC

GGAUCAGAAGAUUCUAGGUU**C**GACUCCUGGCUGGCUCGCGA

UGACUACGGAUCAGAAGAUU**C**UAGGUUCGACUCCUGGCUGG

UGACUGCAGAUCAAGAGGUC**C**CUGGUUCAAAUCCAGGUGCC

GAAGCUAAGCAGGGUCGGGC**C**UGGUUAGUACUUGGAUGGAG

UCGCCUAACACGCGAAAGGU**C**CCCGGUUCGAAACCGGGCGG

CGCCUAACACGCGAAAGGUC**C**CCGGUUCGAAACCGGGCGGA

GAAGCUAAGCAGGGUCGGGC**C**UGGUUAGUACUUGGAAGGGA

UUAGCACUUGCAUGGGAGAC**C**GCCUGGGAAUACCGGGUGCU

GUUAGCACUUGCAUGGGAGA**C**CGCCUGGGAAUACCGGGUGC

GCAGUGUCGGGCCUGGUUAG**C**ACUUGCAUGGGAGACCGCCU

AUCUCGUCUGAUCUCGGAAG**C**UAAGCAGUGUCGGGCCUGGU

CCAUACCACCCUGAACGCGC**C**CGAUCUCGUCUGAUCUCGGA

GUUAGUAUUUGGAUGGGAGA**C**CGCCUGGGAAUAGCAGGUGC

UUAGUAUUUGGAUGGGAGAC**C**GCCUGGGAAUAGCAGGUGCU

CCAUACCACCGUGAAUGCGC**C**CGAUCUCGUCUGAUCUCGGA

GUGGUCUAGUGGUUAGGAUU**C**AGUGCUCUCUCAUAAAAUAA

AGCACUCUGGACUCUGAAUC**C**AGCGAUCUGAGUCACGGCAC

AUCUCGCCUGCUCUCGGAAG**C**UAAGCAGGGUGGGGCCUGGU

AAGCGGUUACCUCCUCAUGC**C**GGACUUUCUAUCUGUCCAUC

AGUUCUUUAAUUGAAACAAG**C**AACCUGUCUGGGUUGUUCGA

AGCAACCUGUCUGGGUUGUU**C**GAGACCCGCGGGCGCUCUCC

AACCUCUGGGCUGGCUUUAG**C**UCAGCGGUUACUUCGCGUGU

CCUCUGGGCUGGCUUUAGCU**C**AGCGGUUACUUCGCGUGUCA

CUUUAGCUCAGCGGUUACUU**C**GCGUGUCAUCAAACCACCUC

CUCUCUGGGUUGUUCGAGAC**C**CGCGGGCGCUCUCCAGCCCU

AAUUACUGUCUACGGCCAUA**C**CACCCUGAACGCGCCUGAUC

ACUGUCUACGGCCAUACCAC**C**CUGAACGCGCCUGAUCUCGU

AGUGGUUAUCACGUUCGCCU**C**ACACGCGAAAGGUCCCCGGU

CUGGUCUUGUAAACCAGGGU**C**GCGAGUUCAAAUCUCGCUGG

GAUGUAGCUCAGUGGUAGAG**C**GCAUGCUUUGCAUGUAUGAG

AUGCUUUGCAUGUAUGAGGC**C**CCGGGUUCGAUCCCCGGCAU

GUUGAGAUACGGUCAUCGGG**C**GCCCGGCUAGCUCAGUCGGU

AGAAUACAGACAUGGCCGCC**C**GGCUAGCUCAGUCGGUAGAG

AGUUAGAAUACAGACAUGGC**C**GCCCGGCUAGCUCAGUCGGU

GUUAGUACUUGGAUGGGAGA**C**CGCCUGGGAAUACCGGGUGC

UUAGUACUUGGAUGGGAGAC**C**GCCUGGGAAUACCGGGUGCU

UUGGUGGCGGAAGGCGGCCC**C**AUGGUGUAAUGGUUAGCACU

AAGGCGAUGGACUUGAAAUC**C**AUUGGGGUCUCCCCGCGCAG

GACUACGGAUCAGAAGAUUC**C**AGGUUCGACUCCUGGCUGGC

UGUCUAGUAAACAGGAGAUC**C**UGGGUUCGAAUCCCAGCGGG

GAUGUAGCUCAGUGGUAGAG**C**GCAUGCUUCGCAUGUAUGAG

AUGCUUCGCAUGUAUGAGGU**C**CCGGGUUCGAUCCCCGGCAU

UGCUUCGCAUGUAUGAGGUC**C**CGGGUUCGAUCCCCGGCAUC

GAGCUACGUUCGGGUGAGCC**C**GGCUAGCUCAGUCGGUAGAG

GUCCUUAGACAUCCUUAGGU**C**GCUGGUUCGAAUCCGGCUCG

AAACUAAGGCAUCCUUAGGU**C**GCUGGUUCGAAUCCGGCUCG

CGAUAGCUCAGUUGGUAGAG**C**GGAGGACUGUAGGCUCAUUA

AGGACUGUAGGCUCAUUAAG**C**AAGGUAUCCUUAGGUCGCUG

UAAGCAAGGUAUCCUUAGGU**C**GCUGGUUCGAAUCCGGCUCG

GUAAUGACAUCCUUCGAUAG**C**UCAGCUGGUAGAGCGGAGGA

AAUGACAUCCUUCGAUAGCU**C**AGCUGGUAGAGCGGAGGACU

GAAUGUGGUCAUCCUUAGGU**C**GCUGGUUCGAAUCCGGCUCG

AGCAAGCCCUCUUAGCGCAG**C**UGGCAGCGCGUCAGUCUCAU

CAGUCUCAUAAUCUGAAGGU**C**CUGAGUUCAAGCCUCAGAGA

AGUCUCAUAAUCUGAAGGUC**C**UGAGUUCAAGCCUCAGAGAG

UAAUCUGAAGGUCCUGAGUU**C**AAGCCUCAGAGAGGGCAUCA

UCGCGGGUUCGAUCCCCGUA**C**UGGCCAGACGUGACUUUUUA

GGUGCUAAUAACGCUAAGGU**C**GCGGGUUCGAUCCCCGUACU

CGCUUCGGGUGUGAGAGGUC**C**CGGGUUCAAAUCCCGGACGA

GCUUCGGGUGUGAGAGGUCC**C**GGGUUCAAAUCCCGGACGAG

GGUGUGAGAGGUCCCGGGUU**C**AAAUCCCGGACGAGCCCACU

AAUCCAUUGUGCUCUGCACG**C**GUGGGUUCGAAUCCCACCCU

GGAUCAGAAGAUUCUAGGUU**C**GACUCCUGGCUGGCUCGGAG

AGAAGAUUCUAGGUUCGACU**C**CUGGCUGGCUCGGGUGUUAA

AUCCAAUGGACAGGUGUCCG**C**GUGGGUUCGAGCCCCACUCC

UUGCCUAACACGCGAAAGGU**C**CCCGGUUCGAAACCGGGCAG

UGCCUAACACGCGAAAGGUC**C**CCGGUUCGAAACCGGGCAGA

CGCCUCACACGCGAAAGGUC**C**CCGGUUCGAAACCGGGCAGA

CGCCUUACACGCGAAAGGUC**C**UCGGGUCGAAACCGAGCGGA

AGGAAAUGCUAGUUCAGCUU**C**AGGUUCCAUGGUGUAAUGGU

UGGACUCUGAAUCCGGUAAU**C**CGAGUUCAAAUCUCGGUGGA

GGACUCUGAAUCCGGUAAUC**C**GAGUUCAAAUCUCGGUGGAA

UGAAUCCGGUAAUCCGAGUU**C**AAAUCUCGGUGGAACCUUGG

AAUCCAUUGUGCUCUGCACG**C**GUGGGUUCGAAUCCCACCUU

AGACUUUUAAUCUGAGGGUC**C**AGGGUUCAAGUCCCUGUCCA

CAGACUUUUAAUCUGAGGGU**C**CAGGGUUCAAGUCCCUGUCC

AAUCCAUUGGGGUUUCCCCG**C**GCAGGUUCGAAUCCUGUCGG

CACGAGCUAUUGCGAGGUUC**C**AUGGUGUAAUGGUUAGCACU

UGAAUCCAGCGAUCCGAGUU**C**AAAUCUCGGUGGAACCUGUC

AAUCCAUUGGGGUUUCCCCA**C**GCAGGUUCGAAUCCUGCCGA

AAUCCAUUGGGGUUUCCCCG**C**GCAGGUUCGAAUCCUGCCGA

AAGGCGAUGGACUAGAAAUC**C**AUUGGGGUUUCCCCGCGCAG

UAAGGCGAUGGACUAGAAAU**C**CAUUGGGGUUUCCCCGCGCA

GGACUCUGAAUCCAGCGAUC**C**GAGUUCAAGUCUCGGUGGAA

UGGACUCUGAAUCCAGCGAU**C**CGAGUUCAAGUCUCGGUGGA

AACCCAUUGGGGUCUCCCCG**C**GCAGGUUCGAAUCCUGCCGA

CCGUCUGUCACGCGGGAGAC**C**GGGGUUCGAUUCCCCGACGG

CCCGUCUGUCACGCGGGAGA**C**CGGGGUUCGAUUCCCCGACG

UUCUGGUCUCCGCAUGGAGG**C**GUGGGUUCGAAUCCCACUUC

CAGACUCAAGUUGCUACUUC**C**CAGGUUUGGGGCUUCUGGUC

GUGGUCUAAGGCGCCAGACU**C**AAGUUGCUACUUCCCAGGUU

GGCCGAGUGGUCUAAGGCGC**C**AGACUCAAGUUGCUACUUCC

UUCUGGUCUCCGUAUGGAGG**C**GUGGGUUCGAAUCCCACUUC

ACUGCUUCCUGUGUUCGGGU**C**UUCUGGUCUCCGUAUGGAGG

GUGGUCUAAGGCGCCAGACU**C**AAGCUUACUGCUUCCUGUGU

GGUCUCGUAAACCUAGGGGU**C**GUGAGUUCAAAUCUCACCAG

AAAGGUCCCUGGAUCAAAAC**C**AGGCGGAAACAAGUGGUUAC

CGCCUAACACGCGAAAGGUC**C**CUGGAUCAAAACCAGGCGGA

UCGCCUAACACGCGAAAGGU**C**CCUGGAUCAAAACCAGGCGG

CCCACCAGCAGGGUUGUUUC**C**GUAGUGUAGUGGUUAUCACG

UCAAUUCUCACUCCCACCAG**C**AGGGUUGUUUCCGUAGUGUA

GUUAGCUCAGUCGGCUAGAG**C**GUGGUGCUAAUAACGCCAAG

AAUCCAAUGGGGGUUCCCCG**C**GCAGGUUCAAAUCCUGCUCA

AAGGUGUUGGACUCGAAAUC**C**AAUGGGGGUUCCCCGCGCAG

CGCCUAACACGCGAAAGGUC**C**GCGGUUCGAAACCGGGCGGA

UCGCCUAACACGCGAAAGGU**C**CGCGGUUCGAAACCGGGCGG

AUCCUGGGUUCGAAUCCCAG**C**GGGGCCUUGAUUUCUGUACC

UGGCUUAGCUGGUUAAAGCG**C**CUGUCUAGUAAACAGGAGAU

CGUGGCUUAGCUGGUUAAAG**C**GCCUGUCUAGUAAACAGGAG

GGUGCUAAUAACGCCAAGGU**C**GCGGGUUCGAUCCCCGUACU

AUCCAAUGGGCUGGUGCCCG**C**GUGGGUUCGAACCCCACUCU

GGAUGGCCGAGUGGUUAAGG**C**GUUGGACUUAAGAUCCAAUG

UGUCUAGUAAACAGGAGAUC**C**UGGGUUCGAAUCCCAGCGAG

AAAAGAAGAAGCUUUGUAAC**C**GUUGGUUUCCGUAGUGUAGU

UGGGCCCAUAACCCAGAGGU**C**GAUGGAUCUAAACCAUCCUC

GGACUCUGAAUCCAGCGAUC**C**GAGUUCAAAUCUCGGUGGGA

UGGACUCUGAAUCCAGCGAU**C**CGAGUUCAAAUCUCGGUGGG

AAUCGCUAAGAACUUGGCCC**C**AUGGUGUAAUGGUCAGCACU

AAAUCGCUAAGAACUUGGCC**C**CAUGGUGUAAUGGUCAGCAC

CCCCAUGGUGUAAUGGUUAG**C**ACUCUGGACUUUGAAUCCAG

AGGUGGCCGAGUGGUUAAGG**C**GAUGGACUGCUAAUCCAUUG

AAUCCAUUGUGCUCUGCACA**C**GUGGGUUCGAAUCCCAUCCU

UGUCUUGUAAACAGGAGAUC**C**UGGGUUCGAAUCCCAGUAGA

GGCUCCAGUCUCUUCGGGGG**C**GUGGGUUUGAAUCCCACCGC

UGUCUCGUAAACAGGAGAUC**C**UGGGUUCGACUCCCAGUGGG

UGGCUUAGUUGGUUAAAGCG**C**CUGUCUCGUAAACAGGAGAU

AGUGCACCUGUGAGCAAUGC**C**GAGGUUGUGAGUUCAAGCCU

UGGACUUUGAAUCCAGCAAU**C**CGAGUUCGAAUCUCGGUGGG

GGACUUUGAAUCCAGCAAUC**C**GAGUUCGAAUCUCGGUGGGA

UGCUUAGCAUGUACGAGGUC**C**CGGGUUCAAUCCCCGGCACC

UGCUUUGCAUGUAUGAGGUC**C**CGGGUUCGAUCCCCGGCAUC

UGUCUCGUAAACAGGAGAUC**C**UGGGUUCGAAUCCCAGCGGG

CUGUCUCGUAAACAGGAGAU**C**CUGGGUUCGAAUCCCAGCGG

GCUUAGCAUGCAUGAGGUCC**C**GGGUUCGAUCCCCAGCAUCU

UGCUUAGCAUGCAUGAGGUC**C**CGGGUUCGAUCCCCAGCAUC

GAUGUAGCUCAGUGGUAGAG**C**GCAUGCUUAGCAUGCAUGAG

UGCUUCGCAUGUAUGAGGCC**C**CGGGUUCGAUCCCCGGCAUC

AUGCUUCGCAUGUAUGAGGC**C**CCGGGUUCGAUCCCCGGCAU

UGCUUCGCAUGUACGAGGUC**C**CUGGUUCAAUCCCUGGUACC

GUGCUUAGCAUGCACGAGGC**C**CUGGGUUCAAUCCCCAGCAC

UGCUUAGCAUGCACGAGGCC**C**UGGGUUCAAUCCCCAGCACC

UGCUUAGCAUGCACGAGGCC**C**CGGGUUCAAUCCCUGGCACC

UGUCUAGUAAACAGGAGAUC**C**UGGGUUCGACUCCCAGCGGG

CCCAGCGGGGCCUUGGUUGG**C**AAGGUCAGUGUGCCUUUCUG

AAGCCAACAGAACUCUCAUG**C**GGUCAGGGGGUGUAGCUCAG

GUGCUUCGCAUGUACGAGGC**C**CCGGGUUCGACCCCCGGCUC

UGCUUCGCAUGUACGAGGCC**C**CGGGUUCGACCCCCGGCUCC

CAUGUACGAGGCCCCGGGUU**C**GACCCCCGGCUCCUCCAGUU

UGCUUAACAUUCAUGAGGCU**C**UGGGUUCGAUCCCCAGCACU

AGACUUUUAAUCUGACGGUG**C**AGGGUUCAAGUCCCUGUUCA

UGAGGCCCCGGGUUCGAUCC**C**CGGCACCUCCAAAUGGUGGU

GUGAGGCCCCGGGUUCGAUC**C**CCGGCACCUCCAAAUGGUGG

UGCUUUGCAUGUGUGAGGCC**C**CGGGUUCGAUCCCCGGCACC

AUGCUUUGCAUGUGUGAGGC**C**CCGGGUUCGAUCCCCGGCAC

CCGGGUUCGAUCCCCGGCAC**C**UCCAAACGGUGACUUUUUGC

AUGAGGUCCCGGGUUCGAUC**C**CCGGCACCUCCAAACGGUGA

GCUUUGCAUGUAUGAGGUCC**C**GGGUUCGAUCCCCGGCACCU

UGCUUUGCAUGUAUGAGGUC**C**CGGGUUCGAUCCCCGGCACC

AUGCUUUGCAUGUAUGAGGU**C**CCGGGUUCGAUCCCCGGCAC

GGUGUAGCUCAGUGGUAGAG**C**GCAUGCUUUGCAUGUAUGAG

UGCUUAGCAUGCACGAGGUC**C**UGGGUUCGAUCCCCAGUACC

GUGCUUAGCAUGCACGAGGU**C**CUGGGUUCGAUCCCCAGUAC

ACCUCCAAGUGAUGGUUUCC**C**UCUGGCAGUUCUCAAGCGAC

CACCUCCAAGUGAUGGUUUC**C**CUCUGGCAGUUCUCAAGCGA

AUGCUUUGCAUGUAUGAGGC**C**UCGGUUCGAUCCCCGACACC

AGAUAGCUCAGUUGGGAGAG**C**GUUAGACUGAAGAUCUAAAG

UGCUUAGCAUGCACGAGGUC**C**UGGGUUCAAUCCCCAAUACC

CGAUCCCCGACACCUCCAAG**C**GAUGGUUUUGCUCUGGUAGU

AUGCUUUGCAUGUAUGAGGC**C**UCGGGUUCGAUCCCCGACAC

GACCGAAGAUCUUAAAGGUC**C**CUGGUUCAAUCCCGGGUUUC

AGACCGAAGAUCUUAAAGGU**C**CCUGGUUCAAUCCCGGGUUU

CAGUUGGGAGAGCGUUAGAC**C**GAAGAUCUUAAAGGUCCCUG

CAAUCCCCGGCACCUCCAUU**C**CUUUUGCUUUUAAUUUUUUU

UGCUUAGCAUGCACGAGGCC**C**CGGGUUCAAUCCCCGGCACC

GUGCUUAGCAUGCACGAGGC**C**CCGGGUUCAAUCCCCGGCAC

GCUUAGCAUGCACGAGGCCC**C**GGGUUCAAUCCCCGGCACCU

GGUGUAGCUCAGUGGUAGAG**C**GCGUGCUUAGCAUGCACGAG

AUUCUGGUCUCCAAUGGAGG**C**GUGGGUUCGAAUCCCACUUC

GUGGUCUAAGGCGCCAGACU**C**AAGCUAAGCUUCCUCCGCGG

GGCCGAGUGGUCUAAGGCGC**C**AGACUCAAGCUAAGCUUCCU

UGGCCGAGUGGUCUAAGGCG**C**CAGACUCAAGCUAAGCUUCC

UGUCCCAGGUGUCAGGAUGG**C**CGAGUGGUCUAAGGCGCCAG

UGGCCGAGUGGUCUAAGGCG**C**CAGACUCAAGCUUGGCUUCC

GGCCGAGUGGUCUAAGGCGC**C**AGACUCAAGCUUGGCUUCCU

GUGGUCUAAGGCGCCAGACU**C**AAGCUUGGCUUCCUCGUGUU

UCUAAGGCGCCAGACUCAAG**C**UUGGCUUCCUCGUGUUGAGG

CCAGACUCAAGCUUGGCUUC**C**UCGUGUUGAGGAUUCUGGUC

UUUUUUGUUAAGUAGGGUUC**C**AUGGUGUAAUGGUUAGCACU

CAGUCUCAUAAUCUGAAGGU**C**CUGAGUUCGAACCUCAGAGG

AGUCUCAUAAUCUGAAGGUC**C**UGAGUUCGAACCUCAGAGGG

UGAAGGUCCUGAGUUCGAAC**C**UCAGAGGGGGCAAGGCGUCU

GGCUCCAGUCUCUUCGGGGG**C**GUGGGUUCAAAUCCCACCGC

AGUUUCAUAAUCUGAAAGUC**C**UGAGUUCAAGCCUCAGAGAG

CAGUUUCAUAAUCUGAAAGU**C**CUGAGUUCAAGCCUCAGAGA

AUCCAAUGGACAUAUGUCCG**C**GUGGGUUCGAACCCCACUCC

UGGGCUUUGAAUCCAGCAAU**C**CGAGUUCGAAUCUUGGUGGG

GGGCUUUGAAUCCAGCAAUC**C**GAGUUCGAAUCUUGGUGGGA

UCUCUGGAAGGUAAGCAGGG**C**CUGGUUAGUACUUGGAUGGG

GAACGCGCCCGAUCUGGUCU**C**AUCUCGGAAGCUAAGCAGGG

ACCAGGCGCCACUGCCAUAC**C**ACCCUGAACGCGCCCGAUCU

CAAGAGAUAACUACCAGUGG**C**UCGUUGGUCUAGGGGUAUGA

AGAGAUAACUACCAGUGGCU**C**GUUGGUCUAGGGGUAUGAUU

GUCAGUUACAGAUCGAACUC**C**UUGUUCUACUCUUUCCCCCC

GGCAUAGCUCAGUGGUAGAG**C**AUUUGACUGCAGAUCAAGAG

AUUACACUGUCCUCCUAUGA**C**UGGGUGUAUGGCUCAGGGGU

UGACUGCAGAUCAAAAGGUC**C**CUGGUUCAAAUCCAGGUGCC

GAAUGCACCUGAUCUCGGAA**C**CUAAGCAGGGUCGGGGCUGG

AUGUCUUCUGAUCUCGGAAG**C**UAAGCAGGGUCGGGCCUGGU

GUUUGUGUCUACGGCCAUAC**C**ACCCUGAAUGCGCCCGAUCU

CCAUACCACCCUGAAUGCGC**C**CGAUCUCGUCUGAUCUCAGA

AGGGGACGCCGACACACGUA**C**ACGUCCCUUCGAUAGCUCAG

CGUACACGUCCCUUCGAUAG**C**UCAGCUGGUAGAGCGGAGGA

AGCAGGAGACAUCCUUAGGU**C**GCUGGUUCGAUUCCGGCUCG

GUGCCCGCUCCCUUCGAUAG**C**UCAGCUGGUAGAGCGGAGGA

GCCCGCUCCCUUCGAUAGCU**C**AGCUGGUAGAGCGGAGGACU

UUAGGUCGCUGGUUCGAUUC**C**GGCUCGAAGGAGAGACACCC

CAGUCUCAUAAUCUGAAGGU**C**GUGAGUUCGAUCCUCACACG

UAUGUAUACAUGUGCCAUGC**C**CGAUCUCGUCUGAUCUCGGA

AAAACUUAAAACUUUACAGU**C**AGAGGUUCAAUUCCUCUUCU

AGAACUGCUAACUCAUGCCC**C**CAUGUCUAACAACAUGGCUU

UGGCCUUUCACACUGGCAGC**C**UGGGUUCAAUUCCCAGCUUA

GUUAGUACUUGGAUGGGAGA**C**CACCUGGGAAUACCGGGUGC

UUAGUACUUGGAUGGGAGAC**C**ACCUGGGAAUACCGGGUGCU

# (2) List of the 1425 RNA samples in the negative subset $\mathbb{S}^{\mathbf{-}}$

GCGCUGCGUGGCUUGGAAGG**C**ACUGGGGGGCGGGGGUGGAG

CGAGGCAUGACUGAGCUGGC**C**UCAGCCCCCAGGUCCUUCCU

GGUGGUUAUUUAGUUGAGGG**C**CAGCAAACAGGUGUCACCUC

CGUCCCUUGCCAAACCUGUG**C**UGUGGGCCAGGCCUGAGCUG

CGAGGACACCCCUCUUGGCU**C**UGGCUGUGGAAUGUGGAGCU

GAGUAGGGAUGGGGCUCUGC**C**AGGAUGUCUCACGUCAGGGG

CUAUAGUGAGAGUGAGCACC**C**UUCCCGUGCCCCUCCCGAGG

UGUGCUGGGCCUGAGACAGU**C**ACAGCUUCCCCUGGGCUCCU

ACCGCGUCACUCCCCGGGCG**C**CGAGUUCCGCCGGCCUGGCA

CGACAGCAGUGUCUCCUUUA**C**CCUCCUGAAGGAGUGUGAGG

GGCCCAGAGAGGGGCAGGGC**C**CGCCCAAGGCUGCACAGUGA

GCCCCUCCUCCUGGUCACAC**C**CCUGCCCAGGAGGGGAGCCU

GUUGGGGCCUCUCUGGGGUG**C**ACAUCAGCCCCUCCAUCCUC

GGAGUGUGAGGUGGCACUCU**C**AUCCCCACCCAACAGGAGGG

UGAGGGACGGCCCUUGUCUG**C**GGUCAUCCCUCCCCUCCUGA

GCUGGCAGCAUUUCCAUCUU**C**UCUGUGGUCAGUUCAGGGUG

GGUGCGCGGGGUGCAGGGGA**C**GCCGCUUGAGGACUCCCAGC

CCAGGAUGAGCAAGGCUGGC**C**CAGCAUCUCUGAGUGAGUGG

CGGGGAAGCGUUCGCAGAAA**C**UACAGGGUGGUUAUUUAGUU

UUGCCAGUUGUCCUCCCCAG**C**AGUUGUGCGCGAGGUCCAUC

CUGGGGAGGGGCGACACACA**C**UGGUGGUGAGUUCAGGGCAA

CCCACGCCCCUACCCGGGGG**C**UCCCAUCCUGGGGCUGGGAC

UCCCUCUUGCCAGUUGUCCU**C**CCCAGCAGUUGUGCGCGAGG

CAGCCCCAGCCUCUGGGGCC**C**CAGGCCUGGGCAGGGGCCUG

CAGGCCCAGAUCCGGGCCCC**C**UGCGCUGCAAGGCCACCUCA

GGCGCCCGUCCGUCCCUUGC**C**AAACCUGUGCUGUGGGCCAG

AGGAGGUGUCUCAGGGGCUC**C**UGGCUUUGUCCUGGGAGUAG

CCUGAAGGAGUGUGAGGUGG**C**ACUCUCAUCCCCACCCAACA

GGACGGCCCUUGUCUGCGGU**C**AUCCCUCCCCUCCUGAGUGG

GCACCGCGUCACUCCCCGGG**C**GCCGAGUUCCGCCGGCCUGG

CAGGAUGGAUGUGAGAAAAA**C**AAUAACGCCCCCAAACCACA

CUGAGACAGUCACAGCUUCC**C**CUGGGCUCCUCUUCUCUCAU

UGUGGCACCAACUAGGGCUA**C**AGAAGAUGGAAUUUGACCUC

GCAGCCGCCGUGCUGGGCAC**C**CACCUUGGGAAAAGGUCGCU

GCACCCAGGUGCGCGGGGUG**C**AGGGGACGCCGCUUGAGGAC

ACAGCCCCUACUCUUGCUGC**C**CUGGUAGAAGACGAGGCCCA

GGCCCCGGCACCCACUGUCC**C**CUUGUGGCCCCUCCUCCUGG

UUUCUGCCCAUCCCUUGAGC**C**UCAGAACGUCCUGGGGUGAA

GAGUAUGAUCUGGAAGCCCC**C**AGGGCCGGCAAAGGGGUGCA

CCACUCAGGUGCUGUGGGUC**C**UUGGUCCCUCCCGGCCCCGC

CCCAGCGAGGCUCUGAGAAG**C**CGGGACACUCUGCCCAGGGA

GGCAGCUGAAGACUCUGCAU**C**CCUGUGAGACGGUCCCUGCC

CUGUGCACAUUCUUCAGGAC**C**ACAGAGGGGGAGCAGCUGCU

ACUCCCCGGGCGCCGAGUUC**C**GCCGGCCUGGCACGGGCUCA

GGUCCCGGGCUGGGCUUCCC**C**GUCCCACCAUCCAGCUGCCC

UCGAUUUCUGUCCUCAUCUG**C**GGGGCCUGGGAAGGAGCUGG

UCCCCACUGGGGAACCCCAA**C**CCGGACCAGGAGAUGGGCCC

AAUACUCAAAUAAGGAAUCA**C**AUAACAACAUAAGCAAAGAU

CACAUCAGCCCCUCCAUCCU**C**UUCACACUUCCCUCUUGCCA

UAAUCGGGCUUCAACCACAC**C**GCUGGGGAACAAAAAUAAAC

CUGCCCCCAACACCUACAGC**C**CCUGCCCUCCUUCUGAGGCU

GUCCCGACACCAUGGAUGGG**C**AGGGAGGCCAUCCUUACCAA

GGGGCCCCUUCCUCUGCCGC**C**CUGGACCUGGGCUGUGGCAC

GGUCCCUGCCCAGGCAGCAC**C**CAGGGGUGCAGUGCACAGUC

CUGCGUCUGCCAGGCAUGCC**C**UGCAUUCAGGCAGAACUCGA

ACCAUGGAUGGGCAGGGAGG**C**CAUCCUUACCAAAGCAAGCA

GGAGGGGCUCCCUGGGAUGA**C**ACCGUGGAAGAGGAGAUGCC

GACCCAGAGGACCAGAUGUG**C**CCCACGCCCCUACCCGGGGG

GUGAGGGACACAGCUGGGCU**C**UGGAGGCCUGGGAGGCCUGG

GCUGUGGCAGGGAGUAUUUU**C**UCUAAAAACAGGAUGGAUGU

GGCCUCUCUGGGGUGCACAU**C**AGCCCCUCCAUCCUCUUCAC

UGGGACAGAAGAGGCAGUCA**C**AGCCGUGUGGCCAAUCGAUU

GCAGGGGUGGGUCUAUGGGG**C**CCGUGUGCAUAGGGUUGUGU

UGCUCCUCGCCGUCAUAAAC**C**CGCGUGCACCGCGUCACUCC

GAUUUUUUCAUAAACCCAAA**C**ACAAAACACAAUAAAGUUCU

AUAAACCCAAACACAAAACA**C**AAUAAAGUUCUGUGGGAUGU

CCAGGCUUCCUGACUGCCCG**C**UCCCUCUUCACGUUGCUUCU

CAUCUUCUCUGUGGUCAGUU**C**AGGGUGAGGGAGCCUCCCGC

GGGCCACCUGGGUCCCGGGC**C**UCGGGGGUCCCUGGGACAGC

GGCUCUGGAGGCCUGGGAGG**C**CUGGGGAGGAGUCCUUGAGA

GUGGCCCCUCCUCCUGGUCA**C**ACCCCUGCCCAGGAGGGGAG

CUCUCAUUUCUUUCCUCUUC**C**UGCCCCGCCCCUCAGCAGAA

GGGCACUAUGACAGGGGGGA**C**AUUGGCAUGAGUUGUGGGAG

UCUGGGGUGCACAUCAGCCC**C**UCCAUCCUCUUCACACUUCC

CCCCGCUGGGAUGUGCUGGG**C**CUGAGACAGUCACAGCUUCC

GCUGGGCAACCUGGGACUGG**C**CCCUUAGCCCCUCUGGGUCU

AAUGGAGAUGACACACUUUG**C**ACUGAACAUGUACUGUGUAA

CAUUUCUCCAGACUCCCGGC**C**UUCCUAAUUUAUGUGCUGCU

GGAACCACGUUGGGGGCCAC**C**ACAGGGCAGAUGGGUCCCGG

GGGCAGGAUCUGGGGAGGGG**C**GACACACACUGGUGGUGAGU

UUACUUCCCCUGGGGUUUUA**C**AGACCACCGAAGCCCUGCGG

UGGGGUGGCUGGAUUUGGGG**C**ACGGAUGCCCAGGUGGGCUU

GAACCGGUGAUAUCAGAGUU**C**UUGGUGGGGAAAAUCUGCCC

UGGGCAACCUGGGACUGGCC**C**CUUAGCCCCUCUGGGUCUUG

AUUUCUUUCCUCUUCCUGCC**C**CGCCCCUCAGCAGAAUCUCU

CCCGUCCGUCCCUUGCCAAA**C**CUGUGCUGUGGGCCAGGCCU

GGACGCCGCUUGAGGACUCC**C**AGCAGCGUGGGACGUGAGCA

AGCCAUGCUGUGCUGGGCAA**C**CUGGGACUGGCCCCUUAGCC

AAAUCUGCCCAGGGUAGUAG**C**UCCAGAGAGCAGAGUCACAG

AGGAUGUCUCACGUCAGGGG**C**UGCUCGGGGCCUGCGGGACA

GCCCUUGUCUGCGGUCAUCC**C**UCCCCUCCUGAGUGGAAGGU

AGGAGAUGGGCCCCGGCACC**C**ACUGUCCCCUUGUGGCCCCU

GUGGAAGGUGAUGCCGUGGG**C**CCAGGGCUGGCUGCGUCUGC

CCGAGGCAUGACUGAGCUGG**C**CUCAGCCCCCAGGUCCUUCC

UGGGACCCCGGGACCGGGUG**C**UCGCUGAGGGACGGCCCUUG

UCUCUCAUUUCUUUCCUCUU**C**CUGCCCCGCCCCUCAGCAGA

CGGGGACCUCCGGCGGGUCC**C**GGGCUGGGCUUCCCCGUCCC

AUUUGACCUCUCCCCCUGGG**C**CACCCACCCAGCCCCAGCCU

GCCCAAGGCCCUGCCUCCCG**C**UGUCUCUCCCAUGGCCUCUC

GUCACAGCUUCCCCUGGGCU**C**CUCUUCUCUCAUUUCUUUCC

GCAGUCCUCCCCGGUGCCGG**C**CCAUCGGGGUGCGUUAGGUA

UCCCCACCCAACAGGAGGGG**C**UCCCUGGGAUGACACCGUGG

GGGCUCCUCUUCUCUCAUUU**C**UUUCCUCUUCCUGCCCCGCC

CGGCAAAGGGGUGCAGGGUG**C**UGGGGCUGAGGUAGUCCGGG

UGUUUUGGCCCAAGGCCCUG**C**CUCCCGCUGUCUCUCCCAUG

UCUCCCCCUGGGCCACCCAC**C**CAGCCCCAGCCUCUGGGGCC

GGUUCCGAGGCAUGACUGAG**C**UGGCCUCAGCCCCCAGGUCC

UCUCAGCCUCACGUGAGCAU**C**CACGGACACGUGGGGCCCCA

AAACAAUAACGCCCCCAAAC**C**ACACUUAAUGGGUGGGUCGC

GGUGGGCUUCUCCCUGUAGG**C**UCGGAAUGGGGACUGCAGAG

GAUGAGCAAGGCUGGCCCAG**C**AUCUCUGAGUGAGUGGGGAA

AGCUCGAGCGGGUAGGCGCC**C**GUCCGUCCCUUGCCAAACCU

CAGCCAGGUGCCAUGCAGGC**C**UGGACAGCUCCCCACUGGGG

CCCUGCAUUCAGGCAGAACU**C**GAGGGAGGACUCAUCUCCAA

CCUCCAUCCUCUUCACACUU**C**CCUCUUGCCAGUUGUCCUCC

CCGUGCCCCUCCCGAGGACA**C**CCCUCUUGGCUCUGGCUGUG

CAGAAGAUGGAAUUUGACCU**C**UCCCCCUGGGCCACCCACCC

AGUUCAGGGUGAGGGAGCCU**C**CCGCCCCUGUGGUUCCGAGG

GGAAGGAUGCUCCUCGCCGU**C**AUAAACCCGCGUGCACCGCG

UGAAAUUAAAUCGAAAUACU**C**AAAUAAGGAAUCACAUAACA

UCGGGGCCUGCGGGACACCU**C**CUUCCUUACUUCCCCUGGGG

UGGCCUGGGUCCCAGGAAUU**C**CGGGAAAUCUUUCCUAGCUA

UCCAAAUCACUACGCUACUU**C**CUGAAAACCUGUACUUGAGG

CUGACAUGGGCUCGUCCUGC**C**AUGGCCCAAUAAAUCUCGUG

AAGUUGGCGGGUAGCACUGC**C**CUGCACUAGACACACAUUUA

GGACAGUUCUCCACCAUGGU**C**AGGUCUGAGCGAGGGCAGCU

CUCACCCGGAGUCCGUGGUU**C**ACCUCGGCUCACUCUUGGCA

GUGGCCACGUUGUCCAUGUG**C**CCUGUACCUCGUGCUGCUGG

AACAGUGGGACCUGCACAUC**C**GAGAGUCCGAGCUUUACUGU

GCCUCGGACCUCAGGGGCUU**C**CUGGUCGCAGCAGGCGGCCC

ACUUAGGCAGAGAAGAGGCC**C**UGUGUCCCGGGUGCCUGGUG

CACCAAAAUGUCUUCCAACG**C**AGCUGCGUUGUUCACCCCCC

CCCAGUCUGGGUAACAAGAA**C**AAACAUUAAAAAUCCCCGGC

GGUGCCUACCCAGCAGGGGC**C**CUGCCUGUAGGAGCAUCACC

UUUGGGCAGGUGUUUGAUGA**C**GACACACCCCCCCAUUACAG

UUGGGGCAUCUUGUGCACCU**C**UUUGCUCUCUGCUCUUUGAC

AGGGAAUCUGUGAACCUGGG**C**AGCCCCGGGGGCUGGGCUCA

UCCAGAUGGGAAAACCAAGA**C**UCAGGUGGUUGAGUGACGUG

CCACAGAGGAGUUUCUCUGU**C**CUAAAAAUCCCCUCUGCUCC

AAGCCAAGCUAGAGAGGCCU**C**AGUUUCCCCUCCCGUCCUCC

ACACAACACGGAGCGUUUUG**C**UUGUAACUCUGUGGUUGGGG

GUUCCCCACCAUGGUCAAGU**C**UGAGUGAGGAGGGCAGCUCC

UCUCUCCUUUUAUCCUGCUG**C**AUGCGGCAGAGGGACAAAUG

CUGCCGAGCUGGACACUGUC**C**AGCCUGAGCGUGGCCACGUC

GCAGGGAGGUCUGAGGCUUC**C**CCGGUGGGGACUAAGGCUGU

ACACCCCCCCAUUACAGCAG**C**CCACAGAGGAGUUUCUCUGU

GGACAGUUCCCCACCAUGGU**C**AAGUCUGAGUGAGGAGGGCA

AGGUCUGAGCGAGGACAGCU**C**CCCACCACGGUCAGGUCUGA

CCCAAUUUCUUCUUAAGUCA**C**CGUCAGAGCCCACGAUACCC

GUUCCCAUAUGGCCCCUGCC**C**CACCCACGCCCUCCCUCGUG

AGUGUUCGCUGAAGCUGUGU**C**UAAACCUCUGACUCAGUAAG

CACCAGGUCUCACUGGAGGA**C**GGGCCGGCUUCUCCCAUGCC

CUCUGUCCUAAAAAUCCCCU**C**UGCUCCGCCCACUCCUCCCU

AUUUGUAGACUCGGCGAGUC**C**UUUCUUGCCUCCUUUCCUGA

GUUCUGGCCUGGCCUUUGAC**C**UCUGUGGGACCCAGUCCUUG

GUGUCCAGGCUGUGCAGUUG**C**UGGGGCUAGGUCCGCUCGCU

GCAGUUGCUGGGGCUAGGUC**C**GCUCGCUGGCUUGUGGGGCA

UAUGUCUCCACGCACCAGGG**C**GCCUGCCUUUAUUCACUCUC

AUCUGUGAACCUGGGCAGCC**C**CGGGGGCUGGGCUCAGGGCA

UGCCAGGCUGAACCCCCUCC**C**CAAAGCUGCGUCAGAGGACA

UAGAAAGGUGGUUUGAAAGG**C**AUGCUGUGCAUUCACAGUUU

GUCCAGCCUGAGCGUGGCCA**C**GUCGUGGUGGUGUCCAGGCU

ACAGAGGGAGGGAAGGGCUG**C**ACAGCAGGGGCUCAGCGCAU

UUCCUUUCUUAUGGGAUCAU**C**AGAGACUUUUAUGCUCGUCU

CUGGAGAUACAGCCAGACAC**C**UCCACACAGUGGCUGAUUCC

AGUUUCUCUGUCCUAAAAAU**C**CCCUCUGCUCCGCCCACUCC

AAAUGACUUUCCUGACCACA**C**AUCUUUGUUAAAACAGCACU

AGGGCGCCUGCCUUUAUUCA**C**UCUCUCAGUUUAAAAUACAG

AGACCUCGUGGGGGCUUUUU**C**UCCAGCCCAUCCUCUCCCUG

AGUUAUUGUGAAAUGACUUU**C**CUGACCACACAUCUUUGUUA

ACGGGCCGGCUUCUCCCAUG**C**CCUGGGUCCUCCGCACACUC

GUCUGAGCGAGGACAGUUCC**C**CACAACGGUCAGGUCUGAGU

GUCUCCACGCACCAGGGCGC**C**UGCCUUUAUUCACUCUCUCA

AGAACAAACAUUAAAAAUCC**C**CGGCUGAAGUUGGCGGGUAG

AGAAUCCUGACUGUCGAAGC**C**CCCAAAUAGUUCUGUAGGAU

UUCCACAACAAGAUGAGGUG**C**AGCCACAAUGCGUGUCUUGU

GGCGCCUGCCUUUAUUCACU**C**UCUCAGUUUAAAAUACAGAG

CAUGUGGGUCCGGGAUAUGC**C**UGGGGUCAUGGGCCCCUCCC

AUUGCCUCGGACCUCAGGGG**C**UUCCUGGUCGCAGCAGGCGG

GUCUUCCUUCCAAUUCGAUA**C**UCUCCUGAGAUUCAUCUGUG

UGCACACAACCCAGUGUUCG**C**UGAAGCUGUGUCUAAACCUC

GCGAGGGCAGCUCCCCACCA**C**GGUCAGGUCUGAGCGAGGAC

CUUCAGGUUCCGUCUGAUCC**C**AGGUGGGUGGGUGGCUCUGA

GUCCUCUCAGCCCUUAAGGU**C**AACUGUAACAUUUUUAGAAU

ACCUCUCCACGUAGUCAACA**C**AUAAAAAUUAAGCUCUGCCA

CGUGGUCUGUGGGGAGAAUG**C**UCUGGGCCUGACUUGAGUGU

CAGUCAGGUAUGAGCGAGGA**C**AGUUCCCCACCACGGUCAGG

AGAUCAGAGUACAUUAAACA**C**ACAUUUAGCUUUGGAAAUGU

CUCUCCCUGAGCUUGUUUAU**C**AAGAAGAUGCUUCUAGAGUG

UGAGUGAGGACAGUUCCCCA**C**CAUGGUCAAGUCUGAGUGAG

CGAGCUGGACACUGUCCAGC**C**UGAGCGUGGCCACGUCGUGG

AUUUUUCAUAUGACUAGCAU**C**AGGAAAAAAGUCAUCACAUU

GGUAUGAGCGAGGACAGUUC**C**CCACCACGGUCAGGUCUGAG

CCAGUUCCUGGGCUUCCUCC**C**UGUUCUUCAAGGAGAGACUG

UGCAGUUGCUGGGGCUAGGU**C**CGCUCGCUGGCUUGUGGGGC

AACCUCUGACUCAGUAAGGU**C**UUUUUAAAGAUACAGCCAUU

CACAAACAGUUUUAGAAAUG**C**UCUUGCUUCUGCCAUUUAUA

CUGAGAAGGUUUUGGGGCAU**C**UUGUGCACCUCUUUGCUCUC

AGGGUGGGUGCAUGGAAGAG**C**AUUGCGGUAUCAUGAGAGGU

GGCUUUUUCUCCAGCCCAUC**C**UCUCCCUGAGCUUGUUUAUC

AAAAUACAGAGUUAUUCACC**C**CUGAGCUUCUGUGCACACAA

CUGUAAAAUGUGGCUCUGUG**C**CUUAAGAAUGACAUUGUUAA

GUCCUUUCUUGCCUCCUUUC**C**UGAGUUCUGUGUUGUCUUAA

CAAGCGUGGUCCGUUUCACC**C**CCCUCCACCCUGACAUGCAC

CCUCGGACCUCAGGGGCUUC**C**UGGUCGCAGCAGGCGGCCCA

GAUGCUGUGAGGGCCUGUGG**C**UGCCUCACGUCUGCGGAAGA

UAAGUGAGUAUUUCCUCUCU**C**CUUUUAUCCUGCUGCAUGCG

UUUAAGUGAGUAUUUCCUCU**C**UCCUUUUAUCCUGCUGCAUG

GUGGACAGGUCGCGAGGACA**C**CAGGUCUCACUGGAGGACGG

UGGCCACGUUGUCCAUGUGC**C**CUGUACCUCGUGCUGCUGGC

GAUUUUAUUCAAGAUUAUUU**C**AAGAUAACAACUGGCAGUAA

CAACGCAGCUGCGUUGUUCA**C**CCCCCACAGUGGAUAAGAGU

AUCUUGUGCACCUCUUUGCU**C**UCUGCUCUUUGACUUCAGCU

GAGGCUUCCUGGGAGGGGGC**C**ACGCUGAGGAAGCCCGUGUG

CAAACAGUUUUAGAAAUGCU**C**UUGCUUCUGCCAUUUAUAAC

GCUUUCGAGUUGUCAUUCUC**C**CGCGUGGUCUGUGGGGAGAA

CCAGGGCGCCUGCCUUUAUU**C**ACUCUCUCAGUUUAAAAUAC

UGCUGGAAGAGUGAUACGGA**C**UCUGGGCAAAUAUUUGUGGA

AAAUCCCCGGCUGAAGUUGG**C**GGGUAGCACUGCCCUGCACU

CCGCCCACUCCUCCCUCCCU**C**CUCAUGAACCCUUGGCAAAC

GUUCCCCACCACGGUCAGGU**C**UGAGUGAGGGCAGCUCCCCA

CAGAAUCCUGACUGUCGAAG**C**CCCCAAAUAGUUCUGUAGGA

UUUUAGAAAUGCUCUUGCUU**C**UGCCAUUUAUAACAUCAACA

GGGCCGGACAGAGCUGGGGC**C**CCUGGACAGGUCGCGAGGAG

CUGCAGAGAGGGGUUGGUCU**C**CUUCCCUCCUUCAGGUUCCG

GGGUCGGACAGACCUGCGGC**C**CGUGGACAGGUCGCGAGGAC

ACCCGGAGUCCGUGGUUCAC**C**UCGGCUCACUCUUGGCAUUG

ACACAUCUUUGUUAAAACAG**C**ACUGUAUUCAGUAGAGACUG

GCCUGGUGAGCAGCGGCUGA**C**AUGGGCUCGUCCUGCCAUGG

CCACCACGGUCAGGUCUGAG**C**GAGGACAGCUCCCCACCAUG

CCUCCCUCCCUCCUCAUGAA**C**CCUUGGCAAACACAGGACCA

UCUGAGUGAGGAGGGCAGCU**C**CACCACGACUGGCCUGUCUU

GAGUGAGGGCAGUUCCCUAC**C**ACGGUCAGGUCUGAGUGAGG

CUGAGCUUCUGUGCACACAA**C**CCAGUGUUCGCUGAAGCUGU

CCUGAGUGUACUUGGGAGGU**C**AGAGGCUUCCUGGGAGGGGG

UCUGAGCGAGGACAGUUCCC**C**ACAACGGUCAGGUCUGAGUG

AUUUUAAGUGAGUAUUUCCU**C**UCUCCUUUUAUCCUGCUGCA

CAGGGCAGGUUCUGGCCUGG**C**CUUUGACCUCUGUGGGACCC

UUGUGAAAUGACUUUCCUGA**C**CACACAUCUUUGUUAAAACA

ACUGAAAGCACAUAUUCAUG**C**UGGAGUCGACAGUUUCUGUC

AUGGAAGAGCAUUGCGGUAU**C**AUGAGAGGUGUAGAAAGGUG

AUUUAGUGCAGCUGAUGGAC**C**AACACGACACGUCAUCCUCA

UUUAGCACAGUUUUAGGUAC**C**CAGCAAAAUUAAGUGGAAAG

UGCCGCAUCCCCAGCCCAAG**C**UGCCUGGGCCCCCCUGAUGC

UUUUGCAUAAAAAAGGGAAU**C**UGUGAACCUGGGCAGCCCCG

UCUGUGGUUGGGGAGUACAA**C**AUCCUCUAAACCAGUGACCU

UGCUCCUUGUGGGGGCAGUU**C**AGAGACAGAAUCCUGACUGU

GGUUCUGGCCUGGCCUUUGA**C**CUCUGUGGGACCCAGUCCUU

UGAGGGAGGUGUGUGGAUUU**C**ACAAACAGUUUUAGAAAUGC

GCCCCACCCACGCCCUCCCU**C**GUGACCCAGUCCCCUCCAGA

AGGUCUGAGUGAGGGCAGCU**C**CCCACCACGGUCAGGUCUGA

AGGGAGGUGUGUGGAUUUCA**C**AAACAGUUUUAGAAAUGCUC

CUGGUUAUCCCAUUUAAGUA**C**AUCGUUUGCUCCUUGUGGGG

CACAGGACCAUUGACUAUCU**C**UAUAGUUUUGCCUUUUCCAG

CCCUCCCGUCCUCCUUUUGC**C**AGGCUGAACCCCCUCCCCAA

CUUAAGUCACCGUCAGAGCC**C**ACGAUACCCAUUGCCUCGGA

ACCGUCAGAGCCCACGAUAC**C**CAUUGCCUCGGACCUCAGGG

CCACCACGGUCAGGUCUGAG**C**GAGGACAGCUCCCCACCACG

GUUUCUCUGUCCUAAAAAUC**C**CCUCUGCUCCGCCCACUCCU

AGGGAGGUCUGAGGCUUCCC**C**GGUGGGGACUAAGGCUGUCC

GUCGCGAGGACACCAGGUCU**C**ACUGGAGGACGGGCCGGCUU

AAAGACUCACAGGAAUGUGC**C**UAAUGGAAACCCAGUCUGGG

GAAAAAAGUCAUCACAUUUU**C**AUUUAGAGAACAGUUAUUGU

GGAGGAGGAUGCUGUGAGGG**C**CUGUGGCUGCCUCACGUCUG

GCCCACGAUACCCAUUGCCU**C**GGACCUCAGGGGCUUCCUGG

CUGGACAGGUCGCGAGGAGG**C**CGGGUCGGACAGACCUGCGG

UGUCUGUGCUGGGUUUUGGC**C**UUGCUCAGUGGUGUGCAGGG

GCUGACAUGGGCUCGUCCUG**C**CAUGGCCCAAUAAAUCUCGU

GUCCGUGGUUCACCUCGGCU**C**ACUCUUGGCAUUGUCACUGC

CUUUUGCCAGGCUGAACCCC**C**UCCCCAAAGCUGCGUCAGAG

CGGUCAGGUCUGAGCGAGGA**C**AGCUCCCCACCACGGUCAGG

GGGCAGGUGUUUGAUGACGA**C**ACACCCCCCCAUUACAGCAG

UGUACUUGGGAGGUCAGAGG**C**UUCCUGGGAGGGGGCCACGC

GAUGAGGUGCAGCCACAAUG**C**GUGUCUUGUGGAGGAGGAUG

CCAUUUAAGUACAUCGUUUG**C**UCCUUGUGGGGGCAGUUCAG

AUGUGCAUUUACGGUGCCUU**C**GUGUCUUUUCCUGGCUUAGC

GCUCACUCUUGGCAUUGUCA**C**UGCACAGGUUUGGGCAGGUG

GUCUGAGUGAGGACAGUUCC**C**CACCAGGAUCAGGUCUGAGU

GGGACCCAGUCCUUGAAAGA**C**UCACAGGAAUGUGCCUAAUG

UGCUUUCGAGUUGUCAUUCU**C**CCGCGUGGUCUGUGGGGAGA

GUGACUUAAUAUUUUUAGCA**C**AGUUUUAGGUACCCAGCAAA

GAAAAGAAGGGGCUGGUGGG**C**AGGGAAGUGGGAGAGGUGGU

ACUGGAGGACGGGCCGGCUU**C**UCCCAUGCCCUGGGUCCUCC

UGAGUGAGGGCAGCUCCCCA**C**CAUGGUCAGGUCUGAGUGAG

AAAUAGUUCUGUAGGAUGGU**C**UGUCUGUAGGGAUGGCCUGG

CUCCACACAGUGGCUGAUUC**C**CACCCACCAAACAGCGUGCC

AGACAGUUCCCAUAUGGCCC**C**UGCCCCACCCACGCCCUCCC

CCUGACUUGAGUGUCAGGAG**C**CCUCAGAGGAGACACUGCCG

CUCUGCUCCGCCCACUCCUC**C**CUCCCUCCUCAUGAACCCUU

GUGAGGGCAGCUCCCCACCA**C**GGUCAGGUCUGAGUGAGGAC

UCACCUCGGCUCACUCUUGG**C**AUUGUCACUGCACAGGUUUG

AAACCUGUACUUGAGGAAAA**C**CUGAGAAGGUUUUGGGGCAU

UUGGUGUUCCCGCUGUGGUG**C**CUACCCAGCAGGGGCCCUGC

GACCCAGCCUCUCCGCACAG**C**CCCCUCUGUGAUCUUCCCAU

CUCGGCGAGUCCUUUCUUGC**C**UCCUUUCCUGAGUUCUGUGU

CGGUGGGGACUAAGGCUGUC**C**CGGUGGCUAGCAGUGGCUCU

AGAGGGAGGGAAGGGCUGCA**C**AGCAGGGGCUCAGCGCAUGU

CCCUUCACCGUGGAAAUUAU**C**AUGGCUCCCACCUCAGAUGU

CUGGGGUCAUGGGCCCCUCC**C**CAAGUGCUUUCCUGUGAGUG

UGCAGCUGACGCGGCAGCAG**C**AUUCUUUGUUGCUGGAAGAG

AGGUCUUUUUAAAGAUACAG**C**CAUUGCCAGGAAUUUGAUUA

CCCGGGUGCCUGGUGAGCAG**C**GGCUGACAUGGGCUCGUCCU

AUCACCUGGUGCCUGGGAAG**C**UGCAGGCUCCUGAGUGUACU

CCACCCACCAAACAGCGUGC**C**CAGGGGAUUUCUGAGAAAAU

CGGCCCAGUAUAGUCUCAGU**C**CCAAUUUCUUCUUAAGUCAC

GAGAGUCCGAGCUUUACUGU**C**CCUUCACCGUGGAAAUUAUC

AGGCGGCCCAGUGGCUUCUG**C**CGCAUCCCCAGCCCAAGCUG

AUUUCCUCUCUCCUUUUAUC**C**UGCUGCAUGCGGCAGAGGGA

GCCCUCCCUCGUGACCCAGU**C**CCCUCCAGAGCUGUGCAUUU

UGGGGCCCCUGGACAGGUCG**C**GAGGAGGCCGGGUCGGACAG

CUUUGCUCUCUGCUCUUUGA**C**UUCAGCUGAUUUGUAGACUC

AGAGAGGGGUUGGUCUCCUU**C**CCUCCUUCAGGUUCCGUCUG

CCUCCACCCUGACAUGCACA**C**ACCUGGCCCCUGCACACACC

GUGGAAAGUGCAGACAGUUC**C**CAUAUGGCCCCUGCCCCACC

ACGUCAUCCUCACCCGGAGU**C**CGUGGUUCACCUCGGCUCAC

GGCCUCCACACGCGGCACCU**C**CGUCCCUGCUGUCUGGAUGG

UUAAAAUACAGAGUUAUUCA**C**CCCUGAGCUUCUGUGCACAC

CGCAGCUGCGUUGUUCACCC**C**CCACAGUGGAUAAGAGUUCC

GGGCAGCCCCGGGGGCUGGG**C**UCAGGGCAGGUUCUGGCCUG

UUGCCAGGCUGAACCCCCUC**C**CCAAAGCUGCGUCAGAGGAC

AGGUCUGAGUGAGGACAGUU**C**CCCACCAGGAUCAGGUCUGA

GAGCCACCAUGGCUGGCUAG**C**UAUCCCCUUUCUAAAGAGGU

ACCUGUGCUGCCCAGGAGUA**C**AUAUGAUCAACUGCUGAAAA

GGAAGGGGGAAGGAUGGAGC**C**AGUAUUUCUUGGCUCUGGUA

GUGUCACCUGGGCCCUGACU**C**UAACCGCCACCUCCAUGUCC

GUCUCCCACGCCUGCUGUUA**C**UGGUUACCUUUAGAAGGAGU

UGGGCGGGGGGGUCUGUGUA**C**AAGGCAAACGUUUGUGCUGG

UUGAGGUGCAUGGCAGGUGA**C**GUGAGUGGGGCCGCUACAGA

AGCGAUUCUCCUGCCUCAGC**C**UCCCGAGUAGCUAGGAUUAC

AGAUUUCCAGUACAGAGCCC**C**AAUGCUGGGCUGAGAUGCAC

AUGGGAUUUAUAAAAUAGUA**C**UUUACUCACAGGUUUGUUGU

AGUAUUUCUUGGCUCUGGUA**C**AGCUGGUCUAACAGGGUUGG

UAAAACACAAGGGAAAAUUU**C**UCUUGUUUUAAAUUGCUGGC

GGAAUGCCAAGAGUCCACAA**C**AACCUGAUCCAUGAUAGCCC

CCCUCCUUUCAUGCCCGGUG**C**UCCCUGACUCUGCUCCUGGA

UCAUGCCACUGUAAUCUAGC**C**UGGGUGAUGGAGUAAGGCUC

UCCAUGCCUAACGUCAGCGC**C**UGGGGCAGCCCACUCUUCCU

AAGCGAUUCUCCUGCCUCAG**C**CUCCCGAGUAGCUAGGAUUA

UUGAAAUGCUUUUGGAGGAA**C**CAGCUACCUUCUAAAUGAAA

UCAGGGGCGCUGCUCCUUAG**C**CUGGAAGCAUGGGAAUCUGC

AAUCUUCUUCAGGGGCGCUG**C**UCCUUAGCCUGGAAGCAUGG

CUGGGCCCUGACUCUAACCG**C**CACCUCCAUGUCCCUCAUCU

GCUGUGCUUUGUUCCCUAGC**C**CGGCCCCUGGAAUGACUCAC

GUAUACAGACCUCAUAUAGG**C**AAAACCAUGAGGUCAUCAAA

GACCCAAGCAGCCGAUGGUC**C**UAAGACAGGCCAGCUUCCUU

AGUUGGCUCUGUAUCUCAAC**C**AUUUACCCAUAGUAAAGUUU

GCCCAGGCUGGUUUCAAACU**C**CUAAGCUCAAGCAAUCCUCC

GAGUGACUGGCACAUGGAGG**C**UGAUGAUUAAAUGUUUGUUG

AUCCUGACCUACUGACUGAU**C**CAUGCAGGUUGUACUGUCAC

UGUAUUCUAGAUUUCCAGUA**C**AGAGCCCCAAUGCUGGGCUG

CUGGACAGUCAGUGGGGCCU**C**CUGAGAGCCAGAGGUACGGU

UCAUGCCCGGUGCUCCCUGA**C**UCUGCUCCUGGACAGUCAGU

CAUGGCUGGCUAGCUAUCCC**C**UUUCUAAAGAGGUAAGCCUG

UGUGAGAAUUAAAUGAACUG**C**UGUAUGUAAUGCACUUAGAA

GGCAACUGGUUGCUCAGGCU**C**AGGGGCUCCAAUACAUUUUU

GUUGCUCAGGCUCAGGGGCU**C**CAAUACAUUUUUUUUUUUUU

GCCGAUGGUCCUAAGACAGG**C**CAGCUUCCUUGACUGCGUGC

CCAUGCAGGUUGUACUGUCA**C**ACAGCUCACAGAACCAAAUC

GAUUUAACCAUUCCUACCUU**C**CCUCUUGGGUAUGCAGCAAA

GGCAUUAGUGUCUCCCCUCU**C**CCAAAACUACUCACAGAAGU

CAGGCUAGAGUGCAGUGGUG**C**CAUCUCGGCUUACUGCAACC

GAACAGAAAAUUCUUUUUUG**C**UACUUUAUUAGGUUAAUACU

UGAAAAUCAGAGUAGUGAGC**C**UUUCAAGGGCUAAGGUCCAU

AACUGAUCUUCCCACCUCAG**C**CUCCCAAGUAGCUGGGACUA

UGACCCAAGCAGCCGAUGGU**C**CUAAGACAGGCCAGCUUCCU

UUUAGAAGGAGUGACUGGCA**C**AUGGAGGCUGAUGAUUAAAU

CAGUGGCAGGUGGUGCGUCC**C**CAAGGGCUCCCUUUGGCAAG

GGCCCUGACUCUAACCGCCA**C**CUCCAUGUCCCUCAUCUUUC

GGAGGCAGGAGGGAAACCAA**C**UGGAGAGGAGGGUGUGUGUG

AUCAAUUUCCGUAAUAUUUA**C**AUUGGGUGUUUUAUUCUCUG

AUGCCCGGUGCUCCCUGACU**C**UGCUCCUGGACAGUCAGUGG

UCCGGGUCAAAGGGUAGAAA**C**AAUUUAUGGCAUUUGAUUUG

UCUAACCGCCACCUCCAUGU**C**CCUCAUCUUUCAGGCAUUAG

GGGACCCACGUCAUCUACAU**C**CUGACCUACUGACUGAUCCA

CAAGUAGGCCUCAAGGCCAA**C**UGUCAUCAAUUUCCGUAAUA

GAAAAGUGGAAAAGUGGCUC**C**UUUGGAGGGUGGCAUGUUAG

AACUCCUGGGCUCAAGUGAU**C**CGCUGACCUUAACCUCCCAA

UGGCCAACAUAGUGAAACCC**C**GCCUCUAAGAAAAACAUAAA

GCUGAAAACAAACAGCCAAG**C**AAACUUGAAUGCCUGAUGUG

AAACUCGGAAUGCCAAGAGU**C**CACAACAACCUGAUCCAUGA

CCUCCCUCUCUCUUUCCUUU**C**CUUCCCCCUUCACUUCCUUC

UUGGGGAGCGAAGAUCUGUU**C**CAUCACCUGCUUCUGCCUGU

GAGAGGUGAAAGGCAGUUGG**C**UUGAGUGUUGCAGGAAGGAA

CUUCAUGAGAGCCUCAGGUG**C**CUAAAUGACUCUCCCUUCUU

UCUCCUUUCCCUCUCUCUAU**C**UCUCUCUCUGUCUCUCUCUU

CCCAGUAAAAUCACCAUUGC**C**UGCAUAUCCCGGAUCUUCAA

AAAAGAAUGUAUUUACUUAG**C**UAUUUUUUGCUUAUUGUUCA

UAAGAUUUAACCAUUCCUAC**C**UUCCCUCUUGGGUAUGCAGC

GCAUGGGAAUCUGCUAAGCC**C**CUGGGUAGCCCAGCUUGAGG

UGCCCAGGAGUACAUAUGAU**C**AACUGCUGAAAACAAACAGC

CCCAUAGUAAAGUUUAUCUC**C**UCUCCUCUCCUCUCCUUUCC

GAGGUAAGCCUGCCUGAGUA**C**AAAUCCCAUACAAAAAGAAU

ACAGGGCUCUUUAAAGGCAA**C**UGGUUGCUCAGGCUCAGGGG

UGCCUAAAUGACUCUCCCUU**C**UUUUGGUAAAUGUAUUCUAG

GGUCUGAAGGACCUGCCUCC**C**CAGGGAUUCUCAUCUGGGUU

GCUGUUUUCUAUUAUCUGAA**C**AAUACUUACCUAAUUAAGCU

UAAUCAAGCAGAGCAUGGGU**C**CUAACACCCCAGCUGCCGGG

CCUGCCUCCCCAGGGAUUCU**C**AUCUGGGUUUUCAAUACUCU

GCCAGUGGCAGGUGGUGCGU**C**CCCAAGGGCUCCCUUUGGCA

UUGCUAGGUGCUCAGCUAGG**C**UCACUCACUCUAAUUAAAGG

CAAAUAUAUGAAGUUACAUA**C**UUUUGAGUUAUUUACAUGUA

UCACUGCUGUUUUCUAUUAU**C**UGAACAAUACUUACCUAAUU

GUGCCCUUAGGGGAUCUCUC**C**UUUGGGCUAAGGUCCUCUGA

UCUCGAGGGAAUGAGUGGGG**C**AUAAAAAUUCUGUUUCUCAG

GUCACUAGUCUGUAAUUUUU**C**CUUUCCUCUUUCUUUUCCCU

AGAUGGCAGGCCAGUCCUAG**C**UGUGCUUUGUUCCCUAGCCC

UCUUUCAGGCAUUAGUGUCU**C**CCCUCUCCCAAAACUACUCA

UGGAGGGUCUGAAGGACCUG**C**CUCCCCAGGGAUUCUCAUCU

UAACUUUUCCUCUGCCUCCC**C**ACUUAGUCGUAACCCUUGUG

AACACCCCAGCUGCCGGGAA**C**CACUGUCCAGGCAGUUCUCG

UUAAAUUGCUGGCUAGUUUC**C**ACCUCAUUCCGUAUUGACUU

UGUUCCAUCACCUGCUUCUG**C**CUGUCGCCUCCACGCACCCC

CCCAAUGCUGGGCUGAGAUG**C**ACUUGCUAGGUGCUCAGCUA

UUAUGAAAUGAAGAGUUAUU**C**CCGAUCGAAAAAAAUCUUUG

GUGUCUCUGUGCUUCAGUUU**C**UCAUGUAUAAAAUGGGAUUU

UAUCUUUCUGUAUUUGAUUA**C**UAGUUAUAUUUAUUAUUUUG

UGUGCUUGUGCCUGUCCGUG**C**UUGAGGUGCAUGGCAGGUGA

CUGGGAUUACAGGCAUGAGC**C**ACCACACUCGGCCCCAGUAC

GGUUGUACUGUCACACAGCU**C**ACAGAACCAAAUCCUUUAUC

ACUUCCUUCCUUCCUUUUUC**C**UUCUAUUGUUUUAAUGUUCG

UCUUCAUUUAAAGGGAAAAU**C**UGUAAAUGUAUACUCUGUCU

AUGCCAAGAGUCCACAACAA**C**CUGAUCCAUGAUAGCCCUUG

ACGUCAGCGCCUGGGGCAGC**C**CACUCUUCCUGGAAGAUGGG

AGAUGCACUUGCUAGGUGCU**C**AGCUAGGCUCACUCACUCUA

GCCUGAGUACAAAUCCCAUA**C**AAAAAGAAUGUAUUUACUUA

AGGCCGAAGGCUAACUUUUC**C**UCUGCCUCCCCACUUAGUCG

AUGAAAUGAAGAGUUAUUCC**C**GAUCGAAAAAAAUCUUUGGA

AGCAGUUUUCUUAGCUGUUC**C**CUAUUGUGGGGCUUUUAGGU

UAAAGUUUAUCUCCUCUCCU**C**UCCUCUCCUUUCCCUCUCUC

CUGUCGCCUCCACGCACCCC**C**CCAGAAACCAGGAAUCAAGU

GCUUUGUUCCCUAGCCCGGC**C**CCUGGAAUGACUCACAAUGC

GCAGGAGAGUCGCUUGAACU**C**GGGAGGUGGAGGUGGCAGUA

UAAUGUUCGUAUUGUCACCU**C**UGUUAAUCUUUUCCUCUGAC

CAACAUACUUGUGUGUAUAG**C**UUCCUUCUUUUGGGUUAUUU

GAUGCAGAAUAUAAAUUAAU**C**CAUGUACUUCUCUCAAGAUU

CUCUGUAUCUCAACCAUUUA**C**CCAUAGUAAAGUUUAUCUCC

UUUCCUUCCCCCUUCACUUC**C**UUCCUUCCUUUUUCCUUCUA

GUGAGAUGGAAACGUUAGGC**C**AGUCCUUUUCUGGGUAUAUC

GUGCUGGCUGCCCCUCCUUU**C**AUGCCCGGUGCUCCCUGACU

GUCUCUGUGCUUCAGUUUCU**C**AUGUAUAAAAUGGGAUUUAU

CCGAUGGUCCUAAGACAGGC**C**AGCUUCCUUGACUGCGUGCU

CCAAAGUGCCGGGAUUACAA**C**AUGAGCCACCAUGGCUGGCU

UUGGCCAGGCUGGUCUCAAA**C**UCCUGGGCUCAAGUGAUCCG

AAAAUCUGUAAAUGUAUACU**C**UGUCUCCCACGCCUGCUGUU

UGGGGAGCGAAGAUCUGUUC**C**AUCACCUGCUUCUGCCUGUC

UGUGGAAACUUUGAAGAUGG**C**AGGAGAGGUGAAAGGCAGUU

CAAGACCUUCAUGAGAGCCU**C**AGGUGCCUAAAUGACUCUCC

CUGCCAUGAUUUUCCCAACG**C**UUUUCUCCAGAGCCCUUAGU

CGGUGCUCCCUGACUCUGCU**C**CUGGACAGUCAGUGGGGCCU

UUUACAUGUAGUGUGAAACA**C**AAAAUGAGGGGUUUAGGAAG

CAGCUAAUUCGAUUUUUUUU**C**CAUAGAGGUGGGGGGUUCUC

GCUUUUGGAGGAACCAGCUA**C**CUUCUAAAUGAAAAAUAUGG

GGAAUUAAGAAAGGCCUUUU**C**AGGCUGGGCAUGGUGGCUCA

AGUCCUAGCUGUGCUUUGUU**C**CCUAGCCCGGCCCCUGGAAU

AGAACCAAAUCCUUUAUCUC**C**ACCCACACUUAAGGAUACAU

GGCCAGGCUGGUCUCAAACU**C**CUGGGCUCAAGUGAUCCGCU

AGUAGCUAGGAUUACAGGCA**C**CUGUCACCAUGCCCAGCUAA

UCUUUUUGGUUAAUUUAUUC**C**CAGAAGUGAGAUUACUGGGU

CCUGGAGGCAGGUGUCCAUG**C**CUAACGUCAGCGCCUGGGGC

AAAGGCCUUUUCAGGCUGGG**C**AUGGUGGCUCACGCCUGUAA

GGAGCAGGGGCUCAGGAAGG**C**CUCACCUGUGUCCCUAGACA

UUCUGGGUAUAUCUGAAAAU**C**AGAGUAGUGAGCCUUUCAAG

UGUCUCAAAAAAAAAAAGGC**C**UUUUCAAAAGUAAAAGGGGG

AGGCUUAGGCAGGAGAGUCG**C**UUGAACUCGGGAGGUGGAGG

UCCUAGCUGUGCUUUGUUCC**C**UAGCCCGGCCCCUGGAAUGA

AGUGUCCCACUGAAGGCUGA**C**ACCUCUUCAUUUAAAGGGAA

AGCUGUUCCCUAUUGUGGGG**C**UUUUAGGUUGCUUCAAUUUU

UCCUUUCCUCUUUCUUUUCC**C**UCCCUCUCUCUUUCCUUUCC

AGCAAGUACUAUGCUUAAUA**C**UUAACUUGUUAUAUUUGUUA

AUGAUUUUCCCAACGCUUUU**C**UCCAGAGCCCUUAGUCAAGU

UGGGUUGUGGGCGGGGGGGU**C**UGUGUACAAGGCAAACGUUU

AAUCCUCCUACCUCAGCCUC**C**CAAAGUGCCGGGAUUACAAC

CAACAUUUUUGGCAUUGUGU**C**UUUGUUCUGUGGGAUUCAUU

CAUCACCUGCUUCUGCCUGU**C**GCCUCCACGCACCCCCCCAG

GUCCAUGCCUAACGUCAGCG**C**CUGGGGCAGCCCACUCUUCC

UCUGCUAAGCCCCUGGGUAG**C**CCAGCUUGAGGCGUUUGAUC

GGAGGAGGCAGGAGGGAAAC**C**AACUGGAGAGGAGGGUGUGU

GUCCUAGCUGUGCUUUGUUC**C**CUAGCCCGGCCCCUGGAAUG

ACCUCUGUUAAUCUUUUCCU**C**UGACUUUGGUUUAUAAGCCA

UCAAUACUCUACUGUGUCUC**C**UGUAGUUUCCUGUGGUUGCU

ACUCCUAAGCUCAAGCAAUC**C**UCCUACCUCAGCCUCCCAAA

AAAGUAGUGGGCUGUGAAUU**C**AGGGCUACUUGGAGCCCUUA

GCUGGGCAUGGUGGCUCACG**C**CUGUAAUCCCAGCACUUUGG

UCCUCUCCUCUCCUUUCCCU**C**UCUCUAUCUCUCUCUCUGUC

CCUCAGAGGAAGGAGUGCUU**C**UUAGCAGUGAGCAUUGUGGA

AACAACCUGAUCCAUGAUAG**C**CCUUGAUUUCUUGGCCAAAU

CAGUGAGUACCUGUGCUGCC**C**AGGAGUACAUAUGAUCAACU

GCAGUAAGCUGAGAUCAUGC**C**ACUGUAAUCUAGCCUGGGUG

AGAAAAACAUAAAAAUUAGC**C**AGGCGUGUGGUGUGCACCUG

CAGGUUGUACUGUCACACAG**C**UCACAGAACCAAAUCCUUUA

ACCACUGUCCAGGCAGUUCU**C**GAGGGAAUGAGUGGGGCAUA

CUCUCUUUCCUUUCCUUCCC**C**CUUCACUUCCUUCCUUCCUU

CGCUUUGCCCAAGUAUUGUA**C**CAAGUUUUACUGCUCCCAGC

GCCUGUCCGUGCUUGAGGUG**C**AUGGCAGGUGACGUGAGUGG

UGCACCUGUAAUCCCAGCUA**C**UCAGGAGGCUUAGGCAGGAG

CAGGCAUGAGCCACCACACU**C**GGCCCCAGUACAUUUAAUUU

ACAUGAGCCACCAUGGCUGG**C**UAGCUAUCCCCUUUCUAAAG

UGUCCCUAGACACCUUCCAG**C**AGAACCUCAGAGGAAGGAGU

GCCUCCCCAGGGAUUCUCAU**C**UGGGUUUUCAAUACUCUACU

GCCAUCUCGGCUUACUGCAA**C**CUCUGCCUCCCAGGUUCAAG

CAGGUGCAUUCCACCAUGCC**C**AGCUAAUUCGAUUUUUUUUC

UCCUGGACAGUCAGUGGGGC**C**UCCUGAGAGCCAGAGGUACG

ACAACCUGAUCCAUGAUAGC**C**CUUGAUUUCUUGGCCAAAUC

AGCUCAAGCAAUCCUCCUAC**C**UCAGCCUCCCAAAGUGCCGG

GGUGCCAUCUCGGCUUACUG**C**AACCUCUGCCUCCCAGGUUC

UUCUUUGAUUUCCAGUGAGG**C**UAUCUUUCUGUAUUUGAUUA

CUGUAUCUCAACCAUUUACC**C**AUAGUAAAGUUUAUCUCCUC

UUUGGUUUGGUUUAGAUUAG**C**CAGCGGUUCUGUGGCUUUUG

UUUUCUCCAGAGCCCUUAGU**C**AAGUAGGCCUCAAGGCCAAC

CACAGAACCAAAUCCUUUAU**C**UCCACCCACACUUAAGGAUA

AAUGCUUUUGGAGGAACCAG**C**UACCUUCUAAAUGAAAAAUA

CUGUUAACGUGUUUCUUGAC**C**ACAGCGAAACAGUGGGUAGA

CUAUCUCUCUCUCUGUCUCU**C**UCUUUGAGACAGGGUCUUUC

GUGAAAGAGAAAAGGGAUGA**C**UCAUGGCAUUAUGGUGAUAU

UGUGUAACCUGGGCCAGCUG**C**UCAGUGUCUCUGUGCUUCAG

CCUGUGUCCCUAGACACCUU**C**CAGCAGAACCUCAGAGGAAG

CCUUGACUGCGUGCUGGCUG**C**CCCUCCUUUCAUGCCCGGUG

GAUGGAGUAAGGCUCUGUCU**C**AAAAAAAAAAAGGCCUUUUC

GGAGGGUCUGAAGGACCUGC**C**UCCCCAGGGAUUCUCAUCUG

GAUUCUCCUGCCUCAGCCUC**C**CGAGUAGCUAGGAUUACAGG

CUUAGAGUAGACCCUAUCAC**C**UCCUGCUGUCACCAGUGAGU

UGGCCAGGCGCAGUGGCUCA**C**GCCUGUAAUCCCAGCACUUU

GGGCACUGCUUCAGCUCAGG**C**AGCAGGGAGACAACGUUCCC

UUAUCCUUCCAGAAAUGUUU**C**UGAUAUAGAAGAUUAGUUUC

GGCACAGUCCUGGCCUGACU**C**CAGGGAAGACGCACUCUGGA

GCUCACGCCUGUAAUCCCAG**C**ACUUUGGGAGGCCAAGGUGG

UAGCAGCUGCUCACAGCUGG**C**UUGGGGAUGUUGGGCCACCA

UGAAUGAGGUGCUGAAUAGA**C**UGCACCUGGCUAUCCCAACC

GUAGAGCUGUACUUCAGAAA**C**CUGAGCCAGUAGGGAGGGCU

AGGGUUGUGACUCUUCUGGG**C**GCCCCAGGCACCGUUGCCCU

AGCCAGGGUGACCUUGACCU**C**CUGACCUCAUGAUCCACCCG

UGCUAUUCUUGUUUUUCCCG**C**AAUGGGGGACUUAUCUGAGG

UCAAAAAAAAAGAUGGCACU**C**CUGAUGCCAUGGUCGCACUC

UCCACCUCCACCAAGCCCUG**C**UGGGAAGGGGUAGCAGCUGC

AAUGGGCACACAUCGUUACG**C**UGUGUCUUCAUGUUUUACUG

GGGGCUCCACCUCCACCAAG**C**CCUGCUGGGAAGGGGUAGCA

GCCACAGUGCUGGGUUUCAG**C**UACAGGUACUCAGGGUAGCU

CAUCCAGCUUCACACACUCG**C**AGGUGGAUAUUGUGGGUGGG

GGUUGUGAGGGGAUGCUUUG**C**UCUUCUCACCCCAAACAACU

GUUGGAGGGCACCGUCUGUG**C**AGAGGGUCACUGUGGUGGGA

GGUGUCAGGUCUGACAUGGU**C**UGCAAGCAGCUGCCACAGUG

GAGAACCUCCUCCUGGGUCC**C**GUUGCAUCCCAGCUGUAGGG

GUAUCCCAGACUCUCUGAUG**C**UAGGUGAGGUGGCAGCAAGG

GAGUAUAGCCGCCCCAUGGG**C**CCAACAGACAGGGCCCAGUG

AAAAGAUGGCACUCCUGAUG**C**CAUGGUCGCACUCAGGAUUU

CAUGUGGUGUUGACAACGUU**C**CUGUCCCACAGACGCCUUCC

GGUGGCAGGGCUGCUGCCAU**C**CUCUUGCUUACCCCCUCACC

UUUUCCAGAAGCCUCCCAUC**C**AGCUUCACACACUCGCAGGU

GGAUAUGUGACCAUUCUGGG**C**AUUUCCCAGGAAGCAGGGCA

CUGCAAAUGUGUCUGCACUU**C**ACCAUGUGGUGUUGACAACG

GGCUUGCGAUAGGCUUUUGG**C**UCAUCGAGUGGAAACCAAAA

CAGCAGGUGCUGUCUGCGUG**C**CUUUUCCAGAAGCCUCCCAU

UGCCCUGGGAUACAAGGUAC**C**UCUUCAGCAUAGACGCUGGG

UGCUGUCUGCGUGCCUUUUC**C**AGAAGCCUCCCAUCCAGCUU

CAUUCUCCUGCCUCAGCCUC**C**CGAGUAGCUGGGACUACAGG

UGCCUGUUACUCCUGUGAUG**C**CCUCUGGAGCUCCCCCUCAG

GCUGGGACUACAGGCGGCCG**C**CACCACGCCUGGCUAAUUUU

GUCAUUUGGGCCCUAGAGGA**C**AGGGAGGAGGACAGCAGUGA

AUAGACGCUGGGAUAUGUGA**C**CAUUCUGGGCAUUUCCCAGG

GUUUUUCCCGCAAUGGGGGA**C**UUAUCUGAGGGGAUCUUUCU

UCAUGCCACUGCACUACAGC**C**UGGGCAGCAAGAGCGAGAUA

CUGGGACAGCCGUGGGCUCA**C**AGUACUGUCAGCAGGUGCUG

ACAGCCGUGGGCUCACAGUA**C**UGUCAGCAGGUGCUGUCUGC

GGCAGGGCUGCUGCCAUCCU**C**UUGCUUACCCCCUCACCGUA

CGUCUUGCGUUUUUCUGCUC**C**CCAGGGUGCCUGUUACUCCU

GCUGCCACAGUGCUGGGUUU**C**AGCUACAGGUACUCAGGGUA

UCAGAGAAUGUGGCCCCCUA**C**GUGUUAGUUACCUUGAGGGA

CCUCUUGCUUACCCCCUCAC**C**GUAGGGAGAACCUCCUCCUG

GGAGGUUUGAGCCUGGGAAG**C**AGGGUAGAGCUGUACUUCAG

CUGGGUUUCAGCUACAGGUA**C**UCAGGGUAGCUGUGGGACUG

UUUUUAAGACUUUGAAACAA**C**CACAGAUGUUCACAUUUGCA

AGGGGAGCUGUGGCCACUUC**C**CCUGAGCAAGACAUACUCCA

GGGAGCUGUGGCCACUUCCC**C**UGAGCAAGACAUACUCCAGC

UGCUUGUAAUCCCAGCUACU**C**GGGAGGCUGAGGCAGGAGAA

GCUCCUCCUGGAACCUGUAG**C**UGGAGGCUGCUGGAGGAUGC

GUGACCAUUCUGGGCAUUUC**C**CAGGAAGCAGGGCACUGCUU

UUGUUCGUUAUUCUGAUUGC**C**UUUGUUGUGGGGGACAGAUG

UGUGCCUAUCAUGGCCUGGC**C**UUGGGGUGCAGGUUUACUCU

UUGUGACUCUUCUGGGCGCC**C**CAGGCACCGUUGCCCUGGGA

AGCAAGGACCCCAGAAUGGA**C**UAGUCCAGCUGUCAUUUGGG

GCAGGUGCCUCGGCUCAGCC**C**CUGCCCUGUUUCCCUGGGGU

AGAACAGCUUUUGCAGGAGG**C**AGUCCGCUGCUGGAUUUCAC

CUCCUGGGUCCCGUUGCAUC**C**CAGCUGUAGGGUGGGUUGCU

GGAUCUCUGAUCCUGAAGCA**C**CCCUAGCAGCUUAAGCCCAC

UCCUGCUAUUCUUGUUUUUC**C**CGCAAUGGGGGACUUAUCUG

GUAGUUUGGAGGCAGUUUAG**C**CUCAGGGAUGAAGGGGAGCU

GAGUUCUUGACCAGCCUGGG**C**AACAUAGUGGGACCCUGUCU

GCAGUGGCGUGAUCUCUGCU**C**AAUGCAAGCUCCACCUCCCA

GUCACUGUUAGUUUAUCCUU**C**CAGAAAUGUUUCUGAUAUAG

UUUGGGCUGUAUUUCAUCGU**C**UUGCGUUUUUCUGCUCCCCA

CCCCAGGGUGCCUGUUACUC**C**UGUGAUGCCCUCUGGAGCUC

UCGCACUCAGGAAUUUGUUG**C**UCGGUUAGAGUCCUGAGGGG

GGACCCUGGGAUGGUGGGGC**C**CAGCAGUAUCCCAGACUCUC

UCGUUUUUUCUUUGCAUUCC**C**AGAAAAUAUUUACAGAUUUG

CCUCUGUCUUUCCUUGUGUC**C**UCGGCGUGGUCGGGCACUGA

GAUUCUUGUCAGAUACAUUG**C**AAAUUUUUUUCUCUUUUUGU

UGGAUAUUGUGGGUGGGCCU**C**AGUUUCACCUUCUUGCUUAC

GAAGAAAGUGCAGAACGCCA**C**AUGGCACCCGUUUCCCACUG

AGUAUAGCCGCCCCAUGGGC**C**CAACAGACAGGGCCCAGUGU

GCCUGACUCCAGGGAAGACG**C**ACUCUGGAGGUUUGAGCCUG

CUCAGAAUAUAGGGUUAGUC**C**AGCUCCAGGGAGCAGGUGCU

CAGCUCCUUCUGUGUGGGAG**C**ACAGCUGUGAGGAUGCCAGG

CUCCUGACCUCAUGAUCCAC**C**CGCCUCGGCCUCCCAAAGGC

AUGGGCCCAACAGACAGGGC**C**CAGUGUCAGCUCCUUCUGUG

UAGCCUUAGGGUCUUGUGUG**C**CUAUCAUGGCCUGGCCUUGG

GUGUCCUCGGCGUGGUCGGG**C**ACUGACUGUCAGUCUUGUGU

CUGAGGGGCAACAUUGCCUC**C**GGUUCGUGAAGGGCAGUUGG

UUGGCUCAUCGAGUGGAAAC**C**AAAAUGAAGGGAAAUAAAGU

UGGUGAGGGCUGUGGGUGUC**C**AGAGUGGUGGCAGGGCUGCU

UGUGACUCUUCUGGGCGCCC**C**AGGCACCGUUGCCCUGGGAU

CAACGUUCCUGUCCCACAGA**C**GCCUUCCUGGUGUGUGCUGC

AAAUUUUUUUCUCUUUUUGU**C**ACUUGCCUUUUUAACUUCAA

UUUGAUGUCAUUUGUGUUUU**C**UUGAUCAUAGAAGAAUAUGA

CUCGUUUUUUCUUUGCAUUC**C**CAGAAAAUAUUUACAGAUUU

GCCUGGGAAGCAGGGUAGAG**C**UGUACUUCAGAAACCUGAGC

GCACUCAGGAAUUUGUUGCU**C**GGUUAGAGUCCUGAGGGGCA

UUUUUGGCUGGGGGAAGUAG**C**UCACGCCUGUAAUCCCAGCA

GGAGGGACGAUAAGGUAAGA**C**GGCCCCUGGGACAGCCGUGG

AUUGCUUAAGCCCAGGAGUU**C**UUGACCAGCCUGGGCAACAU

AUGAGGUGCUGAAUAGACUG**C**ACCUGGCUAUCCCAACCAGG

UGAUCCACCCGCCUCGGCCU**C**CCAAAGGCUGGGAUUACAGG

UCUCUGAUCCUGAAGCACCC**C**UAGCAGCUUAAGCCCACAGG

GACAGCAGUGACUCCAUUUC**C**UGGAAAGAGUGGUGACUCUG

CGCACUCUGGAGGUUUGAGC**C**UGGGAAGCAGGGUAGAGCUG

ACCUCAUGAUCCACCCGCCU**C**GGCCUCCCAAAGGCUGGGAU

GGUUGUGACUCUUCUGGGCG**C**CCCAGGCACCGUUGCCCUGG

GCAGAACGCCACAUGGCACC**C**GUUUCCCACUGCAUGACGUG

UGCCUCAGCCUCCCGAGUAG**C**UGGGACUACAGGCGGCCGCC

AGCAAGACAUACUCCAGCCG**C**AGUUCCAGUCCCAAGAUGAU

UGAGCAAGACAUACUCCAGC**C**GCAGUUCCAGUCCCAAGAUG

GAUUUAAAAAGAUGGCACUC**C**UGAUGCCAUGGUCGCACUCA

UUUGCUCUUCUCACCCCAAA**C**AACUUUACCCUAAAGCAGCU

ACACAGAUGGAAAUCUGUUU**C**AUUCUUUAAAUUUUCAUUUU

ACGCUGGAGGCUGUGUCCUU**C**CCUCUGUCUUUCCUUGUGUC

ACAGGGUUUCACCAUGUUAG**C**CAGGGUGACCUUGACCUCCU

GCGAUAGGCUUUUGGCUCAU**C**GAGUGGAAACCAAAAUGAAG

CAGGUGCCUCGGCUCAGCCC**C**UGCCCUGUUUCCCUGGGGUG

GGCACUCCUGAUGCCAUGGU**C**GCACUCAGGAAUUUGUUGCU

GCCUGGGCAACAUAGUGGGA**C**CCUGUCUCUAUAAAAAAUAA

AGGGGCUACUAGUCGGCCAU**C**UUUUUGAUGUCAUUUGUGUU

GGGAGAAAAUAGUUUUCUUU**C**UAGACAUUGUUAACACUUGG

GUUGGGUGCUAGAGCAAGGG**C**UAGUAGAGGGGCUCCACCUC

CCUUCUAAGACCCACUUUGC**C**CCCCGUGGACAGUGGGUUGU

GCCAGGCGCAGUGGCUCACG**C**CUGUAAUCCCAGCACUUUGG

CGCAGUAGCGAGGCGGGUGG**C**GUGCGAGAGUGGGGCGUGAA

AACAUAGUGGGACCCUGUCU**C**UAUAAAAAAUAAAUAAAAUA

CCAACGUGGUGAAACUCCGU**C**UCUCCUAAAAAUACAAAAUU

UGGGUCCCGUUGCAUCCCAG**C**UGUAGGGUGGGUUGCUGGCG

GUGCCUCGGCUCAGCCCCUG**C**CCUGUUUCCCUGGGGUGUGG

UCUUGCGUUUUUCUGCUCCC**C**AGGGUGCCUGUUACUCCUGU

CUGUCAGCAGGUGCUGUCUG**C**GUGCCUUUUCCAGAAGCCUC

GAUACAAGGUACCUCUUCAG**C**AUAGACGCUGGGAUAUGUGA

CACACAUCGUUACGCUGUGU**C**UUCAUGUUUUACUGCAAAUG

CAGGAAGCAGGGCACUGCUU**C**AGCUCAGGCAGCAGGGAGAC

CUGCCAUCCUCUUGCUUACC**C**CCUCACCGUAGGGAGAACCU

GUGGCUCACGCCUGUAAUCC**C**AGCACUUUGGGAGGCUGAGG

GGACAGCAGUGACUCCAUUU**C**CUGGAAAGAGUGGUGACUCU

GGAACUGGAAAUGAAUAAUA**C**AUCUCUCGUUUUUUCUUUGC

CUUUAAACACAGAUGGAAAU**C**UGUUUCAUUCUUUAAAUUUU

UGUCCUUCCCUCUGUCUUUC**C**UUGUGUCCUCGGCGUGGUCG

UUUUAUCUCAAAUAAAAGGA**C**AUUUCUGGCCAGGCGCAGUG

GGGUGACCUUGACCUCCUGA**C**CUCAUGAUCCACCCGCCUCG

CUGUGAUGCCCUCUGGAGCU**C**CCCCUCAGUUAUUAACAUGU

UCUGUGGCUCCUCCUGGAAC**C**UGUAGCUGGAGGCUGCUGGA

GAUACAUUGCAAAUUUUUUU**C**UCUUUUUGUCACUUGCCUUU

AGAUUAGUUUCUAAAAAGUU**C**UUUAAACACAGAUGGAAAUC

AGGGAGAAAAGUCUGAAAAG**C**AGAACAGCUUUUGCAGGAGG

UUGGGGUGCAGGUUUACUCU**C**UGUGGUGGAUCUCUGAUCCU

GCAGAACUGUACAGUUUGUU**C**CAUAGGGAACUGGAAAUGAA

GGAGUUCGAGACCAGCCUGG**C**CAACGUGGUGAAACUCCGUC

GGCUGUAUUUCAUCGUCUUG**C**GUUUUUCUGCUCCCCAGGGU

CCCACAGGGCAAGGCUGUGG**C**UGUGGCUCCACCCGUGGGGU

GGGAUGAAGGGGAGCUGUGG**C**CACUUCCCCUGAGCAAGACA

AGAUGCACCUUCCCUCAGGG**C**CGCUUCCACCUGGGCCUCAG

GAAAUGAAUAAUACAUCUCU**C**GUUUUUUCUUUGCAUUCCCA

CAGCUUUUGCAGGAGGCAGU**C**CGCUGCUGGAUUUCACAUGC

CUUUUCCAGAAGCCUCCCAU**C**CAGCUUCACACACUCGCAGG

UUCACAUGCUGUCCUAAUUA**C**UUUGUAUUUCAUGUUGUUUU

UAAGCCCAGGAGUUCUUGAC**C**AGCCUGGGCAACAUAGUGGG

GAAAAGUCUGAAAAGCAGAA**C**AGCUUUUGCAGGAGGCAGUC

CCAUUCUCCUGCCUCAGCCU**C**CCGAGUAGCUGGGACUACAG

CUUGCGAUAGGCUUUUGGCU**C**AUCGAGUGGAAACCAAAAUG

AGGGAGGAGGACAGCAGUGA**C**UCCAUUUCCUGGAAAGAGUG

UCUAAGACCCACUUUGCCCC**C**CGUGGACAGUGGGUUGUGAG

GCCUCAGAGAAUGUGGCCCC**C**UACGUGUUAGUUACCUUGAG

AAUAGAGACCAGCACCCUGA**C**UGAGGAAGGUGUUAUUAAAG

ACUGGAGAUGCACCUUCCCU**C**AGGGCCGCUUCCACCUGGGC

CGUGCCUUUUCCAGAAGCCU**C**CCAUCCAGCUUCACACACUC

GAGGACAGGGAGGAGGACAG**C**AGUGACUCCAUUUCCUGGAA

UUUGGCUCAUCGAGUGGAAA**C**CAAAAUGAAGGGAAAUAAAG

GUCAGGUAUUAGUCUUUUUA**C**UGUCUCUUGUUUGAUUCAAG

CCCCAUGGUAUGUGGCUGGU**C**AGCCUGGGCCUGAGGGUUGU

AGUGGCUCACGCCUGUAAUC**C**CAGCACUUUGGGAGGCUGAG

UUUUUACGUUAUUAGGCUUG**C**GAUAGGCUUUUGGCUCAUCG

GUGGAUCUCUGAUCCUGAAG**C**ACCCCUAGCAGCUUAAGCCC

AGAAGCCUCCCAUCCAGCUU**C**ACACACUCGCAGGUGGAUAU

UUACCUUGAGGGAAAAGGCU**C**AGUGGCUCUGAUCUGUGGCU

CCCCUAGCAGCUUAAGCCCA**C**AGGGCAAGGCUGUGGCUGUG

AUUAAAGUUAAAACAGAGGU**C**AGUGCUGCCUUAAGUCAGGU

GGGAAGGGGUAGCAGCUGCU**C**ACAGCUGGCUUGGGGAUGUU

GCUGGCUUGGGGAUGUUGGG**C**CACCAGGUUUGGGUAGUUUG

GUGGGGCAGGUGCCUCGGCU**C**AGCCCCUGCCCUGUUUCCCU

ACAUGGUCUGCAAGCAGCUG**C**CACAGUGCUGGGUUUCAGCU

CUGGAGGCUGCUGGAGGAUG**C**GUUUGUGACUGUGCCUUCUU

CACCCCUAGCAGCUUAAGCC**C**ACAGGGCAAGGCUGUGGCUG

GGCUCUGAUCUGUGGCUCCU**C**CUGGAACCUGUAGCUGGAGG

GGUACCGUUGGGUGCUAGAG**C**AAGGGCUAGUAGAGGGGCUC

ACAUGUAGUUGUUCGUUAUU**C**UGAUUGCCUUUGUUGUGGGG

GCACAGUCCUGGCCUGACUC**C**AGGGAAGACGCACUCUGGAG

CACUCCUGAUGCCAUGGUCG**C**ACUCAGGAAUUUGUUGCUCG

ACCUUCCCUCAGGGCCGCUU**C**CACCUGGGCCUCAGAGAAUG

AGCUCCCCCUCAGUUAUUAA**C**AUGUAGUUGUUCGUUAUUCU

GUGUCCUUCCCUCUGUCUUU**C**CUUGUGUCCUCGGCGUGGUC

AAGAUGAUGGAGUAUAGCCG**C**CCCAUGGGCCCAACAGACAG

GACUCCAGGGAAGACGCACU**C**UGGAGGUUUGAGCCUGGGAA

GACAACGUUCCCUGAGAGGC**C**ACGGUGCUAUUUACCAGGAG

GAUGAUGGAGUAUAGCCGCC**C**CAUGGGCCCAACAGACAGGG

UGCACCUUCCCUCAGGGCCG**C**UUCCACCUGGGCCUCAGAGA

GCUGUGAGGAUGCCAGGGGG**C**UCCGGUGGUGAGGGCUGUGG

ACAGACCCUUCUAAGACCCA**C**UUUGCCCCCCGUGGACAGUG

AAGCAGCUGUUGUCUCUGGC**C**UUUUAAUAUUUCGGUAGCAA

ACAGUGGAGGCCUCCUGGAU**C**UCUAGGUCUCAGGGCCUCUC

CCCCCUGUCUUUGGCCCCCA**C**CAGGGGAGCCUCAGGAAAAC

UGUCCCGCAGGCAUCUGCAG**C**AUGUGGGGCGGCUCCCACUA

GGUGCCCGAGCCCAGGACUC**C**UUGGAAAACAUCCCCUGCUG

GGGCCAUGGGAGGGGUGGUC**C**CAUGGGGAGGGUCGGCCCAG

GCACCUGCCCCCAGGUGAGC**C**CACAGCUGCUGGGCAGACCC

AGGGGCUCCCCAGCAGGGCU**C**CCCCUCUCCACGUCCAUUGG

CAGGCAGGCUCUUGUGGCCA**C**CCGGGGCUUUGGGCCAUGAG

CCCCAAAAGUUCUCCAGGGC**C**UUCCAUCCCGGGGGGAAGCA

UCCCGGGGGGAAGCAGGCUC**C**AGGCCUGAGAGCACCUCCCA

GCUCCCCAGCAGGGCUCCCC**C**UCUCCACGUCCAUUGGCAGA

CCUUCCAUCCCGGGGGGAAG**C**AGGCUCCAGGCCUGAGAGCA

CCCCCAACCCCUUGGCUUGU**C**UGACACCUCUCUGUGCCCAC

UGCCCGCCCAGAUUCCUACC**C**GCCCGGAUUCCUGCCUGCCA

CUCCGGCAGGUGGGCCCCGC**C**UGCCCUCCACCUCCCGUGCU

CCCGGGCCCCCCAGACCCCU**C**GGCCUCUCUGAGUGUCCUGU

GGGGGUGGCUGGACAGAUGC**C**CAGGGUUGACCUGUGUCUGU

CCUCCAGGUGCUUCAUUCUC**C**UCCUAACGAUGAGGCUGGUG

CGCCCAGGGUCAGCCCCCGC**C**GUGCAACCGUCCCGGCUUCG

GGCUCCUGCCCCGGAGCGUC**C**AAGUGAGUGGGCUCCUGGCC

CGCCACCCAUCCAUUCCUGC**C**CAGACAUCUGCACUAGGCAG

AGCGACCACUGCAGGGGCCG**C**CUUGAGGUGCCCUGCCAGAG

GGUGGAGUCCAUUUGCUGAC**C**CCACAGCCUGCAUCCUGCCC

UGUGAGGCUGAGGGUGGAGU**C**CAUUUGCUGACCCCACAGCC

CACUGGCCACACUGGGUCUC**C**UCUGGCCACAAUCCGUCCGC

GGGCACCUUACGUCGACAGC**C**AUGAGCUCCACAACUGCUGC

UUCUCCUCCUAACGAUGAGG**C**UGGUGACCUCUGGCCUGCCC

GCAACAGCCUGGGGGCAGCA**C**ACACUGGCCUGGGGUCCCCG

AUCCGUCCCCAGUGGCCACA**C**UUGGUCCCCACUGGCCACAC

AGACGGUGUGCGGUAAGACG**C**UGCAGAGCAGAGGUGCCCGG

CAGGCUCCGUGUCCACAGGG**C**UGAAAAUGCUGACACAGCCC

AGGCGGGCAGGGGAUAGACU**C**CCUGCUGAGGGUCUGGGGAG

UCAUUGGAUUGAGGGCCCAC**C**CAGCUAGUCCACGAUGAUGU

UUGGCCCAGUCCCAACCGCA**C**CUGGGCAGGCCGACUGCAGG

UGCGGCCCCAUACAGCCUGC**C**ACCUGCAACUCUAGGUAAGU

GGUAAUCCCUACUCAGCUUC**C**ACACUCACCCUUGCAUUUCA

CUGCUGCUGGGCCUGACAAC**C**CAGUGAGCAUAGGGGAAGCC

GACCCCGCCGCCAGUAUCUC**C**AGUUGGAGGAGGCACACAGG

ACCUUCCCACACCAGCCCUC**C**AGCUCCAGCCCGUCGCCACC

GGGGCGCCCCCGCCCGCCCG**C**AUGCACGCACGCACGCAGCU

CCUGGAGAACUGCACGGUGG**C**CAGGUGCGUGGGUGACAACC

UCUCUGGGGACACCCAGGAC**C**CAACGGUGCAAUGUCAGGAG

GAGCCGGUGCCCACCAGGGG**C**CUGUGGGUUGGGCACAGGAG

AGACCUCACCUGCCCACCCA**C**CAAAGUGUACAAGCCAUGCG

CCCAGUGAGCAUAGGGGAAG**C**CUGGGGAGGGGAAUGAGUGG

CACCUGCCCCCAAGUGAGAC**C**CGAGGCACCUGCCCCCAGGU

GAAGGGGCUCCCCAGCAGGG**C**UCCCCCUCUCCACGUCCAUU

CUCACUUGGCCCCUCCCGGC**C**ACACUGGGUCCCCACUGGCC

AGACAGUGGCCUCCAUCCUC**C**CGCAGUGUGCAACACAACCA

GUGAGGCUGAGCCGGGAUAA**C**UGAGUGGGGGCAACUUCUUC

CAUUUGGACAGUUUUUCUCG**C**CCGGUGAGUGCAUGUGGAUA

GGCAUCUGCAGCAUGUGGGG**C**GGCUCCCACUAUUCCACCUU

UGGAGCUGGAAGUCUGAGAU**C**CAGGCGGGCAGGGGAUAGAC

AGCCCCCGAGGCACCUGCCC**C**CAAGUGAGACCCGAGGCACC

CCCCUGGACACAAUCCAUCC**C**CACUGGCCACACUGGGUCUC

CCGUGUCGUCCUGCUGGACC**C**AAAGCCUGUGGCCAACGUCA

GUGGGUGGAACCAGAGCCUU**C**UGUGAAGCCCCCCAUGGCUU

UGCCCUGAGGACCAGAUCCU**C**UUCAACGCACACAUGGGCAU

GGGGGCGGCCCCGGGCCCCC**C**AGACCCCUCGGCCUCUCUGA

CCACAGCUGCUGGGCAGACC**C**AGCCCUGAGUCACUUAUCCU

GUGGACAUUCCUGCCCUGGG**C**GUGAGCGUCACCUUCAAUGG

UCAAGAUGCUUCCCUUAAUC**C**CAUCUGCAAAGACACUUUCU

GGAGCUUUUGUCUCCUGGGU**C**CUAACAGCGGCUUCCAUCAC

CUGGCCACAAUCCAUCCCCA**C**UGGCCACACUCGGUCCCCAC

AGGUGGGCCCCGCCUGCCCU**C**CACCUCCCGUGCUGCGUGCA

GGGCUGCUGUUCCAAACCGC**C**ACAAGCUGGGGAGCUUAUAC

CUGCCCAUCCUGGGGACUCA**C**GUGGAUGACAGUGGAGGCCU

UGGUCCCCACUGGCCACACU**C**AGCCUCUGCCCUCUCCACUC

UCGUCCUGCUGGACCCAAAG**C**CUGUGGCCAACGUCACCUGC

CAGCCUCUGCCCUCUCCACU**C**CCUUCCCUGGAACCGCUGCU

GCCAACGUCACCUGCGUGAA**C**AAGCACCUGCCCAUCAAAGU

CACAGGGUGGGAGGAGCCGC**C**CAGGACCAUGAUGUGCUCUC

CCCUCGGCCUCUCUGAGUGU**C**CUGUGCGGUGAGUGGGGGCG

UCAGCCUCUACCUGGACAAC**C**ACUACUGCACGGCCUCUGCC

CCCAGGGGCUUCAGUGGCUC**C**CCAAGGCGGCAGUCACAGUG

GAGGUCCUUCCUGCCUCUCC**C**AGCUUUGGGGACUCCAGGUG

GGGGCCGGGCUCCUGGGUGG**C**CUCUUGCUGGGGGUGGGGGA

UUCAAGAUGCUUCCCUUAAU**C**CCAUCUGCAAAGACACUUUC

GUUUCAGCAAGAACGGCGUG**C**UUGUGUCUGUGCUGGGGACC

UGACAGCUGGGCUCACGGUG**C**CCUGGCCUGAGCUCCAGCCA

CCCAGGGCUGCUGUUCCAAA**C**CGCCACAAGCUGGGGAGCUU

CCCUGCUGGUCAUGUUUGUU**C**CCCACUGGCCUCACUUGGCC

GUAAGCUCCGCCACCUGUGG**C**GGGAUACGACCCUGGGCCCG

AGGGCAGGAGUCCAUCUGCA**C**CCAGGAGGAGGGCGACUGCU

CCAUCCCGGGGGGAAGCAGG**C**UCCAGGCCUGAGAGCACCUC

CUAUGCCAACCUGGGUCUGC**C**UGUCCUGGGAGCCAGUGGCU

CCUCCCAGCUCAGGGUUCCC**C**UGGAUUCCCCCAGAUCCUGU

CGCCCAUACUUAGCCCCGCC**C**ACCCCCACCCCAGCCCCACC

GUGGGGGCGGCCCCGGGCCC**C**CCAGACUCCUCGGCCUCUCU

ACACUGCUGUACCCUUUCCC**C**ACAUGCUAUGCCAACCUGGG

GAGUGCCACAACCUUGUGCC**C**CCGGGCCCAUUCUUCAACGC

UUCAUCUCCUUCCCUGCUCC**C**CACUGCCCAUCCUGGGGACU

CACUGGCCACAAUCAGUCCC**C**ACUGGCCACACUUGGUCCCC

GGGCCCCCCAGACCCCUCGG**C**CUCUCUGAGUGUCCUGUGCG

ACCUGCCCCCAAGUGAGACC**C**GAGGCACCUGCCCCCAGGUG

GGCCUGGGGUCCCCGCCUGC**C**CGCCCAGAUUCCUACCCGCC

UGCAUCCUGCCCAGAUGUGU**C**CAGCUGCAGGGUGUGUGCUG

GCCACUGUGUCCUGGCGUGA**C**CGCGGCAGGACCACUCGGCA

CACCUGGCCCCGCCACCGAG**C**CCCACCCAUCCCCGCCCAUA

AAUGCAGCCAGCUGGAGACU**C**CAGGCCCCCAGGGAAGAUCU

GUGUGACGAGGGUUCAGUGU**C**GGUGCAGUGCAAGCCCCUGC

GUGCCCUCAGUGCCACCCUC**C**CACCCCUUGCAGGUUGGUGC

UCUCCCAGAACUCUGGCUUA**C**CCAUCUCUGGGAGUGGCUUA

CCACAGACCUCACCUGCCCA**C**CCACCAAAGUGUACAAGCCA

AUGCCCCGGGGCUCUCUGAG**C**CCCACUCCUUGUCUUGACAU

CUCCCCUGGACACAAUCCAU**C**CCCACUGGCCACACUGGGUC

CCCGCCUGCCCGCCCAGAUU**C**CUACCCGCCCGGAUUCCUGC

CUUGUGUCUGUGCUGGGGAC**C**ACCACCAUGCGUGUGGACAU

GGGGAAAAGCACGCCUGCGA**C**UUACUCUGGGAACAAGUGGU

AGUGAGACCCGAGGCACCUG**C**CCCCAGGUGAGACCUGAGUC

CAGGCCAGGGCCCGGCUGAG**C**CGAGCCACAUGGCACAGAGC

CGUGGGACCCGAUGGGUUUC**C**UAAAUUUGUGAGUGGCUCCA

GAGCUCCACAACUGCUGCCU**C**UGAGAGGUCCCUUCAGGGGC

GCCUCCAGGUGCUUCAUUCU**C**CUCCUAACGAUGAGGCUGGU

UGGGCCCCGACUGGCACACC**C**CCCACUGCCAGCCCCGCAGC

GGCGGGAUACGACCCUGGGC**C**CGACCCAAGCACACACAGGG

CCGCCCAUACUUAGCCCCGC**C**CACCCCCACCCCAGCCCCAC

UGCCACCCUCCCACCCCUUG**C**AGGUUGGUGCAACCUUCCCA

GCUUAUACAACAGAAACCCA**C**UCUCCGUCCUGGAGCUGGAA

GCCUCUCUGAGUGUCCUGUG**C**GGUGAGUGGGGGCGGCCCCG

UUGGCCCAGGGCUGCUGUUC**C**AAACCGCCACAAGCUGGGGA

GUGGCCUCCCCUCUCUGUGU**C**UGUGUCUCUUCUGUCUCCCG

AGCAUGUGGGGCGGCUCCCA**C**UAUUCCACCUUUGACGGCAC

CGCCCCCAGCCCCGCCCACC**C**UGAGCCCCGCACUCCACCCA

AGUGCAUGUGGAUAACACUG**C**UGUACCCUUUCCCCACAUGC

GAGUCCAUUUGCUGACCCCA**C**AGCCUGCAUCCUGCCCAGAU

CCGUGCUGCGUGCACACGGU**C**UGGGUUGGCUGGAGGCACAG

UCCCGGCUUCGUAACCGUGA**C**CAGGCCCCGGGCCGAGAACC

GCCCAGGACUCCUUGGAAAA**C**AUCCCCUGCUGCUCCCAGAU

GAGGGGAUGGCGGAGGGCUG**C**UUCUGCCCUGAGGACCAGAU

GGUGUGUGCUGGAGGCCCUG**C**CCCUGCCUGGGAGUCCUUGU

CCCUGGGGCGCCCCCGCCCG**C**CCGCAUGCACGCACGCACGC

GGUGGAGCAGAGUGCACCGU**C**GGCUAGGCUGGCAGAAUGGG

UUCUGGGGCCAGGAGAAGCU**C**AGGAUGGAAGCGGGAGCCCA

GUCCUCGGGGAAAAGCACGC**C**UGCGACUUACUCUGGGAACA

CACGGCCUCUGCCACUGCCG**C**UGCCGCCCGCUGCCCCCGCG

GGUCUCCUCUGGCCACAAUC**C**GUCCGCACUGGCCACAAUCA

ACCUGCCCCCAGGUGAGACC**C**GAGGCACCUGCCCCCAGGUG

AUGAGAGAGAUCCAUGCACG**C**UUUGGGAAUCUCAGCCUCUA

GCCUCUGCCCUCUCCACUCC**C**UUCCCUGGAACCGCUGCUCC

CUCCCCGAGCCCACCUGGCA**C**UGCCUCCCAGCUCAGGGUUC

UGCCCCCGCGCCCUCAGCAU**C**CACUACAAGUCCAUGGAUAU

CACUGGCCACAAUCCAUCCC**C**ACUGGCCACACUCGGUCCCC

ACCCGCCCCCAGGUGAGCCC**C**CGAGGCACCUGCCCCCAGGU

CUGCACGGCCUCUGCCACUG**C**CGCUGCCGCCCGCUGCCCCC

AACACUGCUGUACCCUUUCC**C**CACAUGCUAUGCCAACCUGG

UCCUGCCCUGAGGGCCGAUC**C**GCACAGGGGCCCUGGACACG

CCCAUUCUACACACUGGACC**C**AUUUUUAUAGACGAGGCAGC

UGGCCCAGGGACGUGGGAAG**C**AGCGGGGAGGUGGCCAAGCA

AUCCUGUGAUGGUCCCUCCC**C**UGAGCCCUGCCUCCCACCAC

AGCCACAUGGCACAGAGCUC**C**CCGCUGCGAACCCAGGUCCA

GAAGUCAUCUACAAUAAGAC**C**GACCGAGCCGGCUGCCAUUU

CCCGAGCCCACCUGGCACUG**C**CUCCCAGCUCAGGGUUCCCC

CGAUCUCUGCCUCUGCCUCU**C**UGUGGCCUCCCCUCUCUGUG

CAAUCAGUCCCCACUGGCCA**C**ACUUGGUCCCCACUGGCCAC

GCCACCUUCCCACACCAGCC**C**UCCAGCUCCAGCCCGUCGCC

CUAGGUAAGUACAGGGAUGG**C**UGGUGCCUUCCCUGCCACCC

UCCCAGAACUCUGGCUUACC**C**AUCUCUGGGAGUGGCUUAUC

CUGGGGAGGGUGGGUGGAAC**C**AGAGCCUUCUGUGAAGCCCC

CCCACAGCUGCUGGGCAGAC**C**CAGCCCUGAGUCACUUAUCC

CCGCCCAGAUUCCUACCCGC**C**CGGAUUCCUGCCUGCCAGAU

GGGGACAGAGCCGGUGCCCA**C**CAGGGGCCUGUGGGUUGGGC

CACUCGGUCCCCACUGGUCA**C**AAUCCGUCCCCAGUGGCCAC

CUGGCCACAAUCCGUCCGCA**C**UGGCCACAAUCAGUCCCCAC

CUCAGCACCCUCCGUGAUGC**C**AUGCUGUUUUCUUUCCAGCC

CCCCACAGCCUGCAUCCUGC**C**CAGAUGUGUCCAGCUGCAGG

CUAACGAUGAGGCUGGUGAC**C**UCUGGCCUGCCCAGGAGUGG

CUGCCGCUGCCGCCCGCUGC**C**CCCGCGCCCUCAGCAUCCAC

CUCUGGGUCAGGAGGGCCUA**C**GCCAGCUCCAGGAAGGAGGG

CGCUGACCUCUGCCUUUGCU**C**UCCCAGAACUCUGGCUUACC

GCCGCCUAAGGCCGAGCGCA**C**CCUGUGGCCUAAAUGCAGCC

UAAUCCCUACUCAGCUUCCA**C**ACUCACCCUUGCAUUUCAGC

CCUGCAACUCUAGGUAAGUA**C**AGGGAUGGCUGGUGCCUUCC

UGGGUUGGCUGGAGGCACAG**C**CACAGUCCAGCUCCCGAGGC

CCAUGCUGUUUUCUUUCCAG**C**CUGCGUGGGACCCGAUGGGU

GGACUCCAGGUGUCCUUUGG**C**UGUGGCUGCAUCCCUCCGAU

UAGCCCCGCCCACCCCCACC**C**CAGCCCCACCCGUCCCCGCC

GGAGAAGCUCAGGAUGGAAG**C**GGGAGCCCAGAGGAGCUUUU

UCCCCAGGAGGCCGCACCCA**C**CAGGGAGGCCCCGCCCACAG

UUCCUGCCCCCAUGGGGUCU**C**UGCCCACCCAGAUUCCUGCC

AGGCCCGGCUGCCCUACAGC**C**UCUUCCACAACAACACCGAG

CUCCCGGCCACACUGGGUCC**C**CACUGGCCACACUGGGUCUC

CCGCGUCCAGACUCCACCCU**C**GGUCCUGGAGGGCCAUGGGA

GAGGGAAGGAGCAUCCCCAU**C**CCACAGGGCAGCUGUGGGGC

CCACCCCUGCCUGGGAAGCC**C**CACCCCUGCCUGGGAGGCCC

UCCAGGUGCUUCAUUCUCCU**C**CUAACGAUGAGGCUGGUGAC

CUUCUGCGAGGGCUCCUGCC**C**CGGAGCGUCCAAGUGAGUGG

CGCAGCGAUGGCCCAGUGCC**C**AAGACAGCUCCCAGGGGGCA

UUGGGAGAUCGCUGGCAUCC**C**UUCAGGAAACCAUCAUGCAC

CUUCCAGGGCGCCUGUCCCA**C**CUCCCCACCGCCAGUGUCCU

AUGGACGAGGCUUCCAUGCA**C**UGACAGCUGGGCUCACGGUG

GUGAAAGGCUCCCCAGAUUC**C**AGCCCCGCGGUGACGCCCCC

UGCCCCCUGUCUUUGGCCCC**C**ACCAGGGGAGCCUCAGGAAA

GAGGUCAACAUCACCUUCUG**C**GAGGGCUCCUGCCCCGGAGC

ACCUCUUACACCUUCCGGGG**C**AACUGCACCUAUGUCCUCAU

UGCGGUGAGUGGGGGCGGCC**C**CGGGCCCCCCAGACCCCUCG

CCCUGUUGCCCCACCAGUGC**C**CUCAGUGCCACCCUCCCACC

CCUUCCCUGCUCCCCACUGC**C**CAUCCUGGGGACUCACGUGG

AGAAAGGAUGGCUGCUGGGC**C**CCGACUGGCACACCCCCCAC

CACCUGUGGCGGGAUACGAC**C**CUGGGCCCGACCCAAGCACA

UUCAAGUGAUUCUCCUGCCU**C**AGCCUCCUGAGUAGCUGGAA

UUUCCUCUGUCUUUUUGGAU**C**UCAUAGUGCAGUGUAGUGUG

GGCAUUACAGGUGUGAGCCA**C**UGCAUCUGGCCUACCAGUAC

GCCCAGGAGUUCAAGACCAG**C**CUCGGCAAAAUAGUGAGACC

CCCAGGAGUUCAAGACCAGC**C**UCGGCAAAAUAGUGAGACCC

CUUAAAUAUAAUUUGGGGGU**C**CUCUUUAUUGUGCAAAAGAA

UUUGGAAAGUUAGUCUUUAA**C**GUAGAUCUUUUCCUUUGAUU

AUGUGAGCCAUUGCGCCCGG**C**CUGUUUUUUUUUUUUUUUUU

GCAGGAGAAUGGCGUGAACC**C**GGGAGGCGGAGUUUGCAGUC

GAGCUGUGAGUGCACUACUG**C**ACUUCCACCUGGGUGACAGA

GCAGUGGCAUGAUCUUGGCU**C**ACUGCAGCCUCCGCCUCCCG

CAUUUUUUAACAUUUAAAAC**C**GUACCACCCUCUGGAACUUG

UACUUAGGAAUCGAGAAUUU**C**UAGUUCAGAACUGCAAAAAG

AAUGGCGUGAACCCGGGAGG**C**GGAGUUUGCAGUCAGCCGAA

CUCCGCCUCCCAGGUUCAAG**C**AGUUCUCUGCCUCAGCCUCC

GUCAUAGUUUCAUUUUUUAA**C**AUUUAAAACCGUACCACCCU

UUCACAAAGUAAUGCUUCAU**C**AUCACUAGAAGUGGUCACUG

UCACGCCUGUAAUCCCAGCA**C**UUUGGGAGGCCGAGGCUGGC

GUGCACAUCCAUGAGAUUUU**C**AAAGAAAUAUUUGGAUAUAG

AGCCCGAGAUUGCGCCACUG**C**ACUCCAGCCUGGGCGACAGA

GUGAGCCCGAGAUUGCGCCA**C**UGCACUCCAGCCUGGGCGAC

AUUCCUGACCUGGUGAUCUA**C**CUGCUUCGGCCUCCCAAAGU

CUUAAGUAUUUGGUGGACAG**C**CAUGUUGAAGGAGAUUUUUC

AACUCCUGAACCCAAGUGAU**C**CACCCACCUUGGCCUCCCAA

UAACCUCCAGACCUGGUGAU**C**UGCCCGCCUCAGCUUCCCGA

ACAGGCGUGAGCCACCGUAC**C**CAGCCUAGUUUUGCAUGUCU

UGGUCUCCAUCUCCUGACCU**C**GUGAUCCGCCCACCUAGGCC

ACGAGGUCAGGAGAUCGAGA**C**CAUCCUGGCUAACACGGUGA

AGAGAUUUGCUAAUUUACAG**C**AAAUUUGCAGAUAGCAUCUU

ACAGAUGUGAGCCAUUGCGC**C**CGGCCUGUUUUUUUUUUUUU

UCCCAGGUUCAAGUGAUUCU**C**CUGCCUCAGCCUCCUGAGUA

AUUACAGGUGUGAGCCACUG**C**AUCUGGCCUACCAGUACUAG

UCGGCUCACUGCAACCUCCG**C**CUCCCAGGUUCAAGCAGUUC

UGUUGAUUUGUUGUUGAUAG**C**AUGUAUUUCUUGACCCUUAG

GCGGGCACCUAUAGUCCCAG**C**UACUCGGGAGGCUGAGGCAG

CGGCCUGGCGCAGUGGCUCA**C**GUUUGUAAGCCCAGCACUUU

GUAGCUGGGACUACAGGCAC**C**UACCACCGCGCCCAGCUAUU

CGGCUCACUGCAACCUCCGC**C**UCCCAGGUUCAAGCAGUUCU

CGAGGUCAGGAGAUCGAGAC**C**AUCCUGGCUAACAUGGUGAA

UGCCUCAGCCUCCCAAAGUG**C**UGGGAUUACAGGCGUGAGCC

UCCCGGGUUCAAGUGAUUCU**C**CUGCCUCAGCCUCCCAAGUA

AACAUUUAAAACCGUACCAC**C**CUCUGGAACUUGCCAUCUGU

CUAUGCCAGGUCUCAAACUC**C**UGAACCCAAGUGAUCCACCC

CAAUACUUACUGAACCCUUU**C**CCCCUUUAUUUUGCCAGUCU

GCAGGAGAAUGGCGUGAACC**C**GAGAGGCGGAGCUUGCAGUG

GCCUCAGGUGAUCUGUCAAC**C**UCGGCCUCCCAAAGUGUUGG

AUCUUGGCUCACUGUAACCU**C**CACCUCCCGGGUUCAACCGG

CGUGAGCCACCGUACCCAGC**C**UAGUUUUGCAUGUCUCUCUA

CUGUGUAGCUUUGAAGUCAU**C**CUGAGAUAACUGCUCUGAUC

ACUCAAGAAGAGAAGACUUG**C**AACAUUUUAAUUAUACUCUU

GUGCAGUGGUGUGAUCUCCG**C**UCAUUGCACGCUCCGCUUCC

AGCUACUCGGGAGGCUGAGG**C**AGGAGAAUGGCGUGAACCCG

CUUUUCACAUGACUCCUCUU**C**CUGGGCAUAGGAACUUAAAG

CGGUUCUCUUGCUUCAGCCU**C**CAGAGUAGCUGGGAUUACAG

GUGGGCUGUCUUAAGGAAGC**C**CUUAUAUUGUAGUUGGCUAA

AAUAAGUAAUUUCUUCUUUU**C**ACAUGACUCCUCUUCCUGGG

CUGACCUCGUGAUCCGCCCA**C**CUAGGCCUCCCAAAGUGCUG

AAUGGCGUGAACCCGAGAGG**C**GGAGCUUGCAGUGAGCUGAG

AUGUUUAUUUAAAGCACUGU**C**AUUGAAAUAUUUCAUGAACA

AAAAUAGUGAGACCCCAUCU**C**UUAAAAAACAAAAUUAGGGC

AGUCAGCCGAAAUCGCACCA**C**UGCACUCCAGCCCUGGGUGA

GCUCACUGUAACCUCCACCU**C**CCGGGUUCAACCGGUUCUCU

UUUGAAAAUGUUCUUUUCUU**C**CCUUAUGGACAAGAUUAGAA

GUGGGCGCCUGUAGUCCCAG**C**UACUCGGGAGGCUGAGGCAG

CUUUUUCUCCCUCCCCAUCU**C**CUUUCCUUCUUUUAUGACAA

UUUUAACAUUUAAAACCGUA**C**CACCCUCUGGAACUUGCCAU

ACAGCAUUUAACCAGAUUAU**C**AUCAUACUUUUUAAAUUAUG

GAUGGUCUUAACCUCCAGAC**C**UGGUGAUCUGCCCGCCUCAG

CUUUGGGAGGCCGAGGCUGG**C**GGAUCACGAGGUCAGGAGAU

GCAAUGGUGCGAUCUCGGCU**C**AGUGCAGCCUCCACCUCCCA

CUGGCUAACAUGGUGAAACC**C**UGUCUCUACUAAAAAUACAA

UGGAGAAGAAGGUGCACAUC**C**AUGAGAUUUUCAAAGAAAUA

UAAAAUAUUAUGACCUGUGA**C**AUCAACAUGGAUUAACAAGU

AAAUUAGCUGAGUGUGGUGG**C**GGGCACCUGUAGUCCCAGCU

UAGCUGAGUGUGGUGGCGGG**C**ACCUGUAGUCCCAGCUACUC

UACAGAUGUGAGCCAUUGCG**C**CCGGCCUGUUUUUUUUUUUU

UACAGGCGUGAGCCACUGUG**C**CCAGCCUUUUCUUUCCUAAU

CUGUUGCCCAGGCUGGAGUG**C**AGUGGUGGGAUCUCGGCUCA

CAAGUAUCUGGCUUCUUGGU**C**AAGGGUUAGGAAGUUACACU

CGCUCCGCUUCCCAGGUUCA**C**GCCAUUCUCCUGCCUCAGCC

AGUAGCUGGGACUACAGGCA**C**CUACCACCGCGCCCAGCUAU

UUCGAGAAGCAAAAUUUGUA**C**AUGUAUGGCUUACAGAAAUU

CAGAACUGCAAAAAGAUAAC**C**UUGUGUAAUUCUUUGUCAUU

GUAGCUGAGACUACAGGCAC**C**CACCACCAUGCCCGGCUAAU

UUCUAAAAAUACUCAUUUUU**C**UUUUUCUUUUUUUCUUUUUU

CUUCCACCUGGGUGACAGAG**C**AAGAGUCUGUCAAAAAAAAA

UAACGUUUUAAAAUGAAAAA**C**AGUUUUGCAAGUCUCUCUUU

CUCCACCUCCCGGGUUCAAC**C**GGUUCUCUUGCUUCAGCCUC

UUUGUCCUUUUUCUCCCUCC**C**CAUCUCCUUUCCUUCUUUUA

GCAGGAGGAUAGUUUGACCG**C**AGGAGGUGGAAGCUGCAGUG

UCCUUUUGUCCUUUUUCUCC**C**UCCCCAUCUCCUUUCCUUCU

UAAACUUUGUCAUGAGGUUG**C**UAGUUGUUUCACUGUCAUUU

AAUGAGUUUCUUGUAGAAGA**C**CUGGAUUAAACAGGGUAAUA

GGAACUGCAGGUGCACGCCA**C**CACGCCCGGCUAAUUUUUUG

UUACCCCAUCCUCCCAGGUC**C**UUUUGUCCUUUUUCUCCCUC

GGUUAGGAAGUUACACUGUU**C**UUUAGUAACAGUGAGCAAUG

AGGCCGAGGCUGGCGGAUCA**C**GAGGUCAGGAGAUCGAGACC

AGUCCCAGCUACUCGGGAGG**C**UGAGGCAGGAGAAUAGCGUG

CUUGCAGUGAGCUGAGAUCG**C**ACCACUGCACUCCAGCCUGG

AUCUUUCAUGGAUUUGAUAG**C**CUGCAUAAUAAGUAAUUUCU

AGCUGGCAUGGUGGUAUGUG**C**CUGUAGCCCCAGCUACUUGG

UUUUGUCCUUUUUCUCCCUC**C**CCAUCUCCUUUCCUUCUUUU

AACCCAAGUGAUCCACCCAC**C**UUGGCCUCCCAAAGUGCUGG

CAGCCUCCACCUCCCAGGUU**C**AAGUGAUUCUCCUGCCUCAG

UCGUGCCUGUAGUCCCAGCA**C**UUUGGGAGGCCAAGGUGGGC

CACGCUCCGCUUCCCAGGUU**C**ACGCCAUUCUCCUGCCUCAG

CCUAGACUGGAGUGCAGUGG**C**AUGAUCUUGGCUCACUGCAG

AUCGCACCACUGCACUCCAG**C**CCUGGGUGACAGAGCGAGAC

GUGCAGUGGUGGGAUCUCGG**C**UCACUGCAACCUCCGCCUCC

GGCUGGUCUCAAUCUCCAGA**C**CUCAGAUGAUCCACCCGCCU

GAUAAUUGCAGUUUCUCUCU**C**ACUUUUUAUUUUCCUCUGUC

GACGGAGUCUCGCUCUGUUG**C**CCAGGCUGGAGUGCAGUGGU

GCUAACCAACAGGCCUGCGC**C**AUCACACCCAGCUAAUUUUU

UUUUUUUUGAGACGGAGUCU**C**GCUCUGUUGCCCAGGCUGGA

AGUUUCACAAAGUAAUGCUU**C**AUCAUCACUAGAAGUGGUCA

AGGGAGUUUACCCCAUCCUC**C**CAGGUCCUUUUGUCCUUUUU

UAAGUAAUUUCUUCUUUUCA**C**AUGACUCCUCUUCCUGGGCA

CUUCCCAGGUUCACGCCAUU**C**UCCUGCCUCAGCCUCCCGAG

GAGAUAUAAUUGAAUUCACC**C**UUUAAAGUAUAGUUUUUUCU

CCUCGGCAAAAUAGUGAGAC**C**CCAUCUCUUAAAAAACAAAA

GAGACUACAGGCACCCACCA**C**CAUGCCCGGCUAAUUUUUUG

GCUAAUUUACAGCAAAUUUG**C**AGAUAGCAUCUUAAAUAUAA

AUGAUCUUGGCUCACUGCAG**C**CUCCGCCUCCCGGGUUCAAG

CAAAGUGCUGGGAUUACAGG**C**GUGAGCCACCGUACCCAGCC

UCGUGAUCCGCCCACCUAGG**C**CUCCCAAAGUGCUGGGUUUA

GUCCCAGCACUUUGGGAGGC**C**AAGGUGGGCAGGUUACUUGA

UGAAAAAUAAUUAAGCAGAG**C**UAAUAAAAAUGUUGCACAAA

CCUCCCGGGUUCAAGUGAUU**C**UCCUGCCUCAGCCUCCCAAG

ACCUCAGAUGAUCCACCCGC**C**UUGGCCUCCCAAAGUGCUGG

CAGUUUUAUAUAUAUGUUCU**C**AUCCAGCUCGAUUUGUGUUU

AGAAAUAAGGGCAGAGUUUG**C**CAGAUAAUUGCAGUUUCUCU

UCUUUGUCAUUUUUUUUUCU**C**AUUAUUUGGAGACUUUUGAC

UCUCCUGACCUCGUGAUCCG**C**CCACCUAGGCCUCCCAAAGU

CAGGUUCAAGCAGUUCUCUG**C**CUCAGCCUCCCAAGCAGCUG

AGGCUGAGGUGGGCGGAUCA**C**GAGGUCAGGAGAUCGAGACC

AGGCCAAGGUGGGCAGGUUA**C**UUGAGCCCAGGAGUUCAAGA

UGAAAAACAGUUUUGCAAGU**C**UCUCUUUCUUUUUUUUUUUU

UGGGGAGUUAACAAUCAUUU**C**GAGAAGCAAAAUUUGUACAU

ACAGAGCCUUUGCACUGUUU**C**CUAGACUGGAGUGCAGUGGC

AAUACUUACUGAACCCUUUC**C**CCCUUUAUUUUGCCAGUCUU

UGGUAUCCUUGUUUAUUGCC**C**AGAUAAGUUUAAUCUUUUUU

CGGUGGCUCACGCCUGUAAU**C**CCAGCACUUUGGGAGGCCGA

GUGACAUCAACAUGGAUUAA**C**AAGUAUCUGGCUUCUUGGUC

GCCGGGCUCAGUGGCUCAUG**C**CUAUAAUCCCAGCACUUUGG

GAGAUCGCACCACUGCACUC**C**AGCCUGGGAGACAGAGCAUG

UUGUGUGUUGUGUUUCUUCU**C**UUUUUGGAGUCUGAUUUUUA

GGGUUUACAGGCGUGAGCCA**C**CGUGCCCGGCCUAAAAAUAC

CCCUUUCCCCCUUUAUUUUG**C**CAGUCUUUUCUACGUAAUAG

UCCUGACCUCGUGAUCCGCC**C**ACCUAGGCCUCCCAAAGUGC

CCAGACCUCAGAUGAUCCAC**C**CGCCUUGGCCUCCCAAAGUG

GUUUUGCCAUGUUGGCCAGG**C**UGGUCUGGAACUCCUGACCU

UAUGAGUGCACUGUUAUGCC**C**AGAAGAGUAUAGGAGGUGCU

ACAGGGUUUUGCCAUGUUGG**C**CAGGCUGGUCUGGAACUCCU

UUUUUUUUUAAACCCAGUUG**C**AUUGUAUUUAUGUGAUAAAC

GCAGUGGCGCAAUACUCCGC**C**UCCCGAGUUCAUGUCAUUCU

GCAAAAUAGUGAGACCCCAU**C**UCUUAAAAAACAAAAUUAGG

AAUUCUAAACAGCAUUUAAC**C**AGAUUAUCAUCAUACUUUUU

CUUGUUAAUGUUGGGGAACU**C**ACUUUUCUAGUUAAUUCAGU

GCAGUUCUCUGCCUCAGCCU**C**CCAAGCAGCUGGGAUUACAG

AAUGACUGUGACCUCAGGUA**C**AUGCAUAUAGAUGUUAUAAA

AGGCGGGCAGAUCAUGAGGU**C**AGGAGAUCCAGAGCAUCCUG

UGGGAAAAAGAGAAAUAUUA**C**AAAAAAUGUGCUUUCAGUGA

AAAGUAAUGCUUCAUCAUCA**C**UAGAAGUGGUCACUGGGAGG

UGUGCCCAGCCUUUUCUUUC**C**UAAUCUGAUUUACUCAAUGA

AUAGGCUUUCCUUCCUAAUA**C**UGGAUUGCAGUGACACUUGC

CUGCUCUGAUCUUAAAUGAU**C**CUCAGAUACUUGAAGAGUCC

AUUCUUUGUCAUUUUUUUUU**C**UCAUUAUUUGGAGACUUUUG

AGGGUUUCACUAUGCCAGGU**C**UCAAACUCCUGAACCCAAGU

AGCCUCCCAAGUAGGUGGGA**C**UACAGGCAUCUGCCACCAUG

AUCUGUCAACCUCGGCCUCC**C**AAAGUGUUGGGAUUACAGGU

CUUUUGUCCUUUUUCUCCCU**C**CCCAUCUCCUUUCCUUCUUU

GGUUUACAGGCGUGAGCCAC**C**GUGCCCGGCCUAAAAAUACU

GCUCACUGCAACCUCCGCCU**C**CCAGGUUCAAGCAGUUCUCU

UGGUGGCGGGCACCUGUAGU**C**CCAGCUACUCGGGAGGCUGA

CUCCCAAAGUGCUGGCAUUA**C**AGGUGUGAGCCACUGCAUCU

CCCAGGUUCACGCCAUUCUC**C**UGCCUCAGCCUCCCGAGUAG

GGAGAUCGAGACCAUCCUGG**C**UAACACGGUGAAACCCCGUC

UACAGGCACCUGCCACUAUG**C**UCACCUAAUUUUGGAAUUUU

CUGAGACUACAGGCACCCAC**C**ACCAUGCCCGGCUAAUUUUU

GGUGAUCUGUCAACCUCGGC**C**UCCCAAAGUGUUGGGAUUAC

AACCGUACCACCCUCUGGAA**C**UUGCCAUCUGUUAUGAGUGC

GGGAUUACAGGCACACGCCA**C**CAUGCCCUGCUAAUUUUUGU

AGUGCAGUGUAGUGUGUGAU**C**UCAUCUCAGUGAAAUAAAAU

UUUGUUUAUGUAGGUUAUAU**C**UACCAAUAUUUGUCAUAUUA

UGUUGAGAUAUAAUUGAAUU**C**ACCCUUUAAAGUAUAGUUUU

CUACGUAAUAGGCUUUCCUU**C**CUAAUACUGGAUUGCAGUGA

UGGUCUCAAUCUCCAGACCU**C**AGAUGAUCCACCCGCCUUGG

GGAUCACGAGGUCAGGAGAU**C**GAGACCAUCCUGGCUAACAU

CAGGCGUGAGCCACUGUGCC**C**AGCCUUUUCUUUCCUAAUCU

CAGAGUUUGCCAGAUAAUUG**C**AGUUUCUCUCUCACUUUUUA

CAGGAGUUCAAGACCAGCCU**C**GGCAAAAUAGUGAGACCCCA

CUCUUACCGGUGGCUGGUAG**C**UUAUGUACUUUCAGAGAAUG

ACGCCAUUCUCCUGCCUCAG**C**CUCCCGAGUAGCUGGGACUA

GAUCUGUCAACCUCGGCCUC**C**CAAAGUGUUGGGAUUACAGG

UAAUGUUAAUCAGUGGAGGU**C**AUAGUUUCAUUUUUUAACAU

CUCAGGUGAUCUGUCAACCU**C**GGCCUCCCAAAGUGUUGGGA

ACAGGCACACGCCACCAUGC**C**CUGCUAAUUUUUGUAUUUUU

CUCCAGCCUGGGAGACAGAG**C**AUGACUCCGUCUCAGAAAAA

UAGGUUGUAUCUUGAUUUGC**C**GCAUGAUAAAAGCCAUUAUU

GGGUCCCCCUCUCGUUAUCA**C**UGGAUCCUCAUCCUCUGAGG

GGGAGUUGCUCUGCAGCCCA**C**GCCGGGGGUUCUCCUGGGAC

ACGGCUGGCACCUCCCUUUC**C**AGAUGGGGAAACUGAGGCAG

CACUCCCUCCUCGCCCGCUC**C**AAGGAAGCACCCGCCGCCUC

CAGACCCCCAGCUCCCACCU**C**CUCCCAGACCCCCAGCUCAC

GCUCACACCUCCUCCCAGAC**C**CCUAGCUCACACCUCCUCCG

CCCUGCCGCUUCCGGCCCCG**C**AACACAGGCAGUUCGCACCG

CCUGGCCCCGCUGAGGUUCA**C**GCCGAGGUCCCCCCCGAAGU

CCUCCCUGAGACCCCCAGCU**C**ACACCUCCUCCCUGAGACCC

GGUUCCCCCCGAGGUUCCCG**C**CAGGGUCCCCGCCAAGGUUC

ACUUCAUGGAACUCUCCAGA**C**AUCCUGCUCCCUGCCCCUAA

CAGAGACACGCAGGUGGACA**C**ACACACACAUGCACACGCUG

CAGGGAAGUGGCCCUGCGGC**C**AUGACUCAUCUCAGCGCUCA

CUCACACCUGGUGAGACACU**C**ACCUGUGAGGCCCCGGGCCU

CUCCACCGAGCCCCAGUGCG**C**CAACUGCCCCAUCUGCUUGG

GCUUAGGGGACACACUGUAG**C**UGGGACGGGGCUGUCACUCC

CAGAUCCUGAGCUGGUAGUG**C**CAGGAAGGUCCCGGUCCCGG

CCGCCCCAGCACAGUGAACC**C**AGGACAACGUCGGGGCUGCC

GGGUCUGUCCCCUCCUCCAC**C**GAGCCCCAGUGCGCCAACUG

UCGUUUUAAAAGAAUUGUGU**C**UUUAACGGCCUGUCCUCAAG

GCUUCUGGAAGCUUGUGCCU**C**CAGCCCAGCCCUGCAUGGAG

GCUCCCAUCUCCUCCCAGAC**C**CCCAGCUCCCACCUCCUCCC

CGUCCUCCCCAGACCCCCAG**C**UCACACCUCCUCCUCCUGAC

CUGGUGAUUAGGAGGCCCCA**C**UCCCUCCUCGCCCGCUCCAA

CUCCUCCCAGACCCCCAGCU**C**ACACCUCCUCCCAGACCCCC

CUCCUCCCAGACCCCCAGCU**C**CCACCUCCCAGACCCCCAGC

AGCUCACACCUCCUCCGAGA**C**CCCCAGCUCACACGUCCUCC

CACCCUGGGAUCACUUGGGC**C**UAGGAUUUUGAGGCCAGCCU

ACCUCCUGCCAGACCCCCAG**C**UCACACCUCCUCCCAGACCC

CCCAGGCUGCUCAUCGGGGG**C**AGGAGGAGCCUGCGGCUGGG

CGAGGUCCCCACCGAGGUCC**C**CGCCGAGGUUCCCCCCGAGG

GUCCCCAAGCUGGGGUGCCC**C**UCACCCCACACCAAGGAGGG

GGAAGCUUGUGCCUCCAGCC**C**AGCCCUGCAUGGAGAGAAGG

GAGCCAAGAUGGCAUCAUUG**C**ACUCCAGCCUGGUGAACAAG

CAGCCUGGGUAUGCGUGACC**C**CAAAACAGCUGGUUAAUUCC

AGGGGGCUCAGCCUCUGCCU**C**CUGACCUGGGGAAGCCAGCA

UGUCCCCUCCUCCACCGAGC**C**CCAGUGCGCCAACUGCCCCA

UCCCAGACCCCCAGCUCCCA**C**CUCCCAGACCCCCAGCUCCC

CUGGUAGUGCCAGGAAGGUC**C**CGGUCCCGGUCAGGCCUGGG

CAGUUCCCUCAAUAGCUGCC**C**CGCUCGGACCCUGGCAUUGU

CCUGAGACCCCCAGCUCACA**C**CUCCUCCCCGAGACCCCCAG

CCUAGGAUUUUGAGGCCAGC**C**UGGCAACAUAGGCAGACCCC

UGGACACACACACACAUGCA**C**ACGCUGACUGCCCUGGCUCC

CCAGGUUUCCCCACCACGGG**C**UGCUGAAGCACCUACGGCUG

AGACCCCCAGCUCCCACCUU**C**CCCUGACACCUCCCAAGCUC

CCAAAACAGCUGGUUAAUUC**C**CUGAAAAGAAUUGAGUGCCA

GCACCAUCCCUGCUGCGGAA**C**GAGAGGCAGGCAGGUGUCCC

UCACACCUCCUCCUCCUGAC**C**CCCAGCUCCCAUCUCCUCCC

AUCCCCCCAGUAGGGUCCUG**C**CCACACUGGCUCCACCCUGA

GGACGGGAUCCGCCUGGAGA**C**UGCCAGCCCUCCCGUGCCGC

ACACCUCCUCCCUGAGACCC**C**CAGCUCACACCUCCUCCCUG

GAGAAGGAACCGGCCCACCC**C**GGCUGCAUCCCUGGGGCCGG

CCAGCGCUCCCGUGGGGCCC**C**ACCUGCCAUCCCCCCAGUAG

GGGUACACCGCCCGGGGGAG**C**UUGGUGCUGUGAAGGAGGGG

AAGGUUCGGCUCUGAGGUUC**C**CCCCAGAGGAUCCCCCCCGA

GUCCCCCCCGAAGUUCCCCC**C**UGAGGGCCCCCCAAGGUUCG

CUCGGUCAGAAUCUACCCUG**C**CGCUUCCGGCCCCGCAACAC

ACGGCAGCAGCAGAGGCUGA**C**GCUCCCACCACCCUGCCUUC

UUCCCUUGAGACUCUCAGCU**C**CCACCUCCUCCCCAGACCCC

GGCUGCUCUUUCAGGGGCGC**C**CCACGUCUGCUUGACCCCGU

CAAGUUGCCACAAGCCUGGC**C**CCGCUGAGGUUCACGCCGAG

GACCCCGUCCUCAGGGGCGC**C**AGGCAUUGAAGCAGCCUCGG

UCACACCUCCUCCCAGACCC**C**CAGCUCAUACCUCAUCCCAG

AUGUAGGCACAGGUGGCUGU**C**AGGGAGGGCUUCUUGGAGGA

GGAAGAGGGCAAGGUGGACC**C**CAGGUAGGGCUCCCCCAGCC

AGGUUCCCCCCGAGGUUCCC**C**CCGAGGUCCCCCCCCGAGGU

GCCCUGCCACUCUGUCAUCA**C**UGGCAGGAAAGGGGUGCUAA

GGCUGAACAAGACAUGAGCU**C**UGCCCGUACUGUCCCUGUGU

GUCCCCGCCAAGCUCCCCAC**C**GAGGUCCCCACCGAGGUUCC

AACAACAACAAAAAAUUAGC**C**AGGCGUGACUAGUCCCAGUU

CCCAGUAGGAGCCCCCGCCC**C**AGCACAGUGAACCCAGGACA

UGAGGUUCCUGCCGAAGCCC**C**AACGUGUGAUUAGGAAAGCG

CCCAGCUCCCACGUCCUCCC**C**AGACCCCCAGCUCACACCUC

GGGGUGGGAGUUCUAAUAAU**C**ACUCAUUUGCUGCUUUGCAG

GAGCUUAAGAGCAGUACACA**C**UGGAAGGCAGGGAGGGAUUG

AGGGCUUCUAGCCAAAUGAC**C**CGGGGAAAUACAGGUUACGC

ACUGAACUCUCUGUUCUGUU**C**CAUCAACUCAAAUCAACCUA

GAGGUCCCCACCGAGGUCCC**C**GCCGAGGUUCCCCCCGAGGU

GGCAGACACUCACUGCAUGC**C**UCCAGGGUGUCAGGCACCAG

CUCCCAGCCCUGUGCCUGCA**C**AAGCCUCUGCCCUGUCCCAA

CUUGAGACUCUCAGCUCCCA**C**CUCCUCCCCAGACCCCCAGC

GGUGGACCCCAGGUAGGGCU**C**CCCCAGCCCCAGGCCAGCCU

UGGGGCCUCGGUCACUGCCC**C**UGCACCAGGGCACCAAGGCC

GUCCCAGACUACUUCCUUGU**C**GCUUCUGGAAGCUUGUGCCU

CCGGCCCACCCCGGCUGCAU**C**CCUGGGGCCGGCCUGGUCCC

GCUGCGGAACGAGAGGCAGG**C**AGGUGUCCCAGACUACUUCC

AUGGCGCCUUCCCAGGUCCC**C**ACAGGCCUCUCCUCUGUGUG

GUCACUGUGGGGUCUGUCCC**C**UCCUCCACCGAGCCCCAGUG

GCGCCCCACGUCUGCUUGAC**C**CCGUAGCCCAUCACACGCAC

GUUUCUGCAGUUUCUGCCCC**C**GAGGGGCCUUGGUGGAGGCU

GGUUCACAUAUGGCGCCUUC**C**CAGGUCCCCACAGGCCUCUC

UCCUCAUCCUCUGAGGCCUU**C**UUUCCUGGGCUCUGUCCUGG

CUGUCCCCAAGCUGGGGUGC**C**CCUCACCCCACACCAAGGAG

GAGGUCCCCGCCGAGGUUCC**C**CCCGAGGUUCCCCCCGAGGU

CCCCAGCUCCCACCUCCUGC**C**AGACCCCCAGCUCACACCUC

CCCGGCUCCCAGCCCUGUGC**C**UGCACAAGCCUCUGCCCUGU

CUCCCCACCGAGGUCCCCAC**C**GAGGUUCCUGCCGAGGUCCC

AAGCCUCACGUCGCCGGCUG**C**UCUUUCAGGGGCGCCCCACG

AAGGUUCCCGCCGAGGUUCU**C**CCCGAAGUUCUGCCCGAGGU

AUCAGGAGGGAGCCAGGAAU**C**CCGACGCCGUGGGGUGGAGG

AGAGCCACUCAGCCUUCAUG**C**UCCACUUGUGAAUUCCACUC

GAAGAGGGCAAGGUGGACCC**C**AGGUAGGGCUCCCCCAGCCC

AGACCCCCAGCUCACACGUC**C**UCCCAGACCCCCAGCUCACA

ACCUCCUCCCAGACCCCUAG**C**UCACACCUCCUCCGAGACCC

ACAUGCACACGCUGACUGCC**C**UGGCUCCAGGCUCCGUCCAU

AGACCCCCAGCUCACACCUC**C**UCCUCCUGACCCCCAGCUCC

CCCAGACCCCCAGCUCAUAC**C**UCAUCCCAGACCCCCAGCUC

ACCCCCAGCUCAUACCUCAU**C**CCAGACCCCCAGCUCCCACC

CUUCUUGGUGUGAACUACAA**C**CUGGUCCUCUGAAGGUCCCU

GGCGCUCAUUCACGGAGCCC**C**CCGGGGGGCUGCACUGCACC

CCAUCUUCAGAGCCAGAGAC**C**CCCAGAUGGUGAUUCUCCCA

UGACGGACAGAAAGUCAUCC**C**CAUGGUGUGGUCACUGUGGG

UCUGUCCUGGUUCCCCAUCC**C**CUGGCAGCCUGGGAGCUCCA

UGCCCUGUCCCAAGUCAUGU**C**CUGCUUCUGUGACCCUCCUC

GUGGGGUCUGUCCCCUCCUC**C**ACCGAGCCCCAGUGCGCCAA

GUGAACCAGGGUGGGCUUCU**C**CCGCCGUCUCCUCCUGGUUC

UUCGGCUCUGAGGUUCCCCC**C**AGAGGAUCCCCCCCGAGGUU

CGUGACUAGUCCCAGUUACU**C**AGUAGGCUGGGGCAGGAGAA

GCCCGCUCCAAGGAAGCACC**C**GCCGCCUCCCAGCCAGGAUG

GACCUUGCCUGGAGUCUGAC**C**CUGAUCGGGGCCCCACGGGG

CUGCCCAGACCCCCAGCUCC**C**ACGUCCUCCCCAGACCCCCA

GAGGGAAUGUGGCCCGGCUC**C**CAGCCCUGUGCCUGCACAAG

GACCCCAGGUAGGGCUCCCC**C**AGCCCCAGGCCAGCCUUCAG

GGGCUGUCACUCCCCAGGGG**C**CAGGAGCCCCGUGUGUGGGC

UGUGGGGUCUGUCCCCUCCU**C**CACCGAGCCCCAGUGCGCCA

AUCAGAGGUAGGGACAGAUC**C**UGAGCUGGUAGUGCCAGGAA

CCAGGACAACGUCGGGGCUG**C**CUGAGUGCCAGCGCCCCUGU

GGUGGGAGUUCUAAUAAUCA**C**UCAUUUGCUGCUUUGCAGGC

AUGUGGCCCGGCUCCCAGCC**C**UGUGCCUGCACAAGCCUCUG

AAGGAGGGAAUGUGGCCCGG**C**UCCCAGCCCUGUGCCUGCAC

CCUGCGGCUGGGACGGGAUC**C**GCCUGGAGACUGCCAGCCCU

AGGGGCCUGAGGGGCCACUG**C**CUGGGGGAGCUUGGCACUGU

AAGUUCCCCCCUGAGGGCCC**C**CCAAGGUUCGGCUCUGAGGU

CAGCAGAGCCGGGACUCAAA**C**CAGGCUGCCUUCGAGUCUGG

CCCGAGGUCCCCACCGAGGU**C**CCCGCCGAGGUUCCCCCCGA

UUCAGAAAGGUUACGUAGCU**C**AGCCAAAGUCACACAGCUGG

CUCUGCAAAUGAGCUCCUGG**C**ACUGAGACCUUGCCUGGAGU

CUGAUCUGUCCCCCACUCCU**C**AAGGGACUCUCCUUGUGUGU

CCCCAGCUCACACCUCCUCC**C**AGACCCCCAGCUCACACCUC

GGGCGUGAGGGACCACAGCC**C**UGGGAGCUUGGUGCUGUGAA

CCCAUCUCCUCCCAGACCCC**C**AGCUCCCACCUCCUCCCAGA

UCCCAGACCCCCAGCUCACA**C**CUCCUCCCAGACCCCUAGCU

CCCAGACCCCCAGCUCCCAC**C**UCCCAGACCCCCAGCUCCCA

AAUCAGAGGUAGGGACAGAU**C**CUGAGCUGGUAGUGCCAGGA

AUGGCAUCAUUGCACUCCAG**C**CUGGUGAACAAGAGCAAAAC

AGGUCUUCAAUUGUUUAAAG**C**AGCUGCAUUUGGGAGCACCC

GGUUCCCGCCAGGGUCCCCG**C**CAAGGUUCCCGCCGAGGUUC

UCAGAGGCCGUGUGAGACAG**C**GAGGUCUUCAAUUGUUUAAA

GCAGCAGAGGCUGACGCUCC**C**ACCACCCUGCCUUCUGCUCC

GAGACUCUCAGCUCCCACCU**C**CUCCCCAGACCCCCAGCUCC

CCUCAGUCCCCGCCCCCAGG**C**AGUCAUACGCACACACGUGC

UCCAGCAGGGAAGGGCAAGG**C**CUGGGAUACCUCUGCCCUGG

GCUCACACGUCCUCCCAGAC**C**CCCAGCUCACAUCUCCUCCC

CGUGGGGCCCCACCUGCCAU**C**CCCCCAGUAGGGUCCUGCCC

UGCCCAGGGCCUGUCCCAGU**C**UUUCCAGCCCUGCCCAUGCU

GAGGUCCCCGCCAAGCUCCC**C**ACCGAGGUCCCCACCGAGGU

CUUGCGGGAAGCAGCUCUCA**C**ACCUGGUGAGACACUCACCU

UACAGUUGUUUGUGGUUUGU**C**UUUGGUAAUUGCCAGUUGCG

GGCCUGUCCUCAAGUACAGU**C**ACAUGCGGAGGUGCCAAGGG

CCGAGGUUCCCCCCGAGGUC**C**CCGCCAAGCUCCCCACCGAG

ACCACGGGCUGCUGAAGCAC**C**UACGGCUGGCACCUCCCUUU

UGCCAUCCCCCCAGUAGGGU**C**CUGCCCACACUGGCUCCACC

AGAAAGGUUACGUAGCUCAG**C**CAAAGUCACACAGCUGGUCU

GGGUAUGCGUGACCCCAAAA**C**AGCUGGUUAAUUCCCUGAAA

AUCUCCUCCCAGACCCCCAG**C**UCCCACCUCCUCCCAGACCC

GCCUGCGGCUGGGACGGGAU**C**CGCCUGGAGACUGCCAGCCC

UCACACGUCCUCCCAGACCC**C**CAGCUCACAUCUCCUCCCCG

ACAGAGCCCCAGUAGGAGCC**C**CCGCCCCAGCACAGUGAACC

UCCUGGGCUCUGUCCUGGUU**C**CCCAUCCCCUGGCAGCCUGG

GGGGAGACACACUCAGCCCA**C**GGCAGCAGCAGAGGCUGACG

UUGUCCCUGCGUGACGAUGU**C**CUGGUCGCCUGUGAGCUCCC

CUUGAGUGUUCGAAUGGGUA**C**CAGGCAGAUUCAGAGGGAGG

UGCACUGCACCAGGCACGGC**C**CUGAGUCCCCUGGGCUCUGC

GGCUGCUGAAGCACCUACGG**C**UGGCACCUCCCUUUCCAGAU

CUCCUCCCAGACCCCUAGCU**C**ACACCUCCUCCGAGACCCCC

CUAGCUCUAUGUGUAUGUGA**C**GGACAGAAAGUCAUCCCCAU

UGUCCCUGUGUGUGGCUGCU**C**CUCUGGGGGUCUGGGAGCGG

CCACCCAGCCAAGCCCUUCU**C**GAUUCUCAGACCUCAGAGGC

GAGACACUCACCUGUGAGGC**C**CCGGGCCUUCCUCACUCUCA

AACCCCAGCUAGCGUCCCCU**C**AGUCCCCGCCCCCAGGCAGU

GCUCCCCACCGAGGUCCCCA**C**CGAGGUUCCUGCCGAGGUCC

ACUUUUCCCCAAGGGUCCCC**C**UCUCGUUAUCACUGGAUCCU

CAGACCCCCAGCUCACACCU**C**CUCCCAGACCCCCAGCUCAC

CCUGAGACCCCCAGCUCACA**C**CUCCUCCCUGAGACCCCCAG

CUUUCCACGAAGCCUCACGU**C**GCCGGCUGCUCUUUCAGGGG

CGGGGAGGGGCCUGAGGGGC**C**ACUGCCUGGGGGAGCUUGGC

CCCAUGUCCCUGCGGUCCAG**C**GCUCCCGUGGGGCCCCACCU

CCCGUGCCGCUUUGGGGAUU**C**AGGAGAUUACGUACAGUUGU

AGGCAGGGGCCGAGUGAUGU**C**CCCCCAUGUCAGGACCAGAG

GCUGUGGGACCACAGAGCCC**C**AGUAGGAGCCCCCGCCCCAG

GUUCCAUCAACUCAAAUCAA**C**CUACAAAAAUGGCUGUAAUC

AUUCUCCCACUGCACCCUUC**C**AGGGCAAUGAACUGCACCCA

GAUUCAGAGGGAGGCAGAGC**C**AUGGGCUGCUUGGGAAUGUC

UUCCGGCCCCGCAACACAGG**C**AGUUCGCACCGCGUCUGGGG

UGGUUUCUUGCCCAGGGUAU**C**CUACCUGGGGUUCUGUCAGG

AGCCCGGCCAGCUUCCUCGU**C**GCUCCCUCCUCCUCUGCAAA

CCAUGUCCAGGUUUCCCCAC**C**ACGGGCUGCUGAAGCACCUA

GAGGCUCCCCGCCGAGGAUC**C**CCCCUCAAGGUUCCCCCCGA

CCAGCUCCCACCUCCCAGAC**C**CCCAGCUCCCACCUCCUGCC

CCCCCGAGGUCCCCGCCAAG**C**UCCCCACCGAGGUCCCCACC

ACCAUUGUUUGAGCAUUAUU**C**CUAAAGUCCCUGGGAUAUUC

UAAAAGAACAGAGCCAUUUC**C**CUCAUGCUGUUCAGCUGAGC

UGAGAGCCUCGUGAGCUGAG**C**CGAGAGCCGUGCAGGACUCA

AGAGCCUGUCUCAUUUCCUG**C**UCAUGGGAAAUACAAGUCCC

UGGGGUUUCAGCAUGUUGGC**C**AGGUUGGUCUCAAGCUCCUG

UGAGUAGCUGGAAUUACAGG**C**GCCCGCCACCACGCCCGGCU

CUGACCCCUCAUGAACAAAU**C**UCUGCCAGCUUCAAACUUUC

CUCACUACACUAAAUCAUUU**C**UUUUUUUUUCUCUCUCUCUC

UAAAGCGAGGUAUGCCUGCA**C**CGCAGGAGGCUAUUUACUUU

AACACAUUUCCAAAUGAUAU**C**CUCACAGCCCAGAGAGGAAG

CUAAAAAUACAAAAAUUAGC**C**AGGUAUGGUUGCACACACCU

UUCGAAGCCAGGCACGGAUC**C**CUUUUCUAGGAUGAAAGUCC

ACACUUUUCCUGUGUGUUCA**C**AAUUUGGCUAACUGGUAGGA

GGCGCCAUCUCGGCUCACUG**C**AAGCUCCGCCUCCCGGGUUC

GCAGUGAGCUGAGAUUGUAG**C**ACUGCACUCCAGCCGGGGCA

CCAAAUGAUAUCCUCACAGC**C**CAGAGAGGAAGCGAGUCCAA

GUCUACAUUGAAAAUCUGAU**C**UGUUUUGUUUUUGUUUUGUU

GAGGCAGGAGAUCACUUGAA**C**CCAGGAGGCAGAGGUUGCAG

GCGCCCGCCACCACGCCCGG**C**UAAUUUCUCUAUUUUUAGUA

CCCCAUCUCUACAGAAACUA**C**AAAAAUUAGCCGGGUGGUGA

AGAUCCAACGGGCAGCCAGA**C**AGCAGAGUCUGGACCACAGG

UACAGGCGUGAGCCACCGUG**C**CCGGCCUGUUUUUUUUUUCU

AUGGUGAAACCCCAUCUCUA**C**AGAAACUACAAAAAUUAGCC

UUAGUAGAGACAGGGUUUCA**C**CGUGUUAGCCAAGAUGGUCU

UCUUUGCUACAUCUUCUGCA**C**AACUGGCCGCAGCUUCUCCA

GAGGAGUGCCUGGCACAGGG**C**GGCUGCUACUAAAUGGAUAC

GUGCAAUGGCGCGAUCUCAG**C**UCACUGCAAGCUCCACCUCC

GCCCAUUUCUUUAGCCUUGG**C**UUCUUAAACAGCGUUAUCUU

ACUAAAAAUACAAAAAUCAG**C**UGGGCUUGGUGGCAGGUGCC

AAGUCCAUGGCAGUGGCUUU**C**UGGGGGUGGACGACGGGGAC

ACUCAGUGAGGAUGGGUGAC**C**UGGCCCAUGAUCCUCUGCAG

AGACCGGAGGGUGUGGGCAA**C**ACUUGGGGAGGGGAGGUGGC

UUCAAGUGAGUCUCCCACCU**C**AGACUCCUGAGUAGCUGGAA

GCAGGGAGUUAAGUUACUCG**C**CCAAGAUUUCACGGCAGUCA

UUCAAGCAAUUCUCCUGCCU**C**AGCCUCCUGAGUAGCUGGGA

GUGACUUCAGGAAAACGAAG**C**UGACUGUGGUGCAUGAGAUG

AGCUUCUCCAUCCGUACUUG**C**UGCUUCACUUUGCCUUUUUC

CUGGUCAACAUGGUGAAAGC**C**UGUCUCUACUAAAAAUACAA

CUGGGUGUUAGUUACACAAG**C**AGACAUAUGGAAAUAUUCAC

AGACUUGCAGAGUGCGAGGG**C**CUUGCUCUGGAUUGGGCUCC

GGGUGACCUGGCCCAUGAUC**C**UCUGCAGGCUCUUGCCGGCC

ACAGGUGCCUGCCAUCAUGC**C**CGGCUAAUUUUUUUGUAUUU

ACACAUUUCCAAAUGAUAUC**C**UCACAGCCCAGAGAGGAAGC

CAACCUCCGCCUCCUGGGUU**C**AAGCAAUUCUCCUGCCUCAG

AGCACUUAGCACAUAGGAAG**C**ACCCAAUCCAUCUCAGCUGC

GUAGCUGGAAUUACAGGCGC**C**CGCCACCACGCCCGGCUAAU

UAAAAGGCGGGUGUGUGGAG**C**CCAUUUCUUUAGCCUUGGCU

CUAAACUGAACCAUUUCUAG**C**UUUUGAUUUAAAGUGACAGA

GUUUCACCAUGUUGGCCAGG**C**UGGUCUUGAACUCCUGACCU

CCGGCAGCGGAGGUUCCAGC**C**CACGCUCUGGCACUUCCUAA

UUCCUCUGCAGCUUCCUCAC**C**UCUCUCAGCCUUCACAGACU

ACUCCUGGCCUCAAAGAGUC**C**UCCACUUUGGCCUCCCAAAG

CUGAGGUCAGGAGUUCGAGA**C**CAGCCUGGGCAAGAUGGUGA

AGGCGGAGGUUGCAGUGAGG**C**AAGAUUGGGCCACUGCACUC

UUAGUAAAGACAGGGUUUCA**C**CAUGUAGGCCAGGCUGAUCU

AUAGAUGAAGGUUGCCGGGU**C**CGGUCUGUGGUGCCCAAAAC

CCUGACCUCAAGUGAUCCAC**C**CACCUCGGCCUCCCACAGUG

GGUUCCAGCCCACGCUCUGG**C**ACUUCCUAACCUCUCUGUGC

AGUGAGGCAAGAUUGGGCCA**C**UGCACUCCAGCCUGGGUGAC

CAUUUCUUUAGCCUUGGCUU**C**UUAAACAGCGUUAUCUUAUA

AGCUUAGCAUUGAAUAAAGA**C**AGAAUGGGGUUUUUAUAAGC

GACUGUUUAUUUUAGGUAUG**C**ACUAGCUCAGUGGAUUUGCA

CAGGUGGGAAACCAGAUGGA**C**ACAGGUCAUCCAACCCCACA

CAAGUAGCUGAGACCACAGG**C**AUGCACAACCAUGCCUGGCU

GAUGGUCUCCAUCUCCUGAC**C**UCGUGAUCCACCCGCCUCGG

UCUUGUGUUCACUGGAGAAG**C**ACUUUGAAUUUCCUUAAAAC

GCAGGUGGAUCACCUGAGGU**C**AGAAGUUUGAGACCAGCCUG

UUCUAGACAGUCUCUAUCAC**C**CAGGCUGGAGUGCAGCGGCA

UCCAGGACACUGAAGAUGUU**C**CAUAUCUUGAUCUGGGUGUU

GCGUGGCUCAUGCCUGUCAU**C**CCAGCAAUUUGGGAGGCCAA

GAAUGCGAGGUGGCCACACA**C**UUCCAACUGGUGAAAAAAAA

ACCUCAAGUGAUCCACCCAC**C**UCGGCCUCCCACAGUGCUGG

GCUCAUUGCAGCCUCAAACU**C**CUGGGCUCAGAUGAUCCCCC

AAGGACUGGCUAAAUCCAGA**C**AUUUGUAAAAACAGUUAUGC

CUGAGAUGACAAGUGUGAGC**C**ACCGCGCCCGGCCAGUCUUC

GCGGUCCGGCAGCGGAGGUU**C**CAGCCCACGCUCUGGCACUU

ACAAGUGUGAGCCACCGCGC**C**CGGCCAGUCUUCUAUUGCAC

GGAGGCCGGCAACCCUGAAC**C**AGGGAACUGGCCAGCGGAAG

GAGUUUGAGACCAGCCUGGU**C**AACAUGGUGAAAGCCUGUCU

UCUUUAUCCGACCACUCAAA**C**UUUCUCCAUAUCAGCAGUGG

GUGAAAAUGACAUUAAAUAA**C**AUGAUGUAGGUUAAAGAACU

GGGAUCUCAGCUCACUGCAA**C**CUCCGCCUCCCAGGUUCAAG

CGUGGCUCAUGCCUGUCAUC**C**CAGCAAUUUGGGAGGCCAAG

CUGAAUAGUCAGAUUUCACG**C**ACGCUGGGAGCCACGGAUGC

UGAUCUUGAACUCCUGACCU**C**AAGUGAUCCACCCACCUCGG

UAUCUUGGCUUUCAACAAGC**C**UUCCUCACUACACUAAAUCA

UCUCUUCCUUCUGUCUCUCC**C**UCUUUCUUUCUUUUCUAGAC

CUUAGCACAUAGGAAGCACC**C**AAUCCAUCUCAGCUGCUAUU

CUCGUGAGCUGAGCCGAGAG**C**CGUGCAGGACUCAGUGAGGA

AGGAAGAGCCUGUCUCAUUU**C**CUGCUCAUGGGAAAUACAAG

CUUUUUAACCCUUGCCUUGC**C**UGUUACUUUUCUUGGAGUAA

GGAUCACAGGCACCCGCCAC**C**AUGCCCGGCUAAUUUUUGUA

GAGCGGGGAGAAGAGAGAGG**C**UGGGAAAGGCUGGUGGGCGG

AAACUGAGGACCGGAGAGAC**C**GAUAUGGAGAAGGGAAGGAC

CUGGUUCCGGGGGAACAGAC**C**CUAAGUCCAGGUGGAGACAU

GCACUGGUUUAAAGUCACCC**C**UGCACUGGCCCCUAACACUA

UUCAAGUGAUUCUCUUGCCU**C**AGCCUCCUGAGUAGCUGGGA

GGGAUUACAGGUGCCCACCA**C**CAACACCCGGCUAAUUUUUU

AGCCUCCCGAGUAGCUGGGA**C**UACAGGUGCCUGCCAUCAUG

GGCUGCCACUUCCCAGGUGC**C**ACCUGUACCAGAUCAGCCUG

AAAGUCACCCCUGCACUGGC**C**CCUAACACUAGAGUCAGCCU

UUCCUAACCUCUCUGUGCCU**C**CGAGGAGUGCCUGGCACAGG

UUCUGCACAACUGGCCGCAG**C**UUCUCCAUCCGUACUUGCUG

CCUCCUGGGUUCAAGCAAUU**C**UCCUGCCUCAGCCUCCUGAG

CAAAAUGCUGGGAUUACAGG**C**GUGAGCCACCGUGCCCGGCC
